# The proanthocyanin-related transcription factors MYBC1 and WRKY44 regulate branch points in the kiwifruit anthocyanin pathway

**DOI:** 10.1038/s41598-020-70977-0

**Published:** 2020-08-25

**Authors:** Yongyan Peng, Amali H. Thrimawithana, Janine M. Cooney, Dwayne J. Jensen, Richard V. Espley, Andrew C. Allan

**Affiliations:** 1 School of Biological Sciences, University of Auckland, 3 Symonds Street, Auckland, New Zealand; 2 The New Zealand Institute for Plant and Food Research Limited, 120 Mt Albert Road, Auckland, New Zealand; 3 The New Zealand Institute for Plant and Food Research Limited, Bisley Road, Ruakura, Hamilton, 3214 New Zealand

**Keywords:** Molecular biology, Plant sciences

## Abstract

The groups of plant flavonoid metabolites termed anthocyanins and proanthocyanins (PA) are responsible for pigmentation in seeds, flowers and fruits. Anthocyanins and PAs are produced by a pathway of enzymes which are transcriptionally regulated by transcription factors (TFs) that form the MYB-bHLH-WD40 (MBW) complex. In this study, transcriptomic analysis of purple-pigmented kiwifruit skin and flesh tissues identified *MYBC1*, from subgroup 5 of the R2R3 MYB family, and *WRKY44* (highly similar to Arabidopsis TTG2) as candidate activators of the anthocyanin pathway. Transient over-expression of *MYBC1* and *WRKY44* induced anthocyanin accumulation in tobacco leaves. Dual luciferase promoter activation assays revealed that both MYBC1 and WRKY44 were able to strongly activate the promoters of the kiwifruit *F3′H* and *F3′5′H* genes. These enzymes are branch points of the pathway which specifies the type of anthocyanin accumulated. Stable over-expression of *MYBC1* and *WRKY44* in kiwifruit calli activated the expression of *F3′5′H* and PA-related biosynthetic genes as well as increasing levels of PAs. These results suggest that while previously characterised anthocyanin activator MYBs regulate the overall anthocyanin biosynthesis pathway, the PA-related TFs, MYBC1 and WRKY44, more specifically regulate key branch points. This adds a layer of regulatory control that potentially balances anthocyanin and PA levels.

## Introduction

Anthocyanins are a group within the flavonoid family of plant secondary metabolites that determine the colour of flowers and plant organs, as well as indicator for ripeness and quality in fruit^1,2^. Flavonoids derive from phenylalanine via the general phenylpropanoid pathway which leads to different pathway branches responsible for lignins, stilbenes, condensed tannins (proanthocyanin) and anthocyanins^3^. Anthocyanin is produced by the biosynthetic pathway consisting of the commonly termed early biosynthetic enzymes, chalcone synthase (CHS), chalcone isomerase (CHI), flavanone 3-hydroxylase (F3H), flavonoid 3′-hydroxylase (F3′H), flavonoid 3′,5′-hydroxylase (F3′5′H) and the late biosynthetic enzymes dihydroflavonol 4-reductase (DFR), leucoanthocyanidin dioxygenase (LDOX), and flavonoid-3-glucosyltransferase (F3GT)^2^. Within the flavonoid family, proanthocyanins (PAs), also known as condensed tannins, are oligomers of epicatechins and catechins that are usually accumulated in the seed coats of many plants, and are well studied in the model plants *Medicago truncatala* and *Arabidopsis*^4,5^. The enzyme flavonol synthase (FLS) converts dihydroflavonol from the anthocyanin pathway into flavonols. The downstream enzymes leucoanthocyanidin reductase (LAR) and anthocyanidin reductase (ANR) are responsible for the conversion of anthocyanidin to catechin and epicatechin^6,7^. Parts of the anthocyanin and PA pathways overlap, as enzymes in the respective pathways utilise and potentially compete for the same intermediate substrates. The pathway direction is determined at the F3′H and F3′5′H branch points, two enzymes which hydroxylate the 3′, and 3′ and 5′ position of the B-ring in the carbon backbone, respectively^6,8^. The roles of F3′H and F3′5′H are critical as they determine the hydroxylation pattern of the anthocyanin aglycone, resulting in the accumulation of cyanidin and delphinidin-based anthocyanins, as well as acting as branch points that provide intermediate substrates for the PA pathway. Dihydroflavonols produced by F3′H and F3′5′H can be converted to flavonols by FLS or leucoanthocyanins by DFR, which can then be converted to the anthocyanidin aglycone by LDOX or flavanols by LAR and ANR. The substrates from the anthocyanin pathway can be intercepted by PA enzymes and diverted into producing PA instead of anthocyanin, made available by the hydroxylation of the substrate by F3′H and F3′5′H.

In addition to the biosynthesis of PAs and anthocyanins being inter-linked, both pathways are regulated by MYB-bHLH-WD40 transcription factor (TF) complexes. MYB TFs can be classified into subgroups and are responsible for many important traits in fruits and plants^9^. The MYB TFs from subgroup 6 have been implicated in the general activation of the anthocyanin pathway, such as *Arabidopsis* AtPAP1, apple MdMYB10, Petunia PH4, strawberry FaMYB10, grape VvMYBA1, kiwifruit MYB10/MYB110 and potato StMYBA1 and AN1^10–19^. Arabidopsis MYB123/TT2*,* apple MYB12, grape VvMYBPA2, peach MYB7, strawberry MYB9/MYB11 and persimmon DkMYB4 from subgroup 5 are involved in the biosynthesis of PA^5,20–24^. In addition, *Arabidopsis* MYB111/11/12, VvMYBF1 from grape, apple MdMYB22 and tomato SlMYB12 from subgroup 7 are implicated in elevating flavonol biosynthesis^20,25–27^. In contrast, MYB TFs from subgroup 4 are repressors of the flavonoid pathway such as *Arabidopsis* MYB3 and MYB4, which repress PA synthesis; FaMYB1, which represses anthocyanin and flavonol biosynthesis in strawberry; VvMYB4, which represses anthocyanin biosynthesis in grapes; and PpMYB18, which balances anthocyanin and proanthocyanin accumulation in peach^21,28–30^.

Recent research has indicated that some R2R3 MYBs from subgroup 5 may also contribute to the regulation of both anthocyanin and PA biosynthesis. Grape *VvMYB5b* encodes for a R2R3 MYB TF that clusters with PA-associated MYBs but was confirmed to activate the promoters of genes that encode enzymes of both the anthocyanin and PA pathways such as *VvLAR1, VvANS, VvANR* and *VvF3′5′H*^31^. Over-expression of *VvMYB5b* in tobacco induced accumulation of anthocyanin and PA as a result of increased expression of the tobacco versions of these genes. In poplar, *MYB6* encodes for a R2R3 MYB TF that is homologous to VvMYB5, and regulates anthocyanin and PA biosynthesis as well as lignin biosynthesis, a side branch of the general phenylpropanoid pathway^32^. In *Freesia hybrida*, the R2R3 MYB TF, FhMYB5, clusters with VvMYB5b and MYBs associated with PA regulators and not with the anthocyanin-related subgroup 6^33^. The spatio-temporal expression of *FhMYB5* correlates with both anthocyanin and PA accumulation, and its expression significantly up-regulates the expression of *F3′H*,* F3′5′H*,* DFR*,* LDOX*, in the presence of bHLH partners. In tea, CsMYB5a and CsMYB5e demonstrated the ability to regulate the accumulation of both anthocyanin and PA by activating the associated genes^34^.

The regulation of anthocyanin and PA also involves the WRKY TF family. Arabidopsis *TTG2* encodes for a WRKY TF that participates in trichome development and controls the seed coat tannins by regulating the expression of the vacuolar transporter of glycosylated epicatechin^35^. A WRKY TF from tea, CsWRKY44, is proposed to be involved in the regulation of catechin production^36^. In apple, MdWRKY11 promoted the accumulation of flavonoids and anthocyanins by regulating the MdMYB10 and MdHY5^37,38^. In petunia flower petals, the gene *PH3* encodes for a WRKY TF that is highly similar to Arabidopsis TTG2, and regulates vacuolar acidification by binding to the MBW complex that transcriptionally activates a vacuolar ATPase^39^. Further investigation has revealed that the grape VvWRKY26, a homologue to petunia PH3 and Arabidopsis TTG2, is recruited by the VvMYB5a to form a MBW-WRKY complex and enhances the transcription of a set of target genes that are involved in vacuolar hyper-acidification^40^. The VvMYB5 gene competes with the anthocyanin activator VvMYBA in the MBW complex and reduces its ability to promote anthocyanin accumulation. The recruitment of VvWRKY26 enhances the transcriptional activation of VvMYB5 towards the promoters of genes that are involved in vacuolar hyper-acidification. These studies suggest that WRKY TFs are potentially involved in anthocyanin and PA biosynthesis, via the interaction with the MBW complex.

Kiwifruit (*Actinidia* sp.) comprises over 70 species with fruits that are distinctive due to differences in colour and texture of both skin and flesh^41^. In anthocyanin-accumulating kiwifruit, two subgroup 6 MYBs, MYB10 (also called MYBF110 and MYB75, sharing 99.5% identity) and MYB110, activate the core genes of the anthocyanin pathway such as *CHS, DFR, F3GT* and *LDOX*^16–19^. Recently, a kiwifruit MYB, MYB123, which is similar to Arabidopsis TT2 in subgroup 5, demonstrated tissue-specific anthocyanin biosynthesis in the red-centred *Actinidia chinensis* by activating core genes such as *AcANS* and *AcF3GT1*^42^. In addition it was found that the microRNA miR858 targets the RNA of another subgroup 5 gene, MYBC1, in red-coloured *A. arguta*^43^. In the purple-skinned and purple-fleshed kiwifruit, MYB110 is responsible for the activation of core kiwifruit anthocyanin pathway but the regulation of the branch points *F3′H* and *F3′5′H*, which determine the types of anthocyanins accumulated, has not been elucidated^19^. Therefore, it was hypothesised that MYB110 may not be the sole regulator for anthocyanin pathway in purple kiwifruit. In order to address this gap in this study, transcriptomic analysis of purple-skinned and purple-fleshed kiwifruit was performed and revealed that MYBC1 and WRKY44 are potential regulators of the anthocyanin pathway, in addition to the previously characterised MYB110. Functional characterisation confirmed the activating roles of MYBC1 and WRKY44 on anthocyanin production, particularly the *F3′H* and *F3′5′H* branch points, adding another layer of regulatory control that is shared between anthocyanin and PA biosynthesis.

## Results

### Transcriptome sequencing of *A. purpurea*, MaMe Red and MaMe Yellow

The transcriptomic data from purple kiwifruit *A. purpurea,* MaMe Red and MaMe Yellow at three developmental stages were obtained and approximately 80% of the reads were uniquely mapped to the manually annotated *A. chinensis* genome^44^ (Supplementary Table 1). Principal component analysis (PCA) of the transcriptomic data showed that MaMe Red and MaMe Yellow grouped together according to tissue type and developmental stage, and separated from *A. purpurea* (Supplementary Fig. 1A). The two principal components explained 64% of the total variation from the 54 kiwifruit samples including three biological replicates for skin and flesh tissues sampled at mature green stage, colour change stage, and ripe stage for three kiwifruit species. The skin and flesh tissue were significantly different and separated from each other in *A. purpurea,* MaMe Red and MaMe Yellow (Supplementary Fig. 1B–D).

### Identification of differentially expressed genes (DEGs) involved in flavonoid biosynthesis pathway

**Figure 1:**
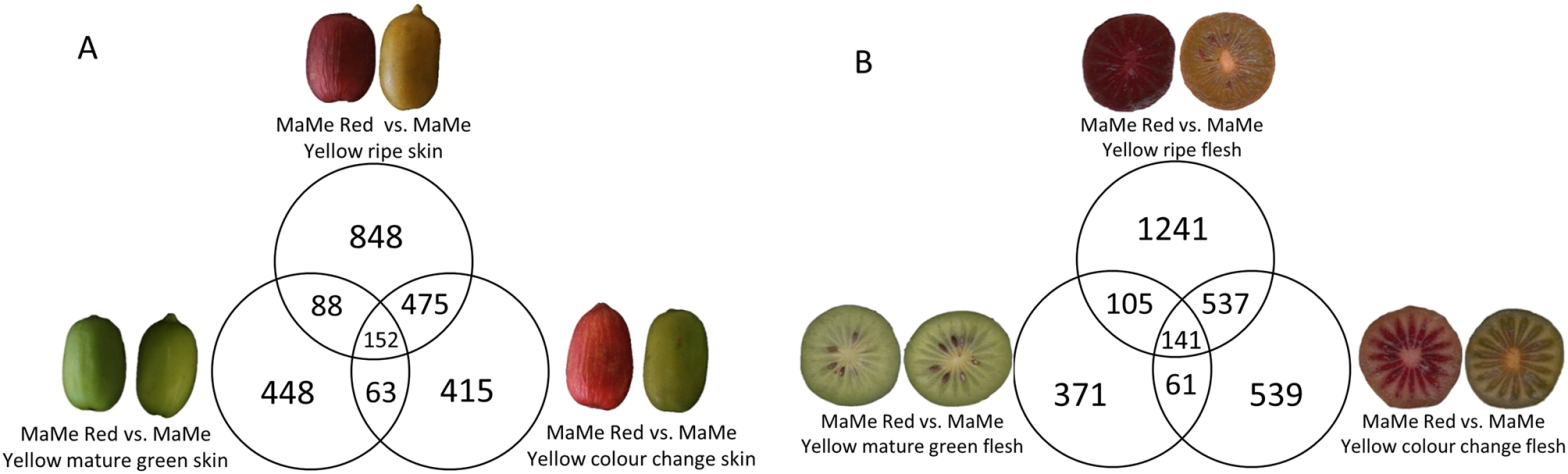
Numbers of differentially expressed genes greater than 2 Log_2_ fold-change from comparing the three developmental stages: mature green, colour change, and ripe. (**A**) Comparison of gene expression changes in the skin between MaMe Red and MaMe Yellow. (**B**) Comparison of gene expression changes in the flesh between MaMe Red and Yellow.

Skin tissue of MaMe Red was compared to that of MaMe Yellow at mature green stage, colour stage, and ripe stage to identify DEGs that may be involved in the flavonoid and anthocyanin pathways. A total of 152 genes (Supplementary Table 2) were differentially expressed more than 2 Log_2_ fold-change (Fig. 1A) between MaMe Red and Yellow, when all three maturity stages were compared. When the same comparisons were made for fruit flesh, there were 141 DEGs (Supplementary Table 3) that showed differential expression greater than 2 Log_2_ fold-change between the flesh of MaMe Red and MaMe Yellow (Fig. 1B). When overlapping the DEGs that were common between skin and flesh and present in all three maturity stages, there were 49 genes predicted to encode for biosynthetic enzymes that are potentially involved in the flavonoid and anthocyanin biosynthesis (Table 1). In addition, a shortlist of 9 gene models (skin) and 27 gene models (flesh) encoding TFs from the MYB, bHLH, homeobox and NAC families were potential flavonoid biosynthesis regulators (Table 2). The reported bHLH partner of the anthocyanin activating MYBs, termed either bHLH5 or bHLH42 (Acc19563.1)^42,45^, was not differentially expressed at higher than the 2 Log_2_ fold-change cut-off.

**Table 1:** Differentially expressed genes (DEGs) encoding biosynthetic enzymes potentially involved in the flavonoid and anthocyanin pathway obtained from the comparison between MaMe Red and MaMe Yellow skin and flesh between mature green (MG), colour change (CC), and ripe (RP) stage.

		MG	CC	RP	Base mean
**Skin comparison**					
Acc00260.1	Chalcone synthase 1	2.7	5.3	5.1	3,372
Acc02004.1	Chalcone synthase 1	2.4	5.6	6.1	4,397
Acc08970.1	Chalcone synthase 1	2.9	3.6	3.1	1,990
Acc13879.1	Anthocyanidin 3-*O*-glucoside 2″-*O*-glucosyltransferase	2.9	2.6	3.9	260
Acc16762.1	Leucoanthocyanidin dioxygenase (LDOX)	3	7.4	9.7	2,947
Acc28876.1	Leucoanthocyanidin dioxygenase (LDOX)	3.2	8.2	11.3	11,830
Acc03638.1	Chalcone–flavonone isomerase 3	0	3.2	3.8	4,242
Acc03848.1	Chalcone–flavonone isomerase	0.2	3	3.3	1,578
Acc11493.1	Flavonol synthase/flavanone 3-hydroxylase (FLS)	1.1	5.6	4.6	36
Acc20131.1	Kaempferol 3-*O*-beta-d-galactosyltransferase	1.8	8.4	9.8	4,692
Acc20132.1	Anthocyanidin 3-*O*-glucosyltransferase 2	0.7	8.5	11.2	77
Acc24966.1	Chalcone synthase 2	1.9	6.4	5.9	3,651
Acc27670.1	Chalcone–flavonone isomerase	0	2.6	2.5	214
Acc28896.1	Flavonoid 3′,5′-hydroxylase 1 (F3′5′H)	− 1	2.3	3.5	65
Acc32899.1	Leucoanthocyanidin reductase	0.5	3	3.3	189
Acc20257.1	Anthocyanidin 5,3-*O*-glucosyltransferase	8.9	3.2	5.2	31
Acc19353.1	Dihydroflavonol 4-reductase (DFR)	0.6	3.6	3.2	121
Acc26615.1	Leucoanthocyanidin reductase	− 0.4	− 2.2	− 5.6	23
Acc26709.1	Flavonol synthase/flavanone 3-hydroxylase	− 1.9	− 2.5	− 3.1	416
Acc29470.1	Isoflavone reductase homolog	3.7	10.7	12.4	117
Acc32390.1	Flavonoid 3′,5′-hydroxylase 2 (F3′5′H)	0.7	3.1	2.8	418
**Flesh comparison**					
Acc00260.1	Chalcone synthase 1	4	6.3	4.1	3,372
Acc02004.1	Chalcone synthase 1	3.7	5.1	5.2	4,397
Acc08970.1	Chalcone synthase 1	3.4	4.7	2.2	1,990
Acc16762.1	Leucoanthocyanidin dioxygenase (LDOX)	3.8	7.7	8.9	2,947
Acc20131.1	Kaempferol 3-*O*-beta-d-galactosyltransferase	5	7.1	5.9	4,692
Acc20132.1	Anthocyanidin 3-*O*-glucosyltransferase 2	6.5	8.1	7.2	77
Acc24966.1	Chalcone synthase 2	4.3	7.6	7.8	3,651
Acc28876.1	Leucoanthocyanidin dioxygenase (LDOX)	3	6.2	7.5	11,830
Acc29052.1	Isoflavone reductase homolog	5.2	8.7	12	1,645
Acc32390.1	Flavonoid 3′,5′-hydroxylase 2 (F3′5′H)	4.1	6.3	2.3	417
Acc01005.1	Dihydroflavonol 4-reductase (DFR)	0.2	4	2.5	545
Acc03638.1	Chalcone–flavonone isomerase 3	1	2.1	2.8	4,242
Acc03848.1	Chalcone–flavonone isomerase	1.7	4	3.2	1,578
Acc14022.1	Leucoanthocyanidin reductase	0.4	3.7	2.9	595
Acc18331.1	Flavonoid 3′-monooxygenase	1.4	4.1	5.1	408
Acc19353.1	Dihydroflavonol 4-reductase (DFR)	0.8	5.5	2.6	121
Acc26709.1	Flavonol synthase/flavanone 3-hydroxylase	− 1.8	− 2.4	− 2.5	416
Acc29470.1	Isoflavone reductase homolog	2.5	7.9	10.6	117
Acc32899.1	Leucoanthocyanidin reductase	0.6	4	3.9	188
Acc28896.1	Flavonoid 3′,5′-hydroxylase 1 (F3′5′H)	2.7	2.9	1.6	64

Base means are shown as an indication of expression.

**Table 2:** Differentially expressed genes (DEGs) encoding transcription factors in the MYB, bHLH, homeobox and NAC families that are possibly involved in the flavonoid and anthocyanin pathway obtained from the comparison between MaMe Red and MaMe Yellow skin and flesh between mature green (MG), colour change (CC), and ripe (RP) stage.

		MG	CC	RP	Base mean
**Skin comparison**					
Acc06303.1	Homeobox-leucine zipper protein HOX16	− 2	− 3.7	− 3.6	335
Acc10232.1	Transcription factor AtMYB114, AcMYB110	3	9.1	10.3	1,222
Acc21917.1	Transcription factor UNE10 (AtbHLH16)	5.5	5.6	4.4	10
Acc07314.1	bZIP transcription factor 44	− 0.3	2.3	2.1	226
Acc11572.1	Dof zinc finger protein DOF1.2	1.4	3.2	3.3	323
Acc22908.1	Transcription factor MYB108	1	− 2.8	− 4.4	267
Acc12965.1	Anthocyanin regulatory C1 protein, AcMYBC1	2.8	6.9	6.2	63
Acc06281.1	Transcription factor MYB1R1	− 0.2	− 2.7	− 3.1	139
Acc10227.1	Transcription factor AtMYB75, AcMYB210	− 0.2	5.4	4.6	5
Acc16887.1	WRKY transcription factor 44	− 0.1	3.4	4.2	237
**Flesh comparison**					
Acc00493.1	Transcription factor AtMYB114, AcMYB10	− 0.2	2	3.9	284
Acc10232.1	Transcription factor AtMYB114 , AcMYB110	4	9.3	11	1,222
Acc12264.1	Dof zinc finger protein DOF1.2	2	4.5	4.2	407
Acc12965.1	Anthocyanin regulatory C1 protein, AcMYBC1	7.2	8.3	8.4	63
Acc21466.1	Transcription factor bHLH149	2	3.5	2.2	41
Acc24307.1	Transcription factor MYB86 (AtMyb4)	− 5.2	− 3	− 2.1	19
Acc31401.1	Myb-related protein B (B-Myb)	− 2.7	− 2.4	− 3.2	40
Acc02780.1	WRKY transcription factor 70	− 0.2	2.7	4.1	101
Acc06319.1	Myb-related protein B (B-Myb)	− 0.1	− 2.8	− 3.3	55
Acc06321.1	Homeobox-leucine zipper protein ATHB-13	0.5	− 3.3	− 2.8	83
Acc07314.1	bZIP transcription factor 44	0.1	2	2.1	226
Acc08333.1	Transcription factor bHLH14	− 1.9	− 2.8	− 2.1	12
Acc10227.1	Transcription factor AtMYB75, MYB210	0.1	5.3	4.3	5
Acc10925.1	Transcription factor MYB86 (AtMyb4)	0.5	− 2.9	− 3.6	14
Acc11572.1	Dof zinc finger protein DOF1.2	0	2.1	2.6	323
Acc13277.1	Myb-related protein 306	0	− 2.9	− 4.9	10
Acc13287.1	Cyclic dof factor 3 (AtDOF3.3)	1.2	− 2.5	− 2	8
Acc14650.1	NAC domain-containing protein 21/22	− 0.1	− 4.6	− 3.7	11
Acc15830.1	Transcription factor MYB1R1	0.4	− 2.2	− 2.6	1,320
Acc16026.1	Anthocyanin regulatory C1 protein, MYB12-like	1.5	4	4.1	7
Acc16887.1	WRKY transcription factor 44	1.8	4.8	5.2	237
Acc19793.1	WRKY transcription factor 53	− 0.4	− 3.2	− 2.2	330
Acc20254.1	WRKY transcription factor 27	1	− 3.4	− 2.3	24
Acc23052.1	Myb-related protein 306	0	− 3.7	− 3.3	115
Acc23779.1	Transcription factor MYB44	− 1	− 2.4	− 2.9	143
Acc28877.1	Transcription factor bHLH120	− 0.9	− 4.4	− 8.8	15
Acc30637.1	Homeobox-leucine zipper protein HAT5	− 0.3	− 2.3	− 2.6	266
Acc06303.1	Homeobox-leucine zipper protein HOX16	− 3.4	− 2.6	− 0.3	335
Acc29560.1	WRKY transcription factor 40	2	3.1	1.2	307
Acc24555.1	bZIP transcription factor 53	2.3	− 0.6	− 2.6	29

Base means are shown as an indication of expression.

**Figure 2:**
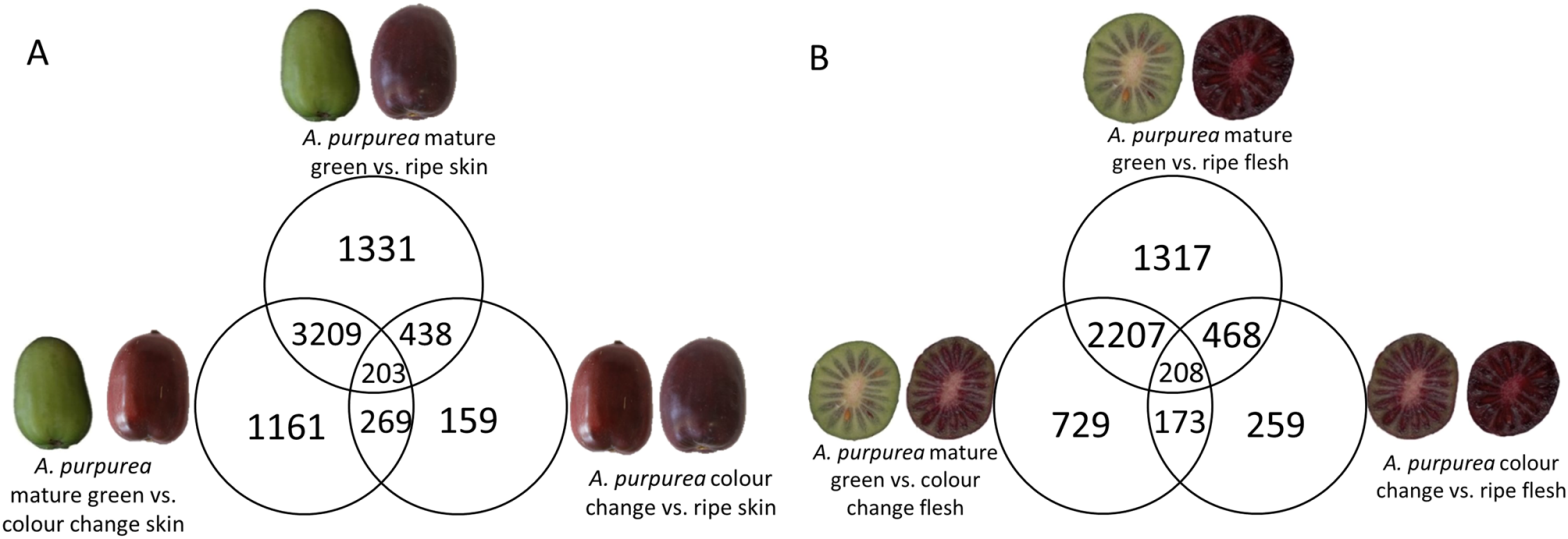
Numbers of differentially expressed genes greater than 2 Log_2_ fold-change from comparing the three developmental stages: mature green, colour change, and ripe. (**A**) Comparison of gene expression changes in the skin of *A. purpurea*. (**B**) Comparison of gene expression changes in the flesh of *Actinidia purpurea*.

To validate these observations against an independent genetic background, a comparison of fruit skin and flesh of the related species *A. purpurea* was made. Comparison between skin and flesh maturity time points revealed 203 DEGs consistently changing during ripening in skin (Fig. 2A, Supplementary Table 4) and 208 DEGs in flesh (Fig. 2B, Supplementary Table 5). Forty gene models and 26 gene models identified in the skin and flesh comparisons of *A. purpurea,* respectively, were predicted to encode for biosynthetic enzymes in the flavonoid and anthocyanin pathways (Table 3). In the same comparisons, 24 TFs and 94 TFs were identified from the MYB, bHLH, homeobox and NAC families that may be involved in the flavonoid and anthocyanin biosynthesis in skin and flesh of *A. purpurea,* respectively (Supplementary Table 6).

**Table 3:** Differentially expressed genes (DEGs) encoding biosynthetic enzymes potentially involved in the flavonoid and anthocyanin pathway obtained from the comparison between mature green (MG), colour change (CC), and ripe (RP) stage in *Actinidia purpurea* skin and flesh.

		MG-RP	MG-CC	CC-RP	Base mean
**Skin comparison**					
Acc00260.1	Chalcone synthase 1	− 1.6	− 3	1.5	3,372
Acc02004.1	Chalcone synthase 1	− 2.7	− 3.8	1.1	4,396
Acc03410.1	UDP-glycosyltransferase 88B1	2.4	2.3	0.1	101
Acc03638.1	Chalcone–flavonone isomerase 3	0.8	− 1.3	2	4,241
Acc03848.1	Chalcone–flavonone isomerase	− 1.2	− 2.1	0.9	1,577
Acc05968.1	Putative glycosyltransferase 7 (AtGT7)	2.3	0.7	1.6	175
Acc08970.1	Chalcone synthase 1	− 1.6	− 3.1	1.5	1989
Acc12813.1	Flavonoid 3′-monooxygenase	4.1	4.3	− 0.1	437
Acc13879.1	Anthocyanidin3-*O*-glucoside2″-*O*-GT	− 0.6	− 3.1	2.5	260
Acc14022.1	Leucoanthocyanidin reductase	− 1.9	− 3.9	2	594
Acc16762.1	Leucoanthocyanidin dioxygenase (LDOX)	− 1.3	− 3.6	2.4	2,947
Acc18331.1	Flavonoid 3′-monooxygenase	4.3	1.7	2.6	408
Acc19922.1	Flavonoid 3′,5′-hydroxylase 1 (F3′5′H)	4.7	4.4	0.3	97
Acc20131.1	Kaempferol 3-*O*-beta-d-galactosyltransferase	− 1.4	− 2.9	1.5	4,691
Acc20132.1	Anthocyanidin 3-*O*-glucosyltransferase 2	− 1.4	− 2.8	1.4	77
Acc20478.1	UDP-glycosyltransferase 73C3	− 2.3	− 2.4	0	1,379
Acc21858.1	Leucoanthocyanidin reductase	4.8	3.6	1.2	100
Acc23638.1	Isoflavone reductase-like protein	− 3.5	− 2.8	− 0.7	1,013
Acc23730.1	Putative dihydroflavonol 4-reductase (DFR)	2	1.3	0.7	231
Acc24966.1	Chalcone synthase 2	− 2.6	− 3.5	0.9	3,651
Acc27670.1	Chalcone–flavonone isomerase	− 1.3	− 2.1	0.8	214
Acc28876.1	Leucoanthocyanidin dioxygenase (LDOX)	− 1.3	− 3.2	1.9	11,830
Acc28896.1	Flavonoid 3′,5′-hydroxylase 1 (F3′5′H)	2.1	0.6	1.5	64
Acc32899.1	Leucoanthocyanidin reductase	− 1.4	− 3.6	2.2	188
**Flesh comparison**					
Acc00260.1	Chalcone synthase 1	− 2.6	− 3.9	1.3	3,372
Acc02004.1	Chalcone synthase 1	− 4.5	− 4.7	0.2	4,396
Acc02411.1	UDP-glycosyltransferase 90A1	3.6	2	1.6	111
Acc02866.1	UDP-glycosyltransferase 76F1	4.5	3.5	1	172
Acc03410.1	UDP-glycosyltransferase 88B1	3.4	3	0.5	101
Acc06429.1	UDP-glycosyltransferase 89A2	− 4.7	− 4.8	0.1	3,379
Acc08970.1	Chalcone synthase 1	− 3	− 4.5	1.6	1,989
Acc12813.1	Flavonoid 3′-monooxygenase	6	5.3	0.7	437
Acc14022.1	Leucoanthocyanidin reductase	− 2.3	− 3.3	1	594
Acc16762.1	Leucoanthocyanidin dioxygenase (LDOX)	− 3.1	− 3.7	0.7	2,947
Acc16896.1	UDP-glycosyltransferase 73E1	− 2.7	− 0.7	− 2	243
Acc18331.1	Flavonoid 3′-monooxygenase	4.1	2.1	2	408
Acc21858.1	Leucoanthocyanidin reductase	3.8	3.2	0.6	100
Acc23638.1	Isoflavone reductase-like protein	− 2.4	− 2.2	− 0.2	1,013
Acc24966.1	Chalcone synthase 2	− 5.3	− 5.6	0.3	3,651
Acc25835.1	UDP-glucose iridoid glucosyltransferase	3.2	3.9	− 0.7	865
Acc26709.1	Flavonol synthase/flavanone 3-hydroxylase	3.2	2.1	1.1	416
Acc32899.1	Leucoanthocyanidin reductase	− 2.3	− 3.4	1.1	189

Base means are shown as an indication of expression.

The common DEGs that appeared on all lists between the skin and flesh comparisons of MaMe Red, MaMe Yellow and *A. purpurea* are gene models encoding CHS (Acc00260.1, Acc02004.1, Acc08970.1, and Acc24966.1), LDOX (Acc16762.1), and LAR (Acc32889.1). Additionally, the two DEGs that are shared in all four lists encodes for the TFs bZIP44 (Acc07314.1) and MYB110 (Acc10232.1). The lack of *CHS* and *F3GT* expression has been associated with the lack of anthocyanin accumulation and the TF MYB110 (Acc10232.1) has been shown to be responsible for anthocyanin biosynthesis in these purple kiwifruit species^19^. The CHS (Acc08970.1), CHI (Acc03638.1), F3′H (Acc12813.1 and Acc18331.1), F3′5′H (Acc32390.1), LDOX (Acc28876.1), flavonoid 3′ glycosyltransferase F3GT1 (anthocyanin glucosyltransferase Acc20132.1) and the TF MYB10 (Acc00493.1) and MYB110 (Acc10232.1) were previously characterised in purple kiwifruit.

### Candidate TFs MYBC1 and WRKY44 identified from DEGs

**Figure 3:**
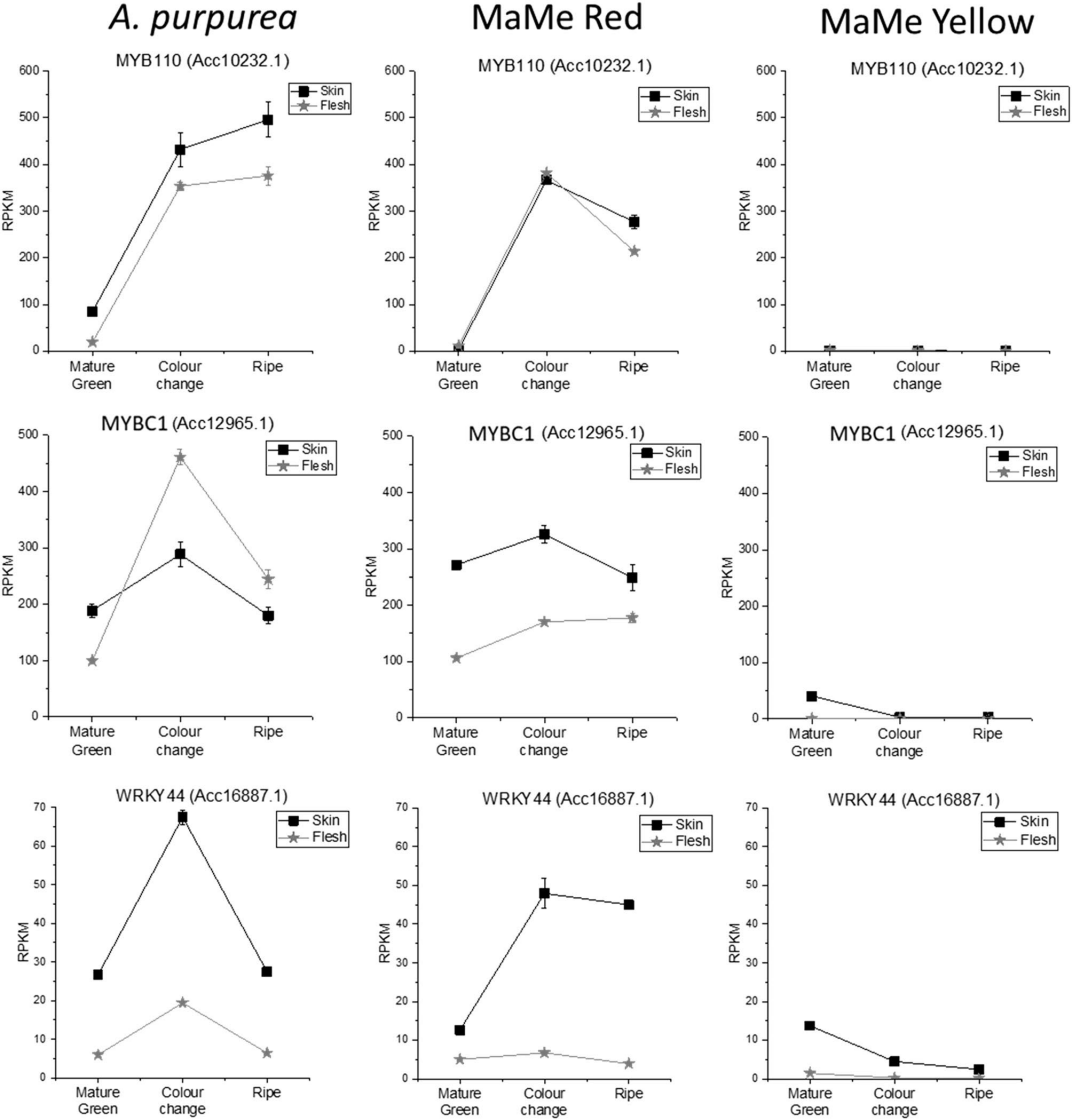
Expression of *MYB110* (Acc10232.1), *MYBC1* (Acc12965.1) and *WRKY44* (Acc16887.1) in the skin and flesh of *Actinidia purpurea*, MaMe Red, and MaMe Yellow across the three developmental stages. Gene expression was expressed in Reads per Kilobase per Million (RPKM). Data were shown as means ± SEM of three biological replicates.

The TF MYB110 (Acc10232.1) was identified in all DEG lists confirming previous studies which have identified its role in elevating anthocyanin biosynthesis^16,19,46^. MYB10 (Acc00493.1) appears to have a more important role in the kiwifruit species, *A. chinensis*^17^, and was identified as a DEG in flesh samples. The MYB TF MYBC1 (Acc12965.1)^43^ and the WRKY TF termed WRKY44 (Acc16887.1) appeared in all the DEG lists of comparisons between MaMe Red and MaMe Yellow. The expression of *MYBC1* exhibited high fold-changes during the colour change and ripe stage compared to the mature green stage in both the skin and flesh (Table 2). *MYBC1* was highly expressed during the colour change and ripe stage in the purple coloured *A. purpurea* and MaMe Red, but barely detected in MaMe Yellow (Fig. 3). Similarly, *WRKY44* was highly expressed in the *A. purpurea* and MaMe Red during the colour change and ripe stage but very lowly expressed in the anthocyanin lacking MaMe Yellow. The expression of *WRKY44* exhibited high fold change increases in both the skin and flesh during colour development (Table 2).

The expression of *MYBC1* and *WRKY44* was significantly higher in coloured fruit, as was *MYB110,* correlating with anthocyanin accumulation. There was some reduction in expression in ripe fruit. The expression of other subgroup 5 MYBs was very low (< 20 RPKM) and the expression patterns did not correlate with the expression of *MYB110* or with anthocyanin accumulation (Supplementary Fig. 2). MYBC1 (closest *A. chinensis* gene model, Acc12965.1) has been implicated in the control of *A. arguta* anthocyanin levels^43^. Another TT2-like MYB termed AcMYB123 (closest gene model Acc28234.1) was proposed to regulate the anthocyanin accumulation in the inner pericarp of the red-centred *A. chinensis*^42^. In our RNA-seq data, the expression of AcMYB123 (Acc28234.1) and other TT2-like MYBs was barely detected in either skin or flesh (Supplementary Fig. 2). Also, the low expression of other *WRKY44-like* gene models did not indicate any correlation with anthocyanin accumulation (Supplementary Fig. 3). Therefore, MYBC1 (Acc12965.1) and WRKY44 (Acc16887.1) from purple kiwifruit *A. melanandra, A. purpurea,* and MaMe Red were selected as candidate genes for functional characterisation.

### MYBC1 is a subgroup 5 R2R3 MYB and WRKY44 belongs to group I of the WRKY family

**Figure 4:**
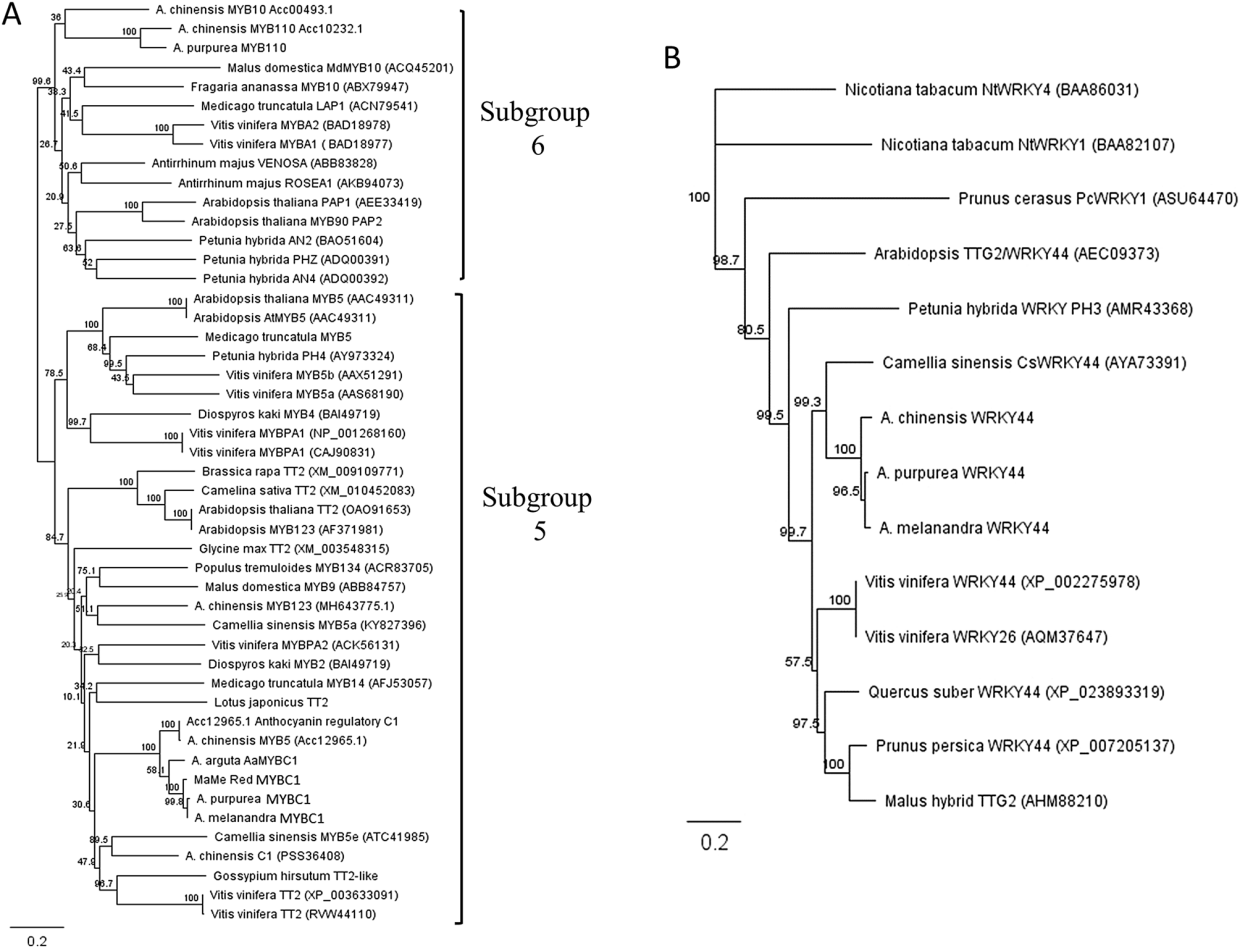
Phylogeny trees of deduced amino acid sequences alignments constructed by neighbour-joining method in Geneious 10.0.3. using global alignment with free end gaps and the protein distance was calculated by Jukes-Cantor model. (**A**) Phylogenetic relationship of MYBC1 from *Actinidia melanandra, A. purpurea* and MaMe Red with R2R3 MYB TFs from subgroup 5 and subgroup 6 from other plant species. (**B**) Phylogenetic relationship of WRKY44 from *A. melanandra* and *A. purpurea* WRKY TFs from other plant species using *Nicotiana tabacum* NtWRKY44 (group III) as outgroup.

Phylogenetic analysis of MYBC1 from *A. melanandra* (purple-skinned and purple-fleshed)*, A. purpurea,* and MaMe Red suggested a close relationship with TT2 TFs from a variety of plant species (Fig. 4A). Using R2R3 MYB TFs from the anthocyanin-related subgroup 6 and subgroup 5 in the phylogenetic tree revealed that kiwifruit MYBC1 clustered with *Gossypium* TT2 and *Camellia sinensis* MYB5a, which belong to subgroup 5 of the R2R3 MYB TF family involved in proanthocyanin biosynthesis^34^. Deduced amino acid alignments revealed that MYBC1 from *A. melanandra, A. purpurea*, and MaMe Red shared 98.2% identity and belonged to the R2R3 MYB TF family, as indicated by the highly conserved R2R3 domains (Supplementary Fig. 4). Although grouped with the PA regulators from subgroup 5, the C1 motif was not observed in the kiwifruit MYBC1 sequences, whereas some amino acids in the C3 motif and the VIRTKAx[K/R]C motif common in the PA regulators were observed in kiwifruit MYBC1 deduced amino acid sequences^31,34^. Similar to previously identified AcMYB123 (Acc28134.1), kiwifruit MYBC1 clustered with the TT2 clade and belongs to the subgroup 5 of the R2R3 MYB TF family in plants.

Phylogenetic analysis of WRKY44 from *A. melanandra* and *A. purpurea* revealed similarity with WRKY44/TTG2 TFs from a range of plant species (Fig. 4B). Kiwifruit WRKY44 grouped into the TTG2 clade and close to the *Camellia sinensis* WRKY44 which is a regulator of PA biosynthesis^36^. Kiwifruit WRKY44 possesses two WRKY domains composed of the conserved amino acids WRKYGQK toward the N-terminal as well as a zinc-finger ligand and a potential nuclear localisation sequence (Supplementary Fig. 5)^47^. These results suggested that the kiwifruit WRKY44 belongs to the group I of the WRKY superfamily in plants.

### MYBC1 and WRKY44 induced anthocyanin patches on the leaves of *Nicotiana tabacum*

**Figure 5:**
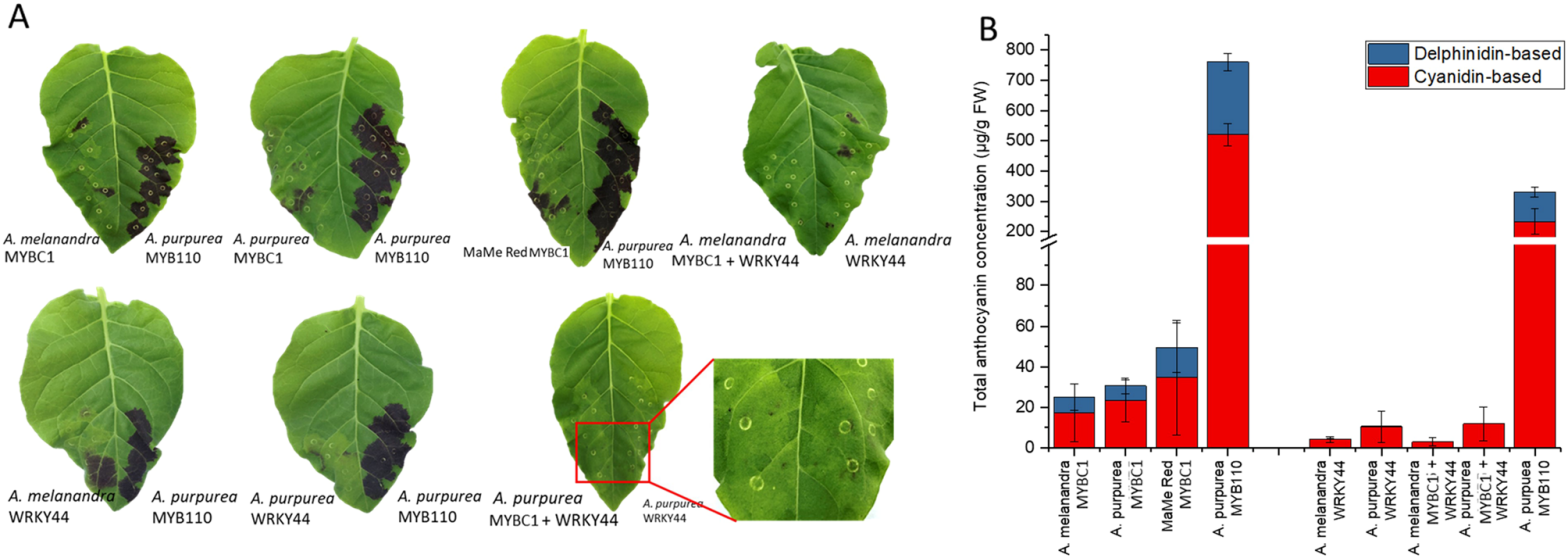
Transient over-expression of *MYBC1* and *WRKY44* in *Nicotiana tabacum* leaves for anthocyanin accumulation. (**A**) Digital image of *N*. *tabacum* leaves 7 days after transient over-expression of *MYBC1* and *WRKY44* from *Actinidia melanandra, A. purpurea* and MaMe Red with *MYB110* from *A. purpurea* as a positive control. (**B**) HPLC measurement of anthocyanin from transient over-expression of *MYBC1* and *WRKY44* from (**A**). Data were shown as means ± SEM of three individual experiments.

Transient over-expression of kiwifruit *MYBC1* and *WRKY44* in the leaves of *N. tabacum* induced anthocyanin accumulation (Fig. 5A). However, anthocyanin accumulated unevenly around the infiltration sites, as opposed to the complete saturation of anthocyanin at the infiltration site using the positive control, *A. purpurea MYB110*. The formation of the anthocyanin patches was variable between leaves. In some cases, only small points of anthocyanin accumulation formed near the infiltrated sites. The majority of anthocyanins induced by transient over-expression of *MYBC1* are cyanidin-based and the remainder are delphinidin-based anthocyanins, whereas only cyanidin-based anthocyanin was observed from the transient over-expression of *WRKY44* (Fig. 5B). These results suggest that both *MYBC1* and *WRKY44* from the purple kiwifruit species are able to induce anthocyanin accumulation when transiently over-expressed in tobacco leaves. The patchy and variable anthocyanin formation around the infiltration sites may indicate that MYBC1 and WRKY44 may require additional TF partners to fully activate the anthocyanin pathway.

### MYB and WRKY binding motifs annotated on kiwifruit *F3′H* and *F3′5′H* promoters

**Figure 6:**
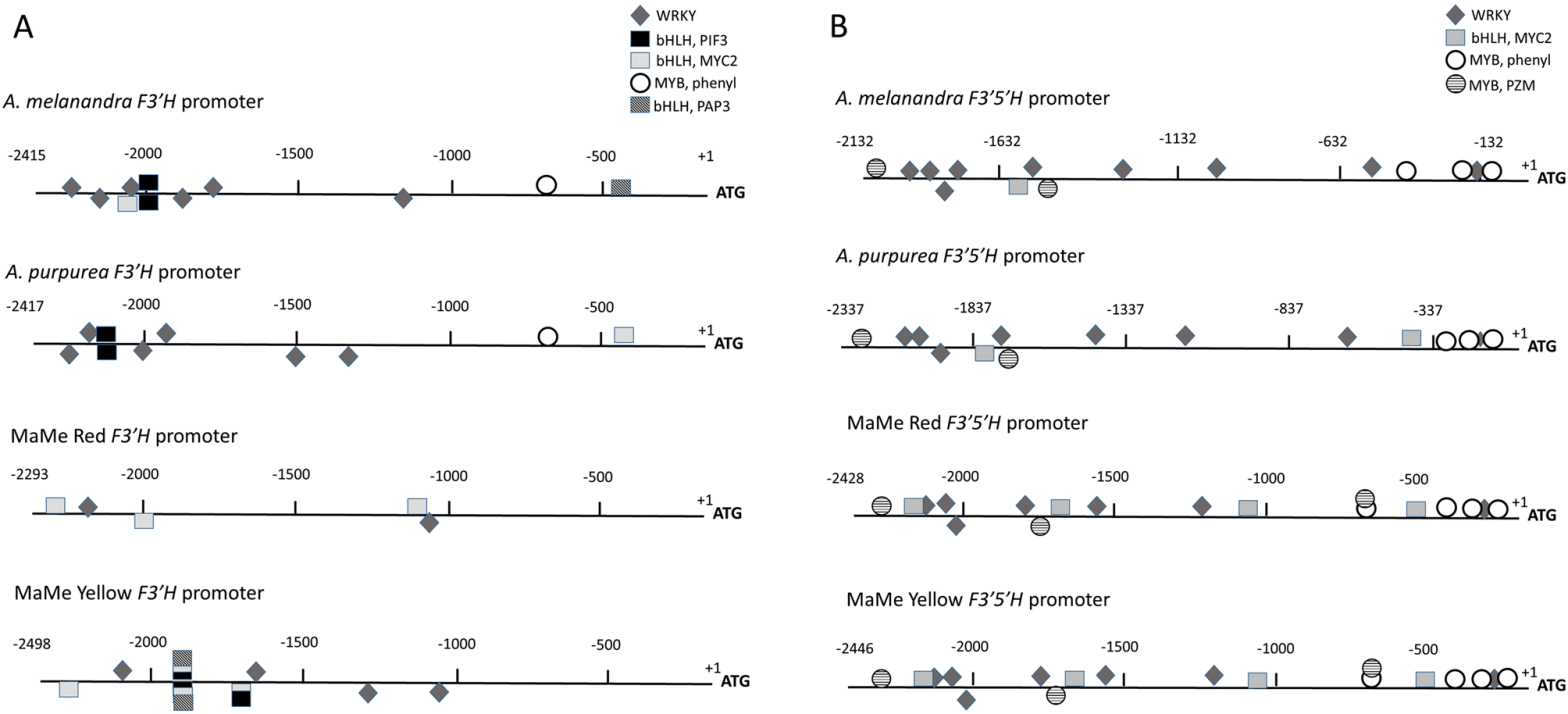
Predicted binding sites of phenylpropanoid-related transcription factor on *Actinidia melanandra, A. purpurea*, MaMe Red and MaMe Yellow promoters using PlantPAN3.0. (**A**) Annotation of WRKY, bHLH, and MYB transcription factors related with phenylpropanoid pathway on *F3′H* promoters. (**B**) Annotation of WRKY, bHLH, and MYB transcription factors related with phenylpropanoid pathway on *F3′5′H* promoters. WRKY: binding site for WRKY, AtTTG2; bHLH, PIF3: binding site for bHLH AtPIF3; bHLH, MYC2: binding site for bHLH AtMYC2; MYB, phenyl: MYB binding motif found in promoters of phenylpropanoid biosynthetic genes. MYB, PZM: core consensus binding site for maize P gene (MYB homologue). The binding sites are displayed on the top (positive strand) and bottom (negative strand) have a similar score to the HIT sequence of 1.

Potential binding motifs for the TFs from MYB, bHLH and WRKY families were predicted to be present on the *F3′H* and *F3′5′H* promoters previously cloned from *A. melanandra, A. purpurea,* MaMe Red and MaMe Yellow by the online tool PlantPAN3.0^19,48^. Further screening of TF binding sites and motifs, by only including HIT score of 1, revealed predicted binding sites for MYB, bHLH, and WRKY TFs that are related to the phenylpropanoid pathway and flavonoid biosynthesis (Fig. 6). The common TF binding motifs found on all *F3′H* promoters were for bHLH TF, AtMYC2 and a WRKY TF, AtTTG2 (Fig. 6A). In addition, within the *A. melanandra* and *A. purpurea F3′H* promoters, a binding site for another bHLH TF, AtPIF3, and a binding site for MYB (MYB, phenyl) that binds to the promoters of phenylpropanoid biosynthetic genes were identified. All four *F3′5′H* promoters harboured the binding motifs for the bHLH TF, AtMYC2, the WRKY TF, AtTTG2, the MYB (MYB, PZM) which is the core consensus for the anthocyanin-related *P* gene in maize, and the MYB-binding motif found in the promoters of phenylpropanoid biosynthetic genes (Fig. 6B). The presence of these predicted binding sites within the *F3′H* and *F3′5′H* promoters suggests regulation by flavonoid-related TFs.

### MYBC1 and WRKY44 activated kiwifruit *F3′H* and *F3′5′H* promoters

**Figure 7:**
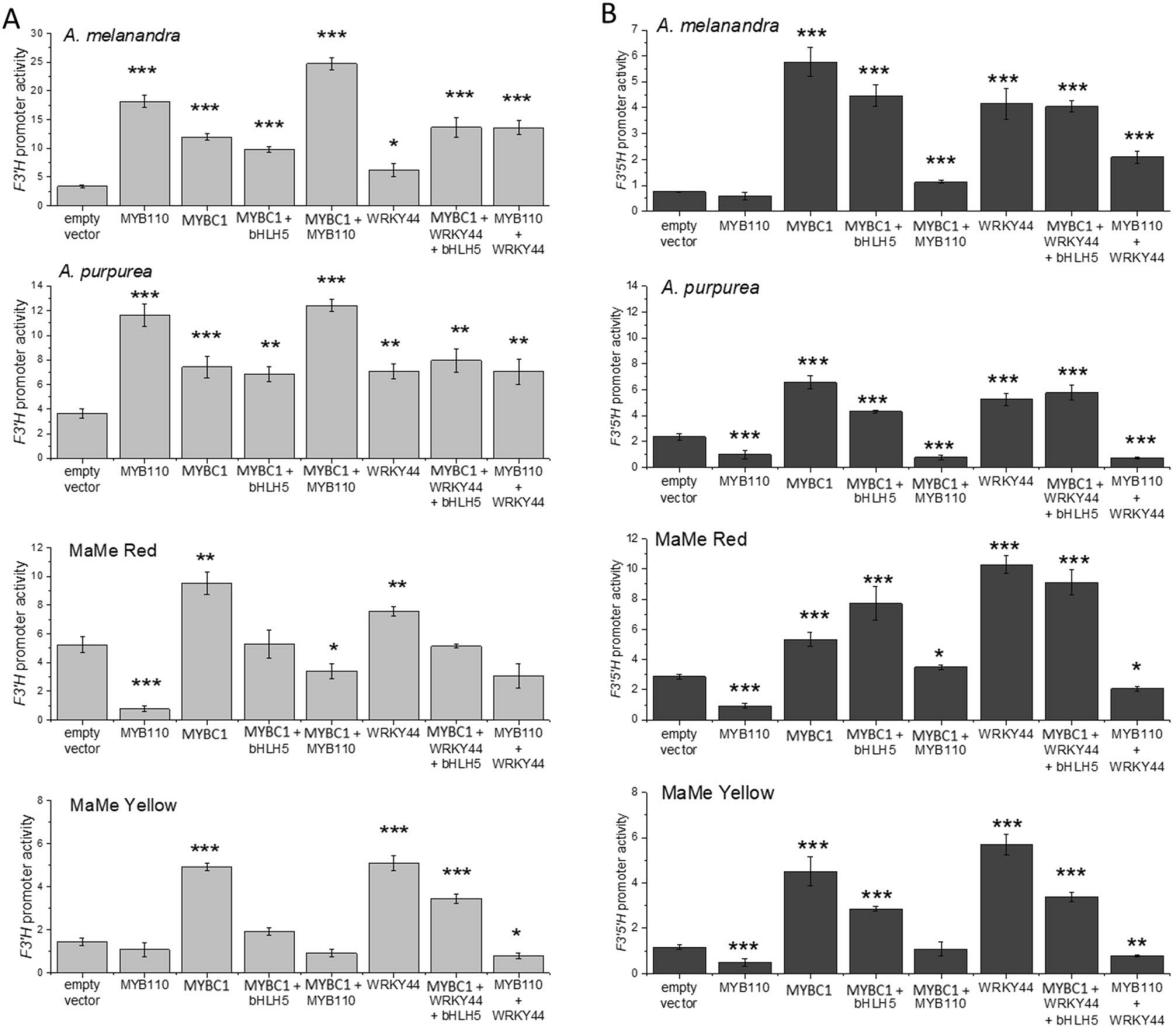
Dual luciferase promoter activation assays testing the ability of MYBC1 and WRKY44 for promoter activation. (**A**) *F3′H* promoters isolated from *Actinidia melanandra*, *A. purpurea*, MaMe Red and MaMe Yellow were tested for activation by the *MYBC1* and *WRKY44* cloned from the respective species. (**B**) *F3′5′H* promoters isolated from *A. melanandra*, *A. purpurea*, MaMe Red and MaMe Yellow were tested for activation by the *MYBC1* and *WRKY44* cloned from the respective species. Empty vector and *A. purpurea MYB110* were included as control. Data were shown as means ± SEM of four biological replicates. Statistical significance against the empty vector control: p < 0.05*, p < 0.01**, and p < 0.001***.

Dual luciferase promoter activation assays in the leaves of *N. benthamiana* showed that *MYBC1* and *WRKY44* significantly activated the *F3′H* promoters from all four species, including MaMe Yellow (Fig. 7A). There were high endogenous promoter readings, again suggesting activation by endogenous tobacco TFs. Previously it has been shown that *MYB110* only activated the *F3′H* promoters from *A. melanandra* and *A. purpurea,* but not the MaMe kiwifruits^19^. The results here suggested that *MYBC1* and *WRKY44* were able to activate all *F3′H* promoters including those cloned from MaMe kiwifruits. However, no activation was observed by MYB110 on the MaMe *F3′H* promoters with or without *MYBC1* and/or *WRKY44* co-infiltration.

Infiltrations of *MYBC1* and *WRKY44* significantly activated *F3′5′H* promoters from all four species, with and without the co-factor *bHLH5* (Acc19563.1) (Fig. 7B). As with *F3′H* promoters, there were high endogenous promoter readings. Consistent with previous findings, *MYB110* was unable to activate *F3′5′H* promoters from all four kiwifruit species^19^. However, co-infiltration of *MYBC1* or *WRKY44* with *MYB110* increased the activity of *A. melanandra F3′5′H* promoter and elevated the MaMe Red *F3′5′H* promoter when *MYBC1* was co-infiltrated with *MYB110.* In summary, both *MYBC1* and *WRKY44* were able to significantly activate the *F3′H* and *F3′5′H* promoters from all four kiwifruit species, above an already high endogenous activity.

### Over-expression of *MYBC1* and *WRKY44* in *A. arguta* increases *F3′5′H* expression and PA content

**Figure 8:**
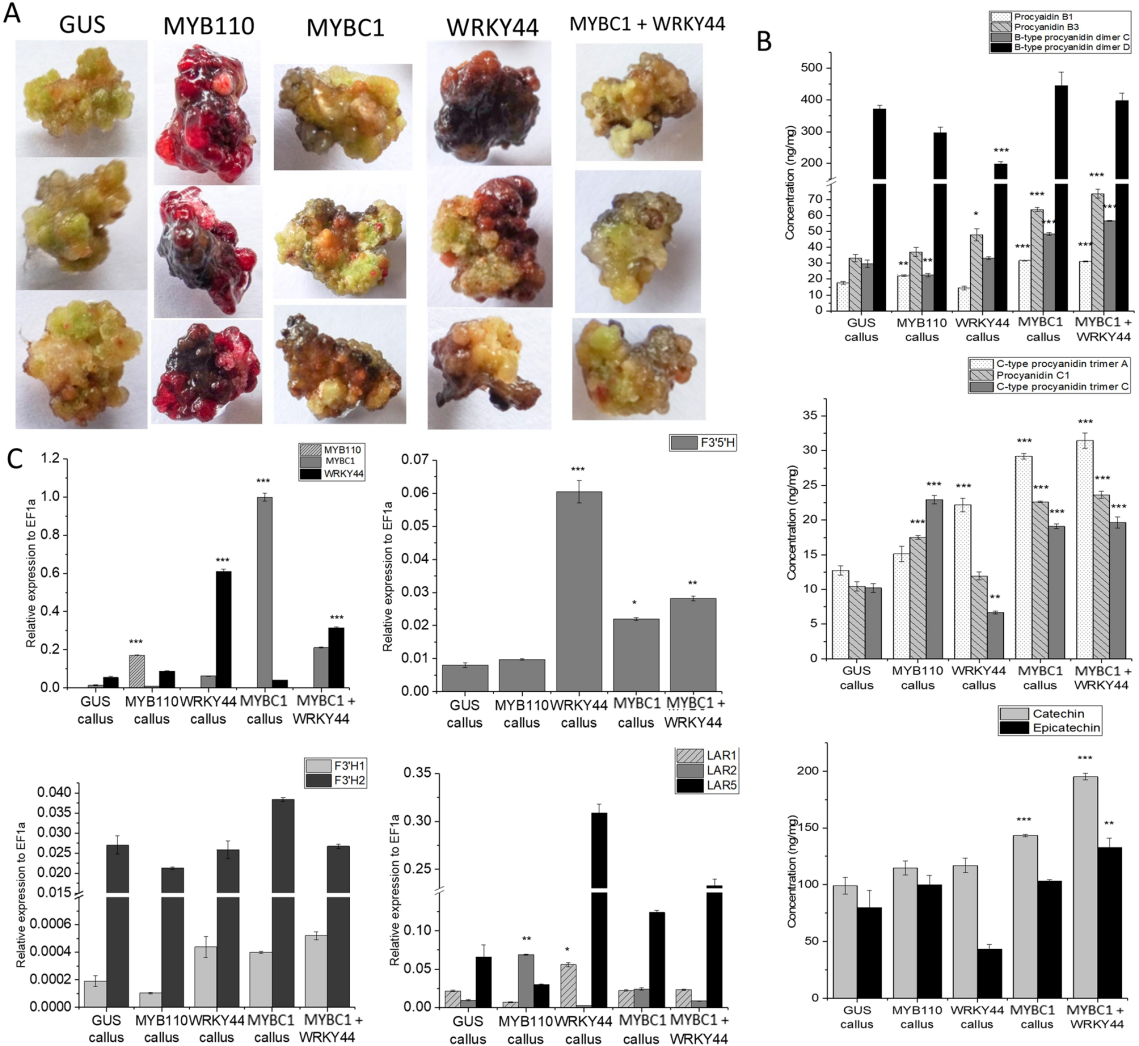
Stable over-expression of *GUS, MYB110, MYBC1,* and *WRKY44* in *Actinidia arguta.* (**A**) Callus formation of *GUS, MYB110, MYBC1, WRKY* and the co-infiltration of *MYBC1*/*WRKY44* eight weeks after *Agrobacteria-*mediated over-expression in *A. arguta* leaf explant. (**B**) Proanthocyanin and anthocyanin analysis of the calli sampled from (**A**). (**C**) Gene expression analysis of the transgenes *MYB110, MYBC1, WRKY44* and genes involved in the proanthocyanin and anthocyanin pathway in the callus from stably transformed calli. Data were shown as means ± SEM of three biological replicates. Statistical significance against the *GUS* control: p < 0.05*, p < 0.01**, and p < 0.001***.

Transgenic calli were produced which over-expressed *GUS, MYB110, MYBC1, WRKY44* and *MYBC1/WRKY44* co-expression. These were analysed for gene expression and metabolite composition (Fig. 8A). *GUS* and *MYB110* were transformed in *A. arguta* ‘K2D4’ as negative and positive controls, respectively. Phenotypically, *MYB110* over-expressing calli are intensely purple throughout the tissue due to the high concentration of cyanidin-based anthocyanin, which is the only anthocyanin type detected (Supplementary Fig. 6A). The phenotype of calli over-expressing *GUS* showed no distinctive difference to the slightly more pigmented calli over-expressing *MYBC1* and *WRKY44*. However, metabolite analysis revealed that *MYBC1* and *MYBC1/WRKY44* co-expression calli accumulated significantly more procyanidin B1, B3, B-type procyanidin dimer C, procyanidin C1, C-type procyanidin trimer A and C, and catechin (Fig. 8B) compared to the *GUS* and *MYB110* over-expressing calli (see categories of PA^49^). *WRKY44* over-expressing calli accumulated significantly higher levels of procyanidin B3 and C-type procyanidin trimer A than the control calli but a significant decrease in B-type procyanidin dimer D and C-type procyanidin trimer C (Fig. 8B). The accumulation of epicatechin and catechin were highest in calli co-expressing *MYBC1/WRKY44*. There were also small amounts of cyanidin-based anthocyanin detected in *MYBC1* and *MYBC1/WRKY44* calli. Plantlets regenerated from *MYB110* over-expressing callus showed purple pigmentation throughout the shoots and roots, whereas the plantlets regenerated from *MYBC1* and *MYBC1*/*WRKY44* showed purple pigmentation in the roots only, compared to the green plantlets regenerated from the *GUS* control (Supplementary Fig. 7).

Gene expression analysis revealed *MYB110* over-expressing calli had high expression of the *F3GT* gene (Acc20132.1) in comparison to other calli (Supplementary Fig. 6B). Expression levels of *DFR*, *F3′H1* and *F3′H2* (Acc01005.1, Acc12813.1 and Acc18331.1) were similar across all transformed calli. Noticeably, the expression of *F3′5′H* (Acc32390.1) was significantly elevated in the calli over-expressing *WRKY44, MYBC1* and a combination of both, compared to the *GUS* and *MYB110* controls. Expression of the PA-related genes, *FLS1* and *LAR1* was elevated in the *WRKY44* calli, whereas *FLS2* was elevated in calli over-expressing GUS and MYB110 controls (Fig. 8C, Supplementary Fig. 6B). *LAR5* was elevated in calli transformed with *MYBC1* and *WRKY44*. Expression of both *ANR1* and *ANR2* was elevated in the calli over-expressing *WRKY44, MYBC1* and *MYBC1/WRKY44* (Supplementary Fig. 6B).

**Figure 9:**
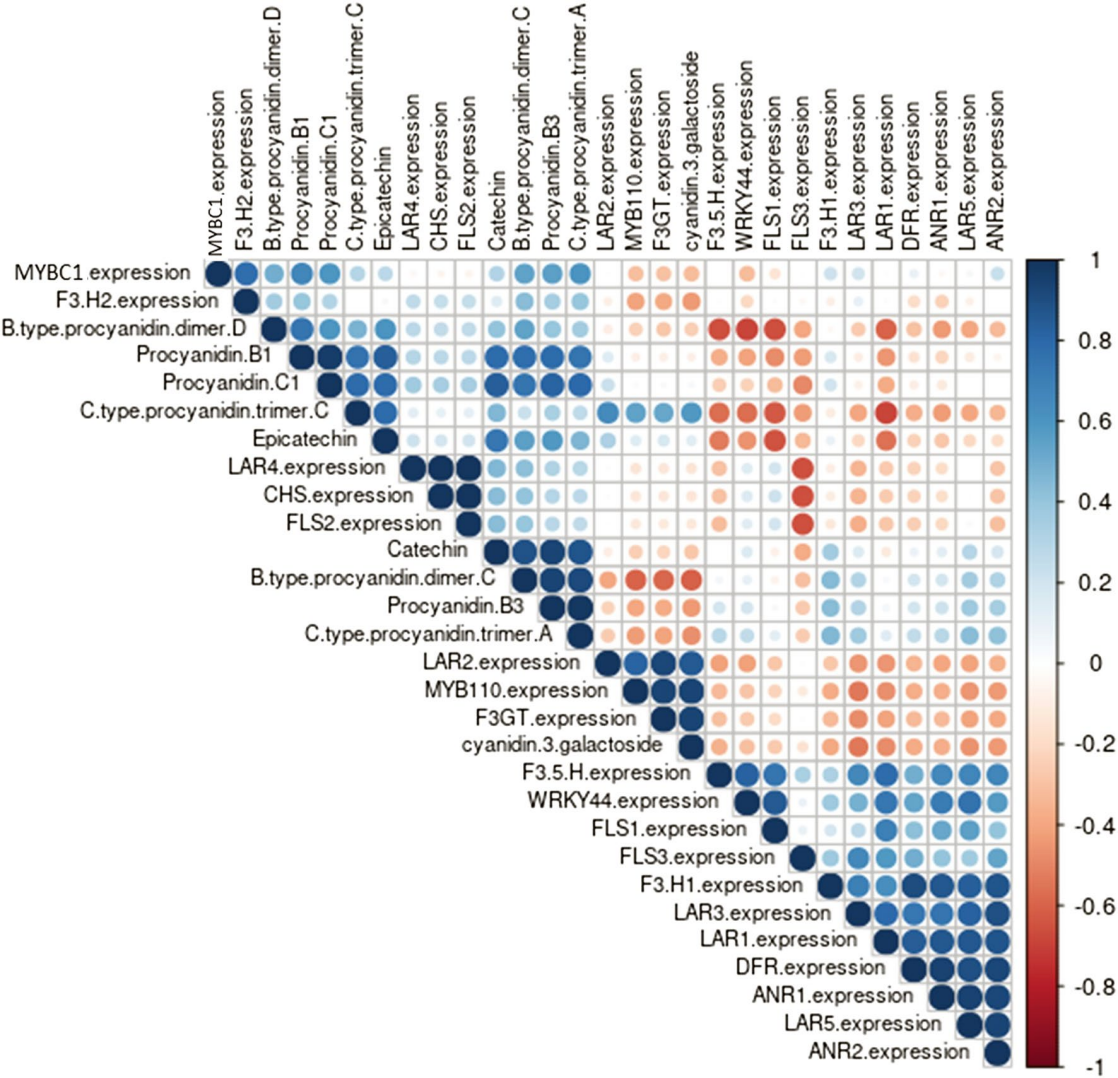
Correlation matrix of the relationship between gene expression and metabolite concentrations in the stably transformed *Actinidia arguta* calli. Dark and big blue dots indicate strong positive correlation while small red dots indicate weak negative correlation. Correlation with p value < 0.05 were shown, statistically non-significant correlation were left blank.

Pearson’s correlation analysis indicated strong positive correlations between *MYB110, F3GT, LAR2*, cyanidin-3-galactoside and C type procyanidin trimer C (Fig. 9, Supplementary Tables 7, 8). The expression of *MYBC1* was strongly correlated with the expression of *F3′H2* and the accumulation of B type procyanidin dimer C and D, procyanidin B1, B3, C1 and C type procyanidin trimer A. The expression of *WRKY44* was strongly correlated with the expression of *F3′5′H*, *F3′H1, FLS1*, *LAR1, LAR3, LAR5, DFR, ANR1* and *ANR2*.

## Discussion

### Kiwifruit *MYBC1* and *WRKY44* are up-regulated during colour development

Anthocyanin accumulation is regulated at the transcriptional levels by genes encoding biosynthetic enzymes and TFs. Previous studies on red and purple kiwifruit species revealed that the key pathway genes *CHS, DFR, F3GT* and *LDOX* are responsible for anthocyanin accumulation^19,50,51^. The R2R3 MYB TFs, MYB10 (AcMYB75/AcMYBF110) and MYB110, positively regulate the anthocyanin pathway at the transcriptional level^16,18,19,52^. However, the transcriptional regulation of the cyanidin and delphinidin branch points controlled by the *F3′H* and *F3′5′H* has not been fully understood. In this study, transcriptomic analysis of purple kiwifruit at three developmental stages revealed and confirmed genes involved in the anthocyanin biosynthetic pathway (Figs. 1, 2). Two TFs, *MYBC1* and *WRKY44,* exhibited similar expression patterns to the anthocyanin biosynthesis regulator, *MYB110* and coincided with anthocyanin accumulation.

### Kiwifruit *MYBC1* and *WRKY44* are PA-related TFs

Kiwifruit MYBC1 belongs to the subgroup 5 of the MYB TF family and shared amino acid residues found in the highly conserved motif VIRTKAx[K/R]C that is characteristic to the PA-regulating TFs, but the C1 and C3 motifs seen in the anthocyanin modulating VvMYB5 were not present in the c-terminus of kiwifruit MYBC1 (Fig. 4A, Supplementary Fig. 4)^31,34^. Generally, MYB TFs from subgroup 6 contribute to the regulation of the anthocyanin pathway while MYB TFs from subgroup 5 are thought to be involved in PA accumulation. In *Arabidopsis*, TT2 is responsible for PA accumulation in the endothelium during seed development by regulating flavonoid genes such as DFR and BAN (anthocyanidin reductase) as well as TT8 (bHLH) and TTG1 (WD40)^53^. In grape berries, VvMYB5a and VvMYB5b are capable of inducing anthocyanin and flavonol accumulation when over-expressed in tobacco^31,54^. *VvMYB5b* was also able to activate the promoters of structural genes involved in the anthocyanin and PA pathways such as *VvLAR1, VvANS, VvANR* and *VvF3′5′H*, suggesting a regulatory role in different branches of the phenylpropanoid pathway. Tea CsMYB5a and CsMYB5e and freesia FhMYB5 all demonstrated regulatory roles in the anthocyanin and PA biosynthesis pathway^33,34^. Recently, in red-centred kiwifruit *A. chinensis* ‘Hongyang’, a TT2 type R2R3 MYB TF, AcMYB123 and a bHLH TF, AcbHLH42 were identified to be involved in the inner pericarp-specific accumulation of anthocyanins by activating the expression of AcANS and AcF3GT^42^. Although belonging in the same subgroup 5 as the kiwifruit MYBC1 identified in this study, AcMYB123 (Acc28234.1, MH643775.1) only shared 47% sequence identity and the expression was barely detected in sequenced tissues. MYBC1 from *A. arguta* is highly similar to MYBC1 identified here (98% amino acid sequence identity) and is implicated to be involved in anthocyanin biosynthesis. It is negatively regulated by microRNA858^43^. Expression of other TT2-like MYBs was barely detected in the purple kiwifruit species studied here, making the possibility of a role in regulation unlikely (Supplementary Figs. 2, 4).

Kiwifruit WRKY44 (Acc16887.1) belongs to group I of the WRKY superfamily and clusters in the TTG2 clade closely with tea CsWRKY44, which is involved in the catechin regulation (Fig. 4B)^36^. Within the TTG2 clade, Arabidopsis *TTG2* encodes for a WRKY TF that participates in trichome formation and tannin production in seed coat endothelium by regulating the vacuolar transport step in PA pathway^35,55^. Moreover, kiwifruit WRKY44 is homologous to PH3 in petunia, which regulates vacuolar acidification for anthocyanin storage^39^. In petunia flower petals, *PH3* encodes for a WRKY TF that is highly similar to AtTTG2 and the transcription is activated by the MBW complex which then activates the transcription of a vacuolar ATPase to hyper-acidify the vacuole. A close phylogenetic relationship with these WRKY TFs indicates a possible regulatory role for kiwifruit WRKY44 in the anthocyanin and PA pathway.

### MYBC1 and WRKY44 regulate F3′H and F3′5′H branch points

Functional characterisation of MYBC1 and WRKY44 revealed that both TFs are able to induce anthocyanin accumulation when transiently over-expressed in tobacco leaves (Fig. 5). The regulatory roles of MYBC1 and WRKY44 were confirmed by the significant activation of all kiwifruit *F3′H* and *F3′5′H* promoters, for which the anthocyanin activator, *MYB110*, showed no regulatory role as previously found (Fig. 7)^19^. This observation was supported by the presence of multiple phenylpropanoid related MYB and WRKY TF binding sites identified on the promoter sequences (Fig. 6). These findings suggest that MYBC1 and WRKY44 transcriptionally regulate anthocyanin biosynthesis by activating the *F3′H* and *F3′5′H* branch points, which determines the hydroxylation patterns of the anthocyanin aglycone.

Kiwifruit calli over-expressing *MYBC1* and *WRKY44* had no obvious visual phenotype but metabolite and gene expression analysis revealed major differences from calli expressing a GUS-control (Fig. 8). The expression of kiwifruit *F3′5′H* was significantly up-regulated by the over-expression of *WRKY44* and *MYBC1*. Expression of genes encoding for biosynthetic enzymes in the PA pathway such as *FLS1, LAR1, LAR5, ANR1* and *ANR2* were elevated in the *WRKY44* and *MYBC1* calli compared to both *GUS-*control and *MYB110* calli. As a result, the amounts of PA accumulated in those calli increased significantly. Correlation analysis suggested linkage between *F3′H* and *F3′5′H* and the B- and C-type procyanidin isomers as a result of the over-expression of *WRKY44* and *MYBC1*. As expected, expression of *F3GT* was significantly elevated by the over-expression of *MYB110* in the anthocyanin accumulating calli, reiterating its critical role in anthocyanin regulation^50^. Anthocyanin accumulated in the calli over-expressing *MYBC1* and *MYBC1/WRKY44* at low levels, but with weak correlation data. However, the roots of the plantlets regenerated from the *MYBC1* and *MYBC1*/*WRKY44* calli were visibly red, differing from the unpigmented roots of *GUS* plantlets (Supplementary Fig. 7). Stable lines expressing these genes, as adult vines, will be important research tools.

### MYB110, MYBC1 and WRKY44 are in the regulatory network shared between PA and anthocyanin biosynthesis

Core genes encoding enzymes in the anthocyanin pathway, such as *CHS, DFR, F3GT,* and *LDOX,* have been shown to be transcriptionally regulated by the subgroup 6 R2R3 MYB TFs, MYB10 and MYB110 in kiwifruit species^16–19^. However the F3′H and F3′5′H branch points that decide the production of cyanidin and delphinidin were not principally regulated by MYB10 or MYB110 in purple kiwifruit species^19^. In grape MYBA1 activates *F3′5′H* promoters whereas two closely related TFs, MYBA6 and MYBA7 from subgroup 6, showed no activation of the *F3′5′H* promoters^56^. However, VvMYB5a and VvMYB5b from subgroup 5 activated the *F3′5′H* promoter by 12 fold as well as other genes involved in the PA and anthocyanin pathways^31^. In transgenic purple tomatoes, the control of flavonoid biosynthesis showed a specialised regulatory mechanism where the over-expression of TFs Del/Ros1 activated a broader spectrum of the genes including the *F3′5′H* in the flavonoid pathway but the anthocyanin-related TFs LC/C1 did not^57,58^. These results suggest different specificities of TFs controlling anthocyanin biosynthesis.

**Figure 10:**
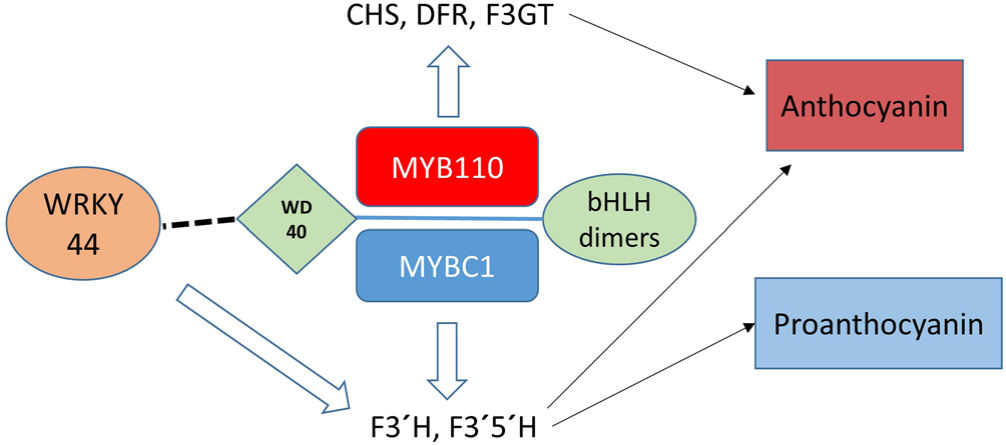
Model of anthocyanin and proanthocyanin pathway regulation by the MYB-BHLH-WD40-WRKY complex. Either the MYB110 or the MYBC1 is the driver of the complex, but not both at the same time. Big white arrows indicate the activation of the MBW-W complex on the biosynthetic genes. Black arrows indicate the impact of the biosynthetic genes on the pathways. Solid line indicates MBW complex partnership and dash line indicates possible interaction with the MBW complex by WRKY44.

Both MYBC1 and WRKY44 could regulate the anthocyanin pathway at the *F3′H* and *F3′5′H* branch points via the MBW complex in kiwifruit (Fig. 10). Functional characterisation showed that *MYB110*, *MYBC1* and *WRKY44* regulate different points of the pathway. We propose a model where, when MYB110 is incorporated into the MBW complex, the complex activates core genes such as *CHS, DFR*, and *F3GT* (Fig. 10). When MYBC1 forms a MBW complex with bHLH and WD40, it activates the *F3′H* and *F3′5′H* branch points which hydroxylate substrates to pass down the anthocyanin pathway. WRKY44 may activate the transcription by engaging with the MBW complex to form MBW-WRKY complex (Fig. 10). Petunia PH3 and Arabidopsis TTG2, homologous to kiwifruit WRKY44, were able to bind to the WD40 protein of the MBW complex and formed a MBW-W complex required to transcriptionally activate the target genes involved in vacuolar acidification (petunia) and hair development (Arabidopsis^39^). In grapevine, VvWRKY26, a homologue of petunia PH3 and AtTTG2, is recruited specifically by the VvMYB5a driven MBW complex to enhance the expression of target genes involved in vacuolar acidification, probably via the formation of a MBW-WRKY complex^40^. In kiwifruit, WRKY motifs are located in close proximity to the MYB and bHLH binding motifs on *F3′H* and *F3′5′H* promoters, suggesting the possible binding of the MBW-WRKY complex on the promoters for transcriptional activation (Fig. 6). Infiltration of WRKY44 (and MYBC1) was able to significantly increase the promoter activation.

The F3′H and F3′5′H enzymes are control points for the accumulation of cyanidin or delphinidin-based anthocyanins. However, they also hydroxylate immediate substrates that feed into the PA biosynthesis by the actions of the downstream enzymes (Supplementary Fig. 8). Activation of the *F3′H* and *F3′5′H* will increase the accumulation of dihydroquercetin and dihydromyricetin, which can be converted into anthocyanin precursors by DFR in the anthocyanin pathway or can be converted in quercetin and myricetin by FLS in the proanthocyanin pathway to form flavonols. The anthocyanin precursors can also be intercepted by LAR and ANR enzymes to generate PAs. While MYB110 may increase the general flux through the anthocyanin pathway by activating the core genes, MYBC1 and WRKY44 may increase intermediate substrates via regulation of *F3′H* and *F3′5′H* branch points and subsequent competition between enzymes. Hence, MYB110, MYBC1 and WRKY44 are proposed to participate in the same regulatory network shared by anthocyanin and proanthocyanin biosynthesis.

## Conclusion

The anthocyanin biosynthesis pathway and the associated transcriptional regulators have been well studied in a variety of plant species. In this study, the kiwifruit MYBC1 and WRKY44 transcriptionally regulate the key F3′H and F3′5′H branch points that have potential for controlling the types of flavonoids accumulated. The involvement of TFs shared by the anthocyanin and PA biosynthesis pathways adds to the potential to fine-tune the balance of the total metabolites in the fruit. These specific hydroxylation steps determine pigmentation as well as increasing nutritional values of fruit. Anthocyanin-enriched extracts from purple kiwifruit showed reduction of key inflammatory signals involved in lung inflammation^59^. Dietary intake of di- and tri-hydroxylated anthocyanins, flavonols, flavanols and PAs is linked to reductions in markers of cardiovascular disease risk^60,61^ as well as reducing the incidence of cardiovascular and metabolic diseases and cancers^62–64^. Understanding the transcriptional regulation of the metabolites will maximise the value of fruit crops as metabolites form a large basis of their nutritional benefits.

## Materials and methods

### Plant material

*Actinidia melanandra, A. purpurea* (sometimes referred to as *A. arguta* var. *purpurea*) and two progeny lines of a cross between *A. macrosperma* × *A. melanandra* (MaMe) were grown at the Plant and Food Research Orchard, Motueka, New Zealand as described previously^19^. Fruits were harvested at mature green stage and held at 20 °C for ripening. Skin peel and flesh tissues were separated and sampled into liquid nitrogen for mature green stage, colour change stage, and ripe stage.

### RNA-sequencing and transcriptomic analysis

RNA was isolated from the finely ground skin peel and flesh tissues of *A. purpurea,* MaMe Red and MaMe Yellow using the Spectrum Plant Total RNA kit (Sigma-Aldrich, USA) following manufacturer’s protocol. The RNA integrity number (RIN) of the extracted total RNA were above 7 and were sent to The Australian Genome Research Facility Ltd (AGRF) for Illumina RNA library constructions and Illumina HiSeq next generation sequencing. Quality of the RNASeq data was checked using FastQC (version 0.11.2) and using BBMap^65^ (version 37.93) adapter and quality trimming was carried out, where quality threshold of 20 and 10 bases from 5′ end were trimmed, with a minimum length of sequence after trimming set to 35 bases. Thereafter, quality trimmed reads were mapped to the manually annotated *A. chinensis* genome^44^ using STAR^66^ (version 2.5.2b) splice aware aligner with the ‘quantMode’ turned on. Resulting counts from STAR aligner were used in the differential expression (DE) analysis using DESeq (version 1.18.1) in R (version 3.4.3). The read counts were used to generate Reads per Kilobase Million (RPKM).

### Isolation, cloning and sequence alignment of candidate genes

The isolated RNA from *A. melanandra, A. purpurea,* MaMe Red and MaMe Yellow were reverse transcribed into cDNA using the QuantiTect Reverse Transcription kit (Qiagen, USA) following manufacturer’s protocols. Sequences of candidate genes were PCR amplified from cDNA of *A. melanandra, A. purpurea* and MaMe Red and cloned into over-expression vector pSAK277 using In-Fusion HD cloning (Takara Bio USA, Inc). Genes were sequenced by Macrogen, Korea. Multiple nucleotide and amino acid sequence alignments and phylogeny trees were created by Geneious 10.0.8.

### Transient over-expression of candidate genes in tobacco leaves

Over-expression vector pSAK277 with 35S promoter driving the expression of the candidate gene was transformed into *Agrobacterium tumefaciens* strain GV3101 by electroporation followed by incubation^19^. *Nicotiana tabacum* plants were grown under glasshouse conditions using natural daylight with extension to 16 h. Three leaves of the 6-week-old *N. tabacum* were infiltrated with *Agrobacterium* and kept under the same growth conditions. Leaves were photographed and harvested at 7 days after infiltration and stored at − 80 °C until analysis.

### Anthocyanin quantification by high performance liquid chromatography (HPLC)

Anthocyanin accumulation from transient over-expression of candidate genes in tobacco leaves was confirmed and measured by high performance liquid chromatography (HPLC–DAD). Approximately 300 mg of freeze-dried tissue powder were used to extract anthocyanin with acidified methanol (0.1% HCl) for two hours at room temperature. The supernatant was spin-dried and resuspended in 20% methanol followed by filtration by syringe filter and diluted with 20% methanol for analysis. Identification was achieved using an Acclaim PA2 C18 column (Dionex, ThermoFisher Scientific) maintained at 35 °C in a Dionex UltiMate 3000 Series HPLC with photodiode array detection at 520 nm (ThermoFisher Scientific, USA). 100% of solvent A (0.1% formic acid) was ramped to 98% at 5 min, then 85% A at 10 min, 80% A at 20 min and 100% B (acetonitrile + 0.1% formic acid) at 34 min at the flow rate of 350 µl/min for 40 min per 5 µl sample run. Standard for cyanidin-3-glucoside was used to quantitate anthocyanin concentrations which are reported as cyanidin-3-glucoside equivalents per gram of fresh weight (FW) or dry weight (DW).

### Dual luciferase transient assay in tobacco leaves

Genomic DNA was extracted from skin tissue of *A. melanandra, A. purpurea,* MaMe Red and MaMe Yellow using the DNeasy Plant Mini kit (Qiagen, USA). The 2.3 kb promoter regions of *F3′H* and *F3′5′H* were isolated by PCR and cloned into the vector pGreen 0800-LUC vector using In-Fusion HD cloning and transformed into *A. tumefaciens* GV3101, as mentioned previously^19,67^. The promoter sequences were confirmed by sequencing (Macrogen, Korea) and potential binding motifs were screened by the Plant Promoter Analysis Navigator PlantPAN3.0 https://plantpan.itps.ncku.edu.tw/^48^. The promoter activation dual luciferase assays were performed on leaves of 6-week-old *N. benthamiana* plants by co-infiltrating the vector carrying the promoter sequence with the over-expression vector containing candidate genes. Four days after infiltration, four leaf discs from each treatment were sampled to assay the firefly luciferase and renilla luciferase assay reagents (Targeting systems, USA). The promoter activities were expressed as a ratio of LUC to REN activity.

### Stable over-expression of candidate genes in *A. arguta*

Newly initiated leaves of *A. arguta* genotype K2D4 from in vitro grown shoots were excised and inoculated with *Agrobacterium tumefaciens* strain EHA105 containing the vectors pSAK277 with CaMV 35S:MYB110, 35S:MYBC1, 35S:WRKY44, and 35S:GUS, respectively. The transformation procedure was based on previous reports^68,69^. Calli formed in the selection medium containing 150 mg/l of kanamycin were excised individually and transferred to fresh regeneration medium containing 150 mg/l of kanamycin for calli growth and bud induction. Approximately 12 weeks after transformation, half of the calli were powdered in liquid nitrogen and stored in − 80 °C until analysis. The rest of the calli were subcultured in fresh regeneration medium. Shoots generated from these calli were excised and subcultured in medium for root elongation before being potted and grown in the containment glasshouse.

### Real time quantitative PCR expression analysis

RNA was isolated from the powdered calli using the Spectrum Plant Total RNA kit (Sigma-Aldrich, USA) and reverse transcribed into cDNA using the QuantiTect Reverse Transcription kit (Qiagen, USA) following manufacturer’s protocols. Genes of the anthocyanin and proanthocyanin pathways were identified by BLAST with genes of known function on the *Actinidia chinensis* genome and the *Actinidia arguta* RNA-seq data^44,70^. Gene-specific oligonucleotide primers were designed using Geneious 10.0.8 and are summarised in Supplementary Table 9. RT-qPCR was carried out using the LightCycler 480 instrument with LightCycler 480 SYBR Green I Mastermix (Roche Diagnostics, USA). Each reaction volume was 5 µL and reactions were run in quadruplicate, and non-template control and water control were included in each run. The thermal cycling conditions were 95 °C for 5 min, followed by 50 cycles of 95 °C for 10 s, 60 °C for 10 s and 72 °C for 20 s, then a melting temperature cycle with continuous fluorescence data acquisition from 65 to 95 °C. The data output was analysed by the LightCycler480 software Version 1.5 (https://lifescience.roche.com/en_nz/products/lightcycler14301-480-software-version-15.html) using the Target/Reference ratio to compare the expression level of the target genes normalised to the reference gene, elongation factor 1-α *EF1α*.

### Proanthocyanin and anthocyanin quantification in *A. arguta* calli

The proanthocyanin and anthocyanin content of the calli was determined by liquid chromatography-mass spectrometry (LC–MS) using an LTQ linear ion trap mass spectrometer fitted with an ESI interface (ThermoFisher Scientific, San Jose, CA, USA) coupled to an Ultimate 3000 UHPLC and PDA detector (Dionex, Sunnyvale, CA, USA) as described previously^71^. For proanthocyanins, to each sample (~ 40 mg fresh weight) was added 1 ml of ethanol/water 95:5 (v:v) and 0.8 g stainless steel beads 0.9–2 mm (Next Advance Inc., NY, USA). Samples were bead beaten for 4 min (Bullet Blender 24 Gold, Next Advance Inc., NY, USA) and were extracted overnight. After centrifugation at 13,000 × *g* for 5 min, the supernatant was evaporated to dryness under a stream of nitrogen at 35 °C and reconstituted in 10% methanol (100 µl) for analysis. Compound separation was achieved using a Hypersil GOLD aQ 1.9µ C18 175 Å (Thermo Scientific, Waltham, MA, USA), 150 × 2.1 mm column maintained at 35 °C. The solvents were (A) water + 0.1% formic acid and (B) acetonitrile + 0.1% formic acid (flow rate, 200 µl/min). The initial mobile phase, 95% A/5% B, was held for 5 min, then ramped linearly to 90% A at 10 min, 83% A at 25 min, 77% A at 30 min, 70% A at 40 min, 3% A at 48 min and held for 5 min before resetting to the original conditions. The sample injection volume was 4 μl. The MS data were acquired in the negative mode using a data dependent LC-MS^4^ method. This method isolates and fragments the most intense parent ion to give MS^2^ data (daughter ions), then isolates and fragments the most intense daughter ion (MS^3^ data), then granddaughter ion (MS^4^ data). The ESI voltage, capillary temperature, sheath gas pressure and sweep gas were set at − 10 V, 275 °C, 35 psi and 5 psi, respectively. Standards for catechin and epicatechin were used to quantitate proanthocyanin concentrations, which, with the exception of epicatechin which is reported as itself, are reported as catechin equivalents per mg of fresh weight (FW).

For anthocyanins, to each sample (~ 10 mg fresh weight) was added 1 ml of methanol/formic acid 95:5 (v:v) and 0.8 g stainless steel beads 0.9–2 mm (Next Advance Inc., NY, USA). Samples were bead beaten for 4 min (Bullet Blender 24 Gold, Next Advance Inc., NY, USA) and were extracted overnight. After centrifugation at 13,000 × *g* for 5 min, the supernatant was evaporated to dryness under a stream of nitrogen at 35 °C and reconstituted in acetonitrile/formic acid/water (5:3:92 v:v:v; 100 µl) for analysis. Compound separation, identification and quantitation by LC–MS were as described^19^.

### Statistical analysis

The statistical significance of the difference between the empty vector control and the treatment means in the dual luciferase transient assays was tested by two sample *t-*test. One-way ANOVA was used to determine the statistically significant differences in metabolite and gene expression analysis between the calli transformed with different candidate genes. Correlation matrix using Pearson’s correlation with significance level was calculated and produced in R Studio version 1.2.5033.

## Supplementary information

Supplementary Information.
