# Supplementary material for: The proanthocyanin-related transcription factors MYBC1 and WRKY44 regulate branch points in the kiwifruit anthocyanin pathway
Source: Sci Rep. 2020 Aug 25;10:14161. doi: 10.1038/s41598-020-70977-0 (PMC7447792; doi:10.1038/s41598-020-70977-0)
Supplement: Supplementary file 1 — Supplementary Information. [file 41598_2020_70977_MOESM1_ESM.pdf]

The proanthocyanin-related transcription factors MYBC1 and WRKY44 regulate branch points in the kiwifruit anthocyanin pathway

Yongyan Peng [1, 2], Amali H. Thrimawithana [2], Janine M. Cooney[3], Dwayne J. Jensen[3], Richard V. Espley [1, 2], Andrew C. Allan [1, 2]

[1] School of Biological Sciences, University of Auckland, 3 Symonds Street, Auckland, New Zealand

[2] The New Zealand Institute for Plant and Food Research Limited, 120 Mt Albert Road, Auckland, New Zealand

[3] The New Zealand Institute for Plant and Food Research Limited, Bisley Road, Ruakura, Hamilton 3214, New Zealand

Yongyan.peng@plantandfood.co.nz *correspondent author

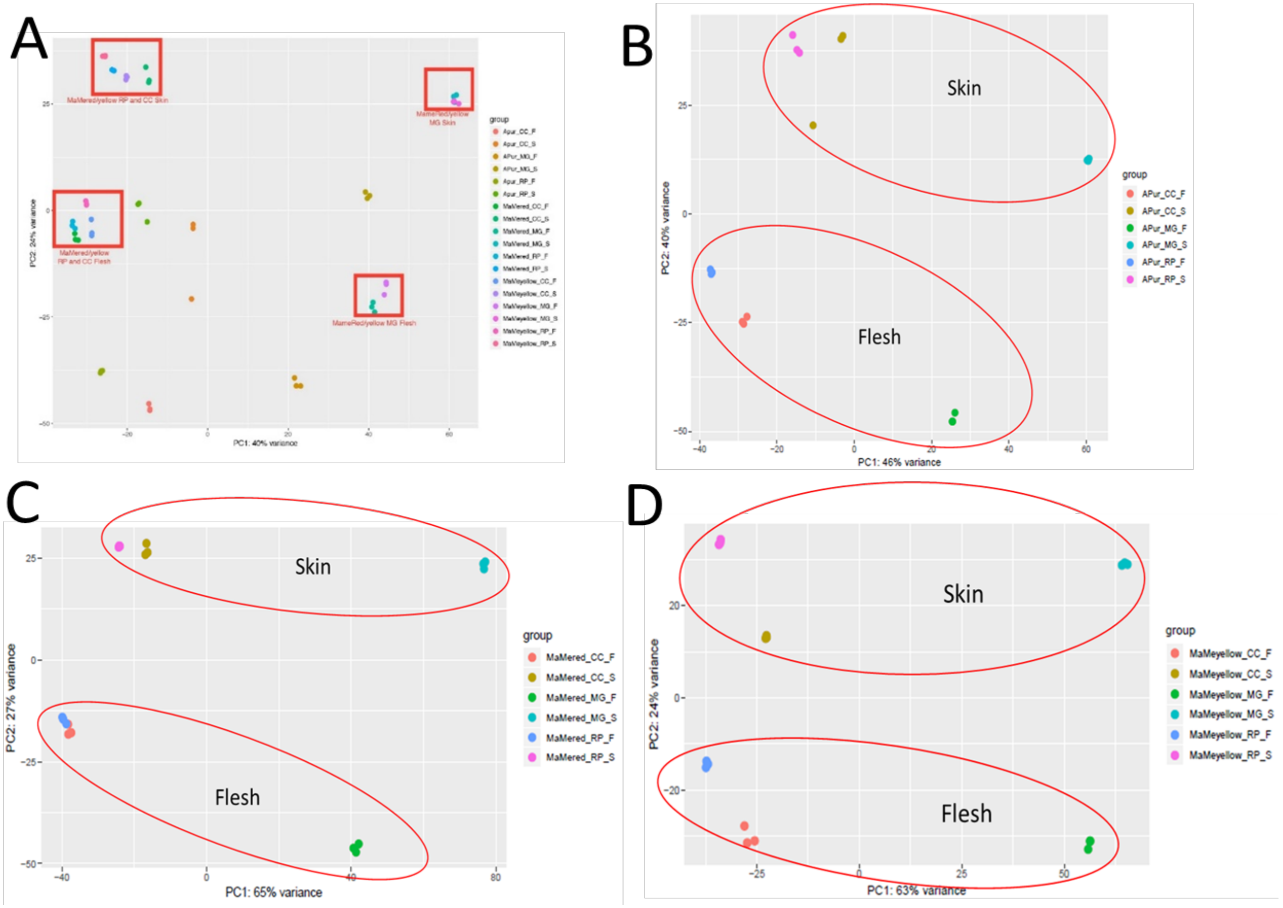

Supplementary Figure 1. Principal component analysis (PCA) plot of transcriptomic data of skin (S) and flesh (F) from mature green (MG) stage, colour change (CC) stage, and ripe (RP) stage. (A) Skin and flesh tissue from A. purpurea (Apur), MaMe Red (MaMered) and MaMe Yellow (MaMeyellow) across three developmental stages. (B) Skin and flesh tissue of A. purpurea (Apur) across the three developmental stages. (C) Skin and flesh tissue of MaMe red (MaMered) across the three developmental stages. (D) Skin and flesh tissue of MaMe Yellow (MaMeyellow) across the three developmental stages. Three biological replicates from each tissue type per developmental stages are presented in the same colour code.

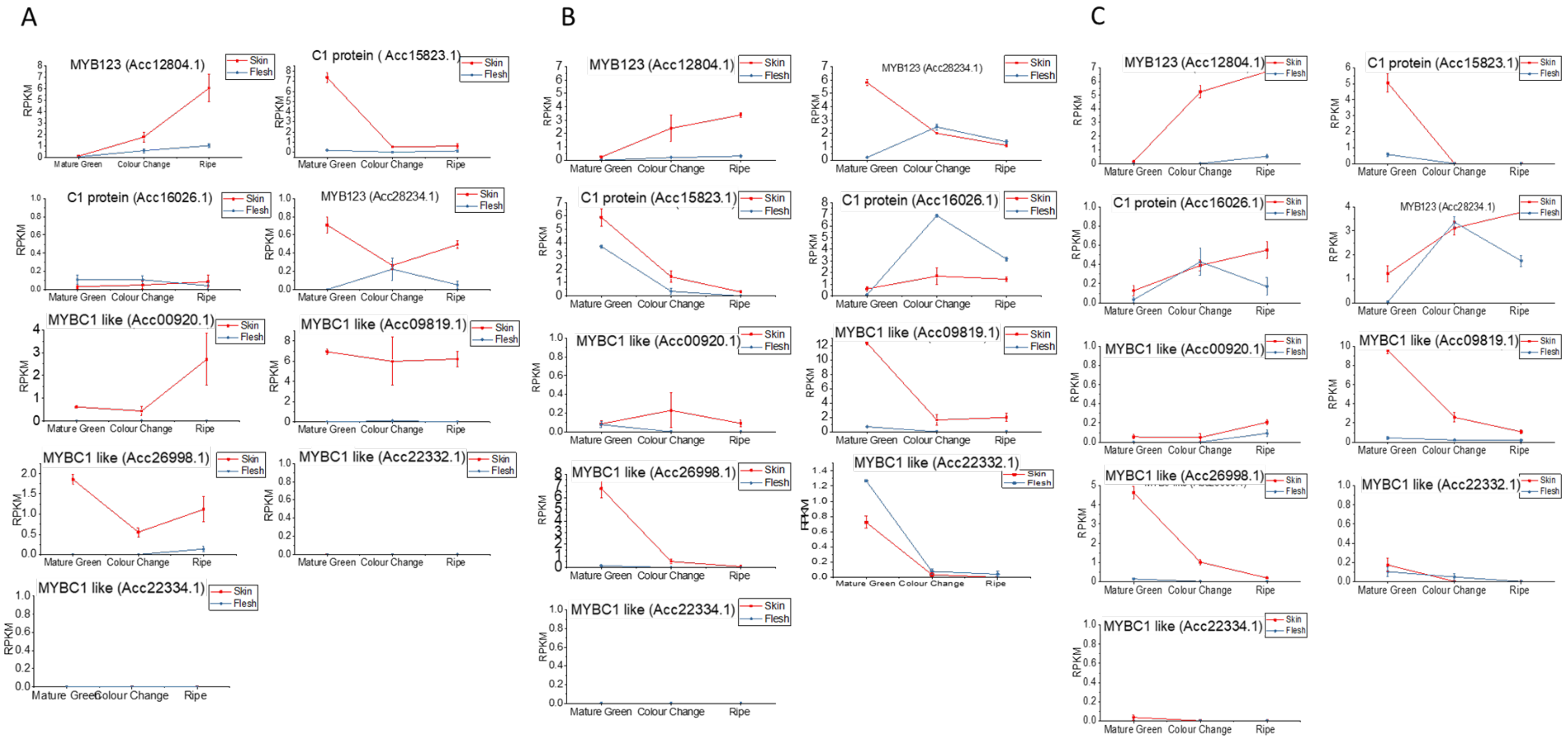

Supplementary Figure 2. The expression of other MYBC1-like gene models from the transcriptomic data of *A purpurea* (A), MaMe Red (B), and MaMe Yellow (C) at the three developmental stages. Gene expression was measured in Reads per Kilobase Millions (RPKM). Data were shown as means ± SEM of the three biological replicates.

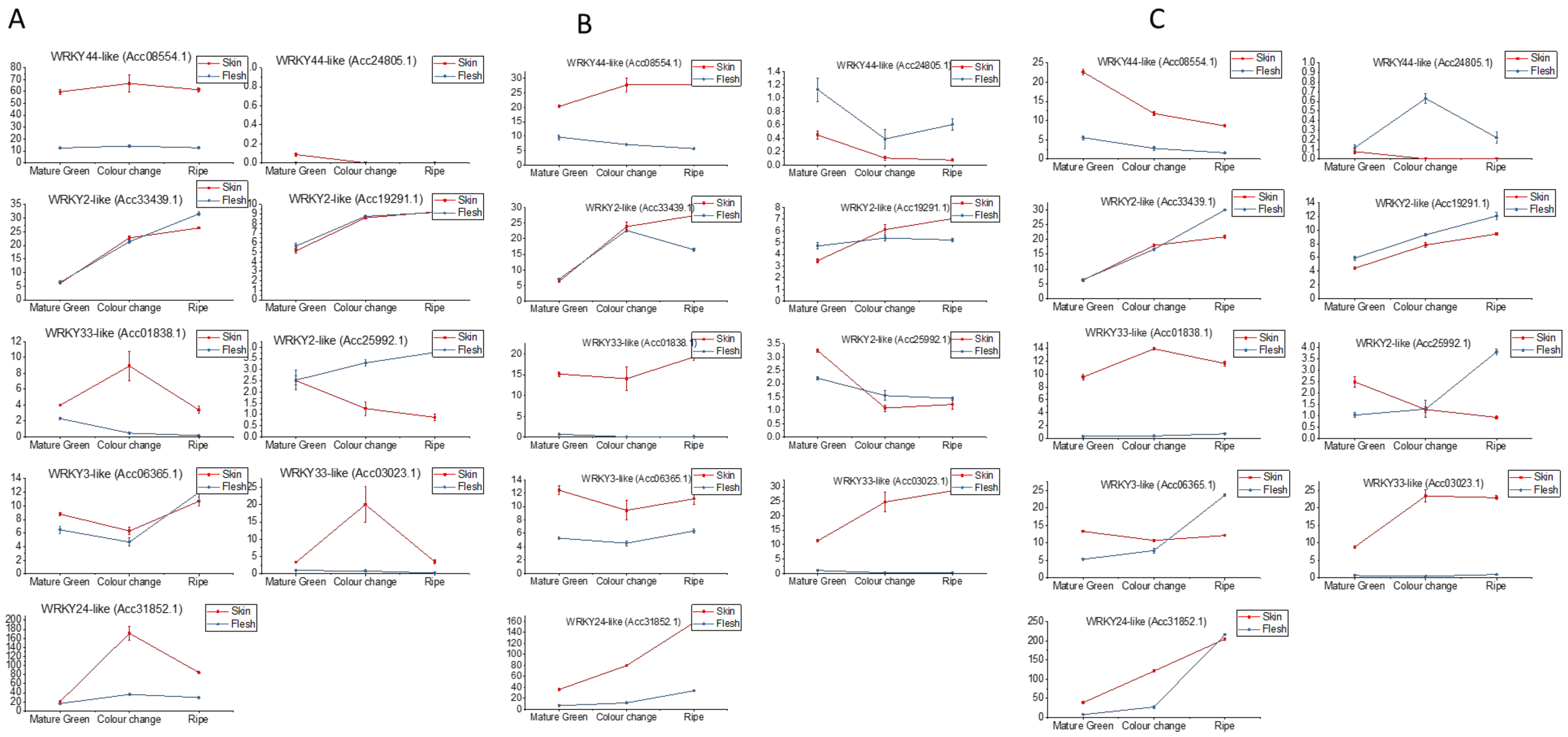

Supplementary Figure 3. The expression of other WRKY-like gene models from the transcriptomic data of A purpurea (A), MaMe Red (B), and MaMe Yellow (C) at three developmental stages. Gene expression was measured in Reads per Kilobase Millions (RPKM). Data were shown as means ± SEM of the three biological replicates.

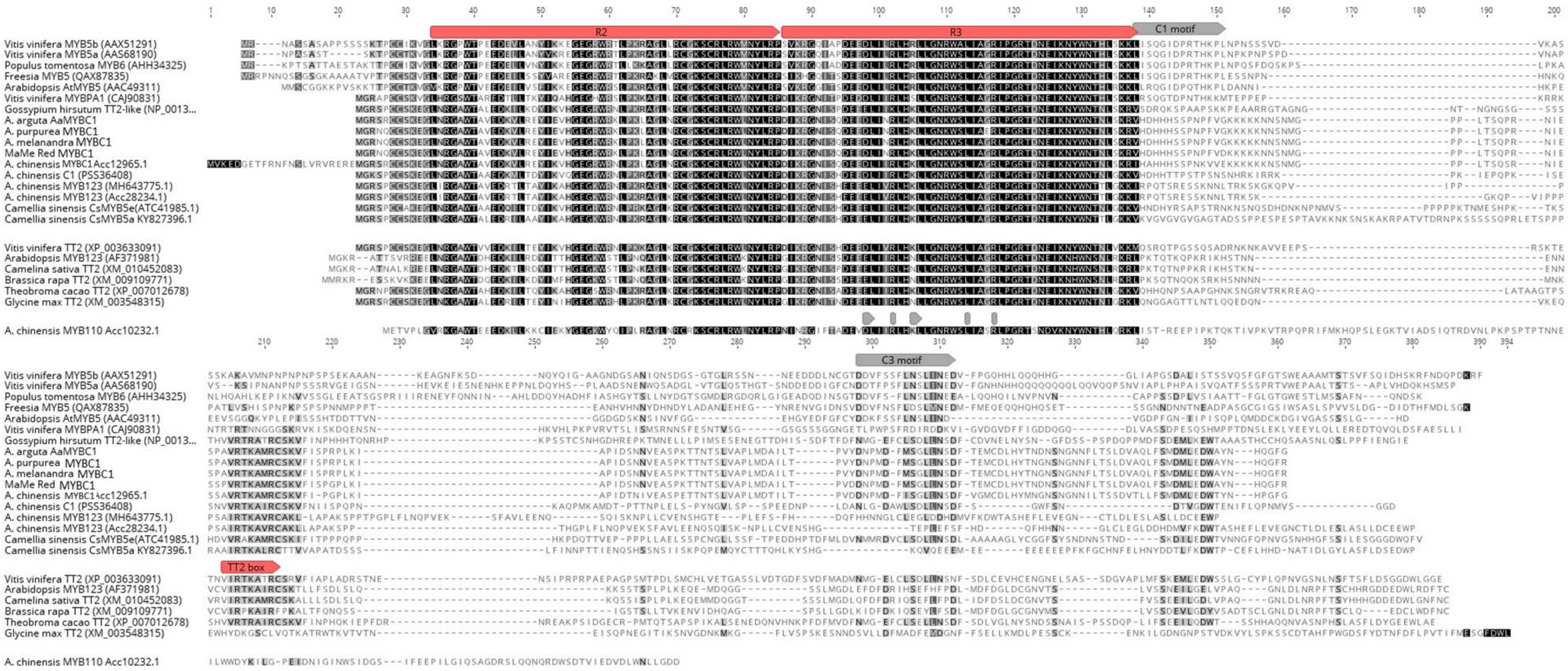

Supplementary Figure 4. Alignment of deduced amino acid of MYBC1 cloned from *A. melanandra, A. purpurea,* and MaMe Red with R2R3 MYB TFs from subgroup 5 from other plant species. The R2R3 domains are annotated as the red bar. C1 and C3 motifs are annotated as the grey bar. The TT2 box motif is highlighted in red box as described in Jiang et al., 2018.

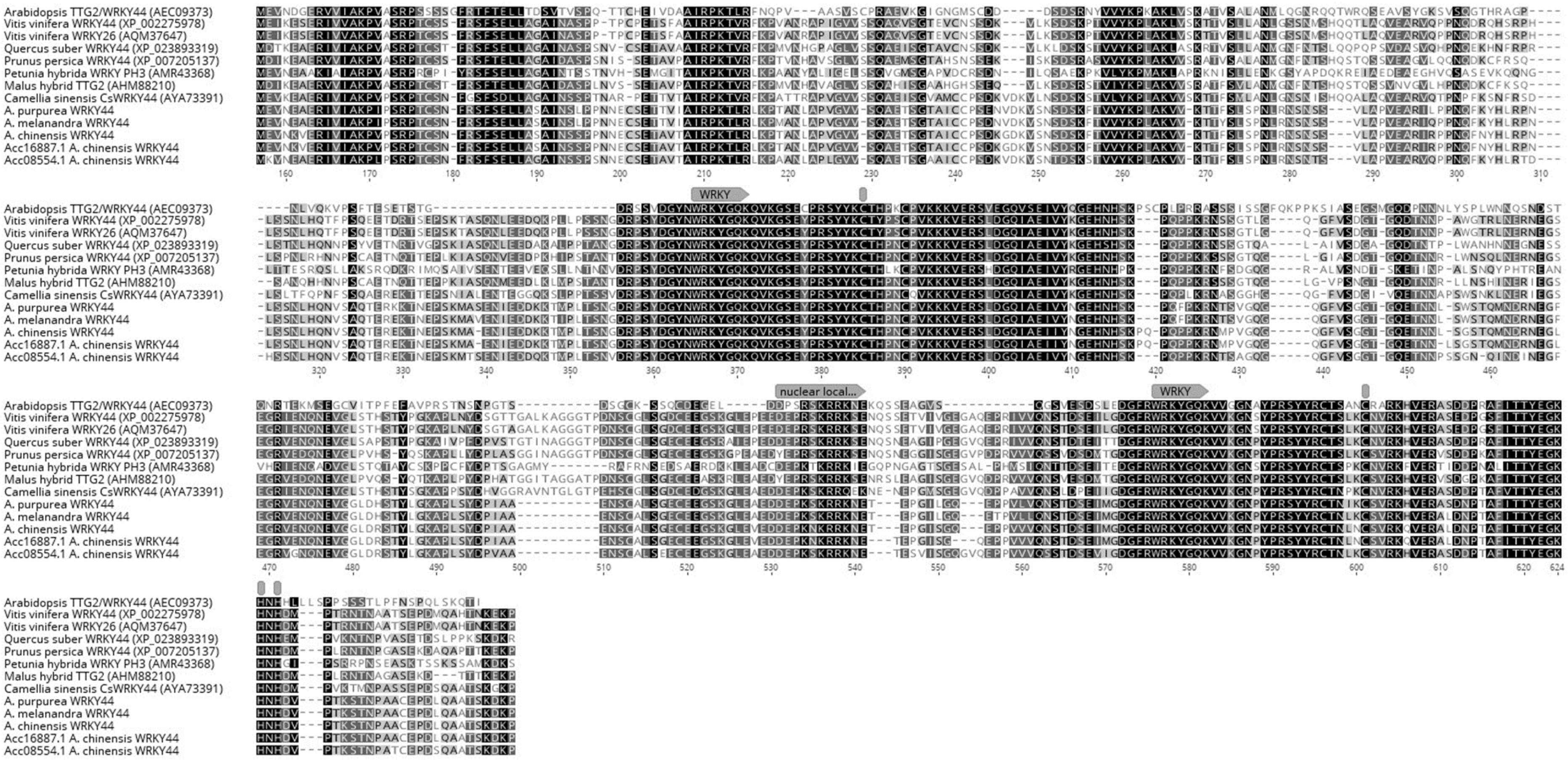

Supplementary Figure 5. Alignment of deduced amino acid of WRKY44 cloned from A. melanandra and A. purpurea with WRKY44 transcription factors from other plant species including A. chinensis. The two signature WRKY domains (WRKYGQK) and the potential nuclear localisation motif are annotated. The potential zinc finger like motif is also annotated by the grey boxes.

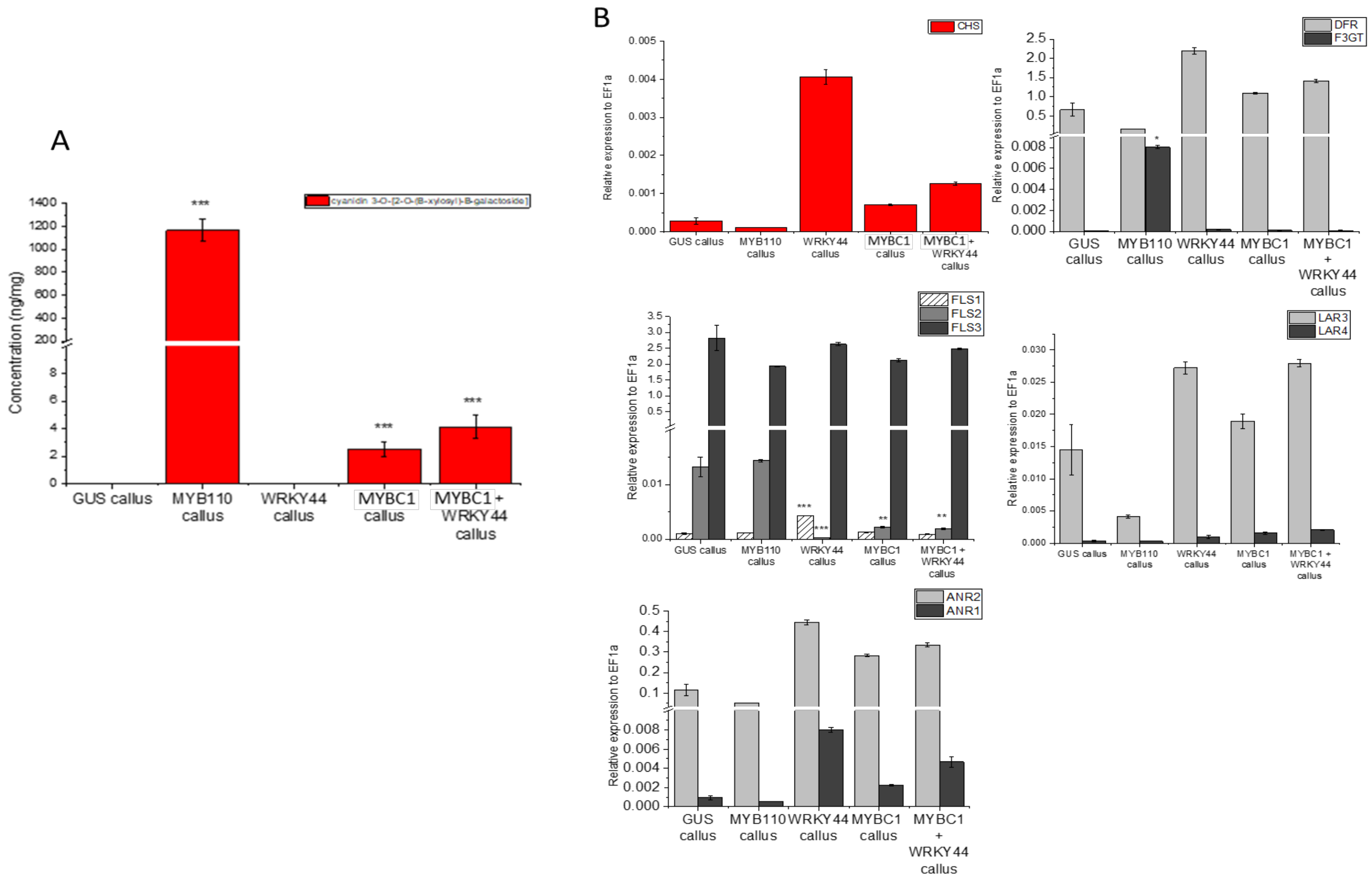

Supplementary Figure 6. Metabolite and gene expression analysis of transgenic A. arguta calli over-expressing GUS, MYB110, WRKY44, MYBC1 and MYBC1+WRKY44 co-expression. (A) Amount of cyanidin-based anthocyanin detected in the calli (B) Gene expression analysis of genes involved in proanthocyanin and anthocyanin pathways in the calli. Data were shown as means ± SEM of three biological replicates. $p<0.05$*, $p<0.01$**, and $p<0.001$***.

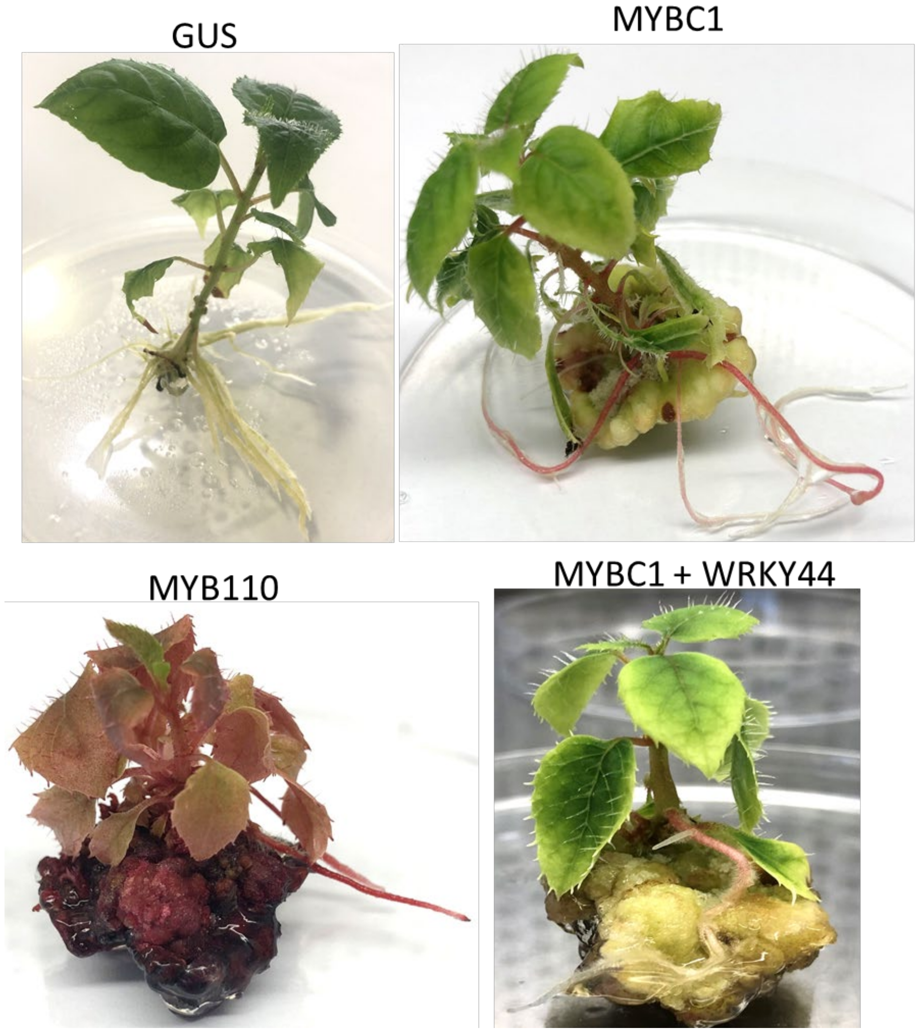

Supplementary Figure 7. Regeneration of plantlets from the stable transformed kiwifruit Actinidia arguta ‘K2D4’ over-expressing GUS (negative control), MYBC1, MYB110 (positive control), and MYBC1 co-infiltrated with WRKY44.

Supplementary Figure 8

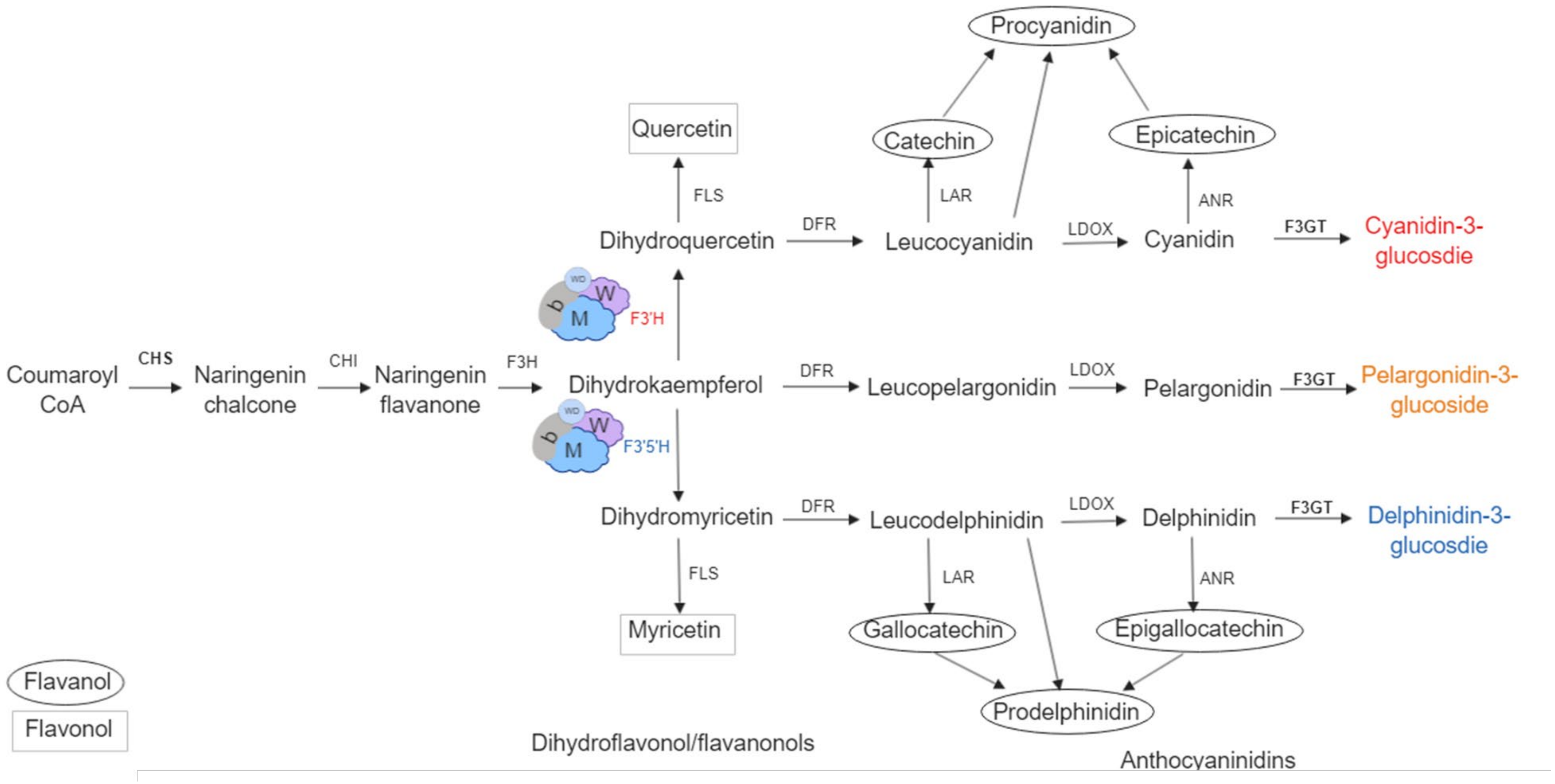

Supplementary Figure 8. The general flavonoid pathway showing the diversion of substrates into producing anthocyanin and proanthocyanin.

Supplementary Table 1. Number of pair-end reads mapped to the A. chinensis genome. Apur: A. purpurea, MaMered: MaMe Red, MaMeyellow: MaMe Yellow, MG: mature green stage, CC: colour change stage, RP: ripe stage, S1, S2, S3: skin biological replicates 1, 2, and 3, F1, F2, F3: flesh biological replicates 1, 2, and 3.

**Sample**	**Uniquely mapped percentage (%)**	**Uniquely mapped reads**
APur_MG_F1	80.5	3234967.5
APur_MG_F2	80.7	3261620
APur_MG_F3	80.2	2965417.5
APur_MG_S1	78.8	3687271.5
APur_MG_S2	78.9	3133372.25
APur_MG_S3	79.1	3156630
Apur_CC_F1	81.6	2829075.5
Apur_CC_F2	79.3	2587188
Apur_CC_F3	81.8	2810535.75
Apur_CC_S1	80.5	2590951
Apur_CC_S2	82.6	2900683
Apur_CC_S3	81.6	2778010.5
Apur_RP_F1	81.9	2773473.5
Apur_RP_F2	82.1	3357388.75
Apur_RP_F3	81.6	2753101.5
Apur_RP_S1	81.6	3251014.75
Apur_RP_S2	81.4	3109048.75
Apur_RP_S3	82.1	2967204.5
MaMered_CC_F1	82.0	3032523
MaMered_CC_F2	81.9	3120880
MaMered_CC_F3	82.0	2780843.25
MaMered_CC_S1	82.1	3243955.75
MaMered_CC_S2	81.5	2940111.5
MaMered_CC_S3	81.9	3038154.75
MaMered_MG_F1	77.1	2535944.75
MaMered_MG_F2	77.2	2614843
MaMered_MG_F3	77.2	2916275.5
MaMered_MG_S1	78.3	3013036.5
MaMered_MG_S2	78.2	2828394.25
MaMered_MG_S3	78.2	2706216
MaMered_RP_F1	80.9	2744339
MaMered_RP_F2	80.5	2656188.75
MaMered_RP_F3	81.0	3058618
MaMered_RP_S1	81.9	2726444
MaMered_RP_S2	82.0	2793044
MaMered_RP_S3	82.1	2844658
MaMeyellow_CC_F1	80.9	2545852.5
MaMeyellow_CC_F2	81.5	2504173
MaMeyellow_CC_F3	81.2	2697317.5

MaMeyellow_CC_S1	82.1	2702030.25
MaMeyellow_CC_S2	81.9	2763183.5
MaMeyellow_CC_S3	81.8	2643372.5
MaMeyellow_MG_F1	78.6	2924979.75
MaMeyellow_MG_F2	78.9	2684561.75
MaMeyellow_MG_F3	78.8	2581433
MaMeyellow_MG_S1	80.5	3139873
MaMeyellow_MG_S2	80.1	2703704.75
MaMeyellow_MG_S3	80.6	2735600.25
MaMeyellow_RP_F1	79.6	2633254
MaMeyellow_RP_F2	79.4	2691564.25
MaMeyellow_RP_F3	79.7	2596022.5
MaMeyellow_RP_S1	82.5	3058646.75
MaMeyellow_RP_S2	81.9	2926491.5
MaMeyellow_RP_S3	81.4	2698469.5

Supplementary Table 2. Differentially expressed genes (>2 Log2FC) identified from the comparison between MaMe Red and MaMe Yellow skin tissues from mature green (MG) stage, colour change (CC) stage, and ripe (RP) stage.

**Gene models**	**Probable description**	**MG**	**CC**	**RP**
Acc00260.1	Chalcone synthase 1	2.7	5.3	5.1
Acc00450.1	Malate dehydrogenase, cytoplasmic (putative)	2.8	3.3	3.4
Acc00549.1	Homeobox protein LUMINIDEPENDENS (similar to)	3.8	2.7	3.9
Acc00724.1	E3 ubiquitin-protein ligase DZIP3 (probable)	4.7	5.1	4.3
Acc00755.1	Zinc finger protein 2 (similar to)	6.0	6.7	6.7
Acc00804.1	Thioredoxin-like 3-1, chloroplastic, Precursor (probable)	-2.1	-2.3	-2.2
Acc01118.1	Monosaccharide-sensing protein 2 (putative)	-3.9	-4.5	-5.2
Acc01537.1	Laccase-5, Precursor (similar to)	2.5	-2.4	-2.9
Acc01600.1	hypothetical protein	-7.0	-5.9	-5.4
Acc01709.1	UPF0481 protein At3g47200 (probable)	5.4	11.3	11.8
Acc01710.1	Vegetative cell wall protein gp1, Precursor (probable)	4.2	9.8	10.3
Acc01711.1	Ankycorbin (probable)	4.7	9.6	9.6
Acc02004.1	Chalcone synthase 1	2.4	5.6	6.1
Acc02198.1	Interactor of constitutive active ROPs 2, chloroplastic, Precursor (putative)	-2.5	-5.0	-3.8
Acc02469.1	Protein WVD2-like 4 (probable)	-2.1	2.4	2.6
Acc02856.1	hypothetical protein	-9.0	-8.1	-7.8
Acc02973.1	Mitogen-activated protein kinase-binding protein 1 (JNKBP-1) (probable)	-5.6	-6.0	-7.8
Acc03257.1	Small heat shock protein, chloroplastic, Precursor (putative)	2.9	3.7	5.0
Acc03755.1	Glutathione S-transferase DHAR2 (CLIC homolog 2) (similar to)	-5.6	-3.1	-4.4
Acc04187.1	1-aminocyclopropane-1-carboxylate oxidase homolog 1 (putative)	2.1	4.3	7.1
Acc04256.1	Putative ribosomal-protein-alanine acetyltransferase (probable)	4.2	4.5	5.3
Acc04531.1	GDSL esterase/lipase EXL3 (Family II lipase EXL3), Precursor (similar to)	2.2	2.9	3.7
Acc04763.1	G2/mitotic-specific cyclin C13-1 (putative)	-4.5	-4.7	-4.8
Acc05003.1	Probable mannose-1-phosphate guanylyltransferase 2	6.6	6.1	4.2
Acc05179.1	7-deoxyloganetin glucosyltransferase (CrUGT6) (putative)	2.5	2.2	2.1
Acc05532.1	Citrate-binding protein, Precursor (probable)	-3.0	-3.4	-4.1
Acc05727.1	Hypersensitive-induced response protein 1 (AtHIR1) (similar to)	-2.3	-4.1	-3.6
Acc05734.1	Homeobox-leucine zipper protein HAT7 (HD-ZIP protein 7) (similar to)	4.4	5.6	4.5
Acc05794.1	2-hydroxy-6-oxononadienedioate/2-hydroxy-6-oxononatrienedioate hydrolase (probable)	-2.9	-3.7	-4.3
Acc05994.1	Lysine-rich arabinogalactan protein 17 (Lys-rich AGP 17), Precursor (probable)	2.0	-2.1	-3.4
Acc06294.1	Glutathione S-transferase U17 (AtGSTU17) (putative)	2.2	3.0	2.7

Acc06303.1	Homeobox-leucine zipper protein HOX16 (probable)	-2.0	-3.7	-3.6
Acc06838.1	Gamma-glutamyl peptidase 5 (probable)	2.9	2.8	2.2
Acc07495.1	Metacaspase-1 (AtMC1) (probable)	-7.7	-6.8	-5.4
Acc07557.1	Calcium-binding protein KIC (similar to)	3.4	2.4	2.9
Acc07655.1	Protein transport Sec1a (AtSec1a) (putative)	-2.0	-3.3	-2.6
Acc07922.1	Condensin complex subunit 3 (hCAP-G) (probable)	-11.3	-9.6	-7.7
Acc08329.1	Protein IQ-DOMAIN 14 (probable)	7.5	6.3	5.4
Acc08684.1	Probable cinnamyl alcohol dehydrogenase 6 (OsCAD6) (putative)	-4.8	-5.2	-5.2
Acc08970.1	Chalcone synthase 1	2.9	3.6	3.1
Acc08998.1	Type III polyketide synthase A (PKS-A) (putative)	2.9	4.7	3.8
Acc09117.1	Protein SDA1 homolog (probable)	-3.0	2.5	2.7
Acc09258.1	Annexin D3 (putative)	-3.2	-4.3	-4.8
Acc09580.1	(E,E)-geranyllinalool synthase (AtTPS04) (probable)	2.0	7.6	6.5
Acc09624.1	CRIB domain-containing protein RIC10 (probable)	-3.6	-4.6	-3.8
Acc10105.1	Dual specificity protein kinase shkD (probable)	5.7	5.8	4.5
Acc10176.1	Myb family transcription factor PHL5 (similar to)	-2.8	-2.8	-2.8
Acc10232.1	Transcription factor MYB114 (AtMYB114) (similar to)	3.0	9.1	10.3
Acc10233.1	Organellar oligopeptidase A, chloroplastic/mitochondrial, Precursor (similar to)	2.0	5.1	4.8
Acc10234.1	Organellar oligopeptidase A, chloroplastic/mitochondrial, Precursor (similar to)	2.2	7.4	5.5
Acc10275.1	Formin-like protein 6 (AtFH6), Precursor (similar to)	3.1	4.6	4.8
Acc10367.1	Isoeugenol synthase 1 (putative)	3.4	3.6	2.5
Acc10926.1	Pectin acetylesterase 12, Precursor (putative)	2.7	-2.6	-2.4
Acc10997.1	Disease resistance protein RPP13 (probable)	-2.1	-3.2	-2.3
Acc11082.1	Desiccation-related protein PCC13-62, Precursor [Craterostigma plantagineum] (similar to)	-2.4	-4.7	-3.1
Acc11191.1	Non-symbiotic hemoglobin 1 (similar to)	2.4	-3.2	-4.7
Acc11414.1	Probable sucrose-phosphate synthase 4 (AtSPS4F) (putative)	3.2	4.4	4.3
Acc11502.1	Snakin-1, Precursor (putative)	4.1	5.5	5.2
Acc11770.1	Protein csh3 (similar to)	2.2	5.8	4.5
Acc11774.1	Actinidain (Actinidin), Precursor (putative)	5.1	3.6	3.1
Acc11996.1	Cullin-2 (CUL-2) (probable)	-7.2	-5.2	-4.7
Acc12584.1	Probable 2-oxoglutarate-dependent dioxygenase AOP1.2	3.5	-3.2	-2.6
Acc12612.1	ATP-dependent DNA helicase DDX11 (probable)	-5.5	-4.9	-4.2
Acc12738.1	Inositol oxygenase 1 (AtMIOX1) (putative)	2.4	5.0	7.1
Acc12897.1	Calcium sensing receptor, chloroplastic, Precursor (probable)	-2.8	-4.3	-6.5
Acc12965.1	Anthocyanin regulatory C1 protein (similar to)	2.8	6.9	6.2
Acc13312.1	Protein RESTRICTED TEV MOVEMENT 2 (AtAcd41.3) (probable)	4.9	5.2	5.3
Acc13526.1	G-type lectin S-receptor-like serine/threonine-protein kinase SD1-1 (SD1-1), Precursor (similar to)	-3.3	-3.4	-4.2
Acc13672.1	Early nodulin-like protein 2, Precursor (similar to)	4.5	5.6	6.3

Acc13715.1	Cyclic nucleotide-gated ion channel 4 (AtCNGC4) (similar to)	-4.9	-7.5	-6.0
Acc13718.1	Nucleoporin NUP159 (probable)	5.9	2.3	5.4
Acc13851.1	Early nodulin-like protein 1 (OsENODL1), Precursor (similar to)	2.2	-2.8	-3.0
Acc13879.1	Anthocyanidin 3-O-glucoside 2''-O-glucosyltransferase (In3GGT) (putative)	2.9	2.6	3.9
Acc14115.1	Peroxidase 11 (Atperox P11), Precursor (putative)	4.2	5.6	7.4
Acc14951.1	Glucan endo-1,3-beta-glucosidase 12 ((1->3)-beta-glucanase 12), Precursor (probable)	-2.1	7.2	8.3
Acc14983.1	Condensin complex subunit 2 (AtCAP-H) (putative)	-3.5	-4.4	-4.2
Acc15495.1	Heavy metal-associated isoprenylated plant protein 30 (AtHIP30), Precursor (similar to)	-4.8	-2.9	-2.4
Acc16298.1	Protein MEI2-like 2 (OML2) (similar to)	4.4	2.7	3.7
Acc16630.1	Imidazole glycerol phosphate synthase subunit HisF (IGPS subunit HisF) (probable)	-4.0	-4.6	-5.1
Acc16712.1	Probable serine/threonine-protein kinase cdc7	5.0	4.7	2.9
Acc16762.1	Leucoanthocyanidin dioxygenase (LDOX) (putative)	3.0	7.4	9.7
Acc16814.1	Auxin transporter-like protein 2 (putative)	-2.8	-3.2	-2.8
Acc16949.1	Peptidyl-prolyl cis-trans isomerase CYP18-2 (PPIase CYP18-2) (similar to)	2.2	2.2	2.6
Acc17759.1	Zinc phosphodiesterase ELAC protein 2 (RNase Z 2), Precursor (probable)	-2.0	-5.1	-5.1
Acc17819.1	DNA replication complex GINS protein PSF1 (probable)	3.0	4.7	5.2
Acc17837.1	Probable 2-oxoglutarate-dependent dioxygenase AOP1.2	3.6	-3.8	-3.2
Acc17888.1	Glutamyl-tRNA(Gln) amidotransferase subunit D (Glu-ADT subunit D) (probable)	-2.3	-4.7	-3.1
Acc18031.1	Cytochrome P450 94B3 (similar to)	-3.1	-3.5	-2.2
Acc18426.1	RNA polymerase II subunit A C-terminal domain phosphatase SSU72 (CTD phosphatase SSU72) (probable)	-2.3	-2.7	-3.6
Acc18572.1	Protein PAT1 homolog 1 (Pat1b) (probable)	4.8	3.1	2.7
Acc19415.1	Cytochrome P450 716B1 (probable)	-6.7	-6.2	-6.3
Acc19451.1	Nucleoside diphosphate kinase (NDK) (probable)	5.2	2.9	5.9
Acc19566.1	hypothetical protein	-2.9	-4.6	-4.2
Acc19848.1	Putative UDP-rhamnose:rhamnosyltransferase 1 (FaRT1) (probable)	3.1	5.1	6.1
Acc20257.1	Anthocyanidin 5,3-O-glucosyltransferase (putative)	8.9	3.2	5.2
Acc20661.1	Protein E6 (similar to)	2.6	-2.0	-3.7
Acc21103.1	Scopoletin glucosyltransferase (putative)	-2.7	-4.6	-5.0
Acc21202.1	Sulfate transporter 1.3 (putative)	3.8	3.4	3.0
Acc21239.1	Basic blue protein, Precursor (similar to)	7.6	5.2	3.9
Acc21864.1	Kinesin-like protein KIN-8A (putative)	3.5	4.3	4.6
Acc21917.1	Transcription factor UNE10 (AtbHLH16) (probable)	5.5	5.6	4.4
Acc22572.1	Lachrymatory-factor synthase, Precursor (probable)	4.0	3.4	4.3
Acc22872.1	Lipid-phosphate phosphatase (probable)	-2.9	-5.4	-5.5
Acc23255.1	Rac-like GTP-binding protein ARAC7 (putative)	-7.0	-7.5	-6.3

Acc23285.1	Protein IQ-DOMAIN 31 (probable)	3.3	2.6	2.4
Acc23336.1	hypothetical protein	-2.4	-3.4	-3.2
Acc23438.1	Diacylglycerol kinase theta (DAG kinase theta) (probable)	-4.0	-3.8	-3.9
Acc23748.1	hypothetical protein	-4.1	-3.1	-5.8
Acc24129.1	7-deoxyloganetin glucosyltransferase (CrUGT6) (putative)	3.3	7.1	4.0
Acc24301.1	Phytochrome A1 (putative)	5.2	8.4	9.7
Acc24307.1	Transcription factor MYB86 (AtMyb4) (similar to)	-4.2	-2.9	-2.9
Acc24569.1	Major allergen Pru ar 1 (putative)	3.6	5.0	2.2
Acc24799.1	Probable LRR receptor-like serine/threonine-protein kinase At1g67720, Precursor	-3.4	-5.1	-4.1
Acc25392.1	Histone H2B (similar to)	-5.9	-4.6	-4.0
Acc25418.1	Vacuolar amino acid transporter 1 (probable)	-2.4	-3.1	-3.3
Acc25475.1	Transcription initiation factor TFIID subunit 13 (AtTAF13) (putative)	4.7	3.1	4.1
Acc25555.1	Protein PALE CRESS, chloroplastic, Precursor (similar to)	-3.0	-2.7	-2.5
Acc26480.1	Probable amino acid permease 7 (similar to)	4.1	6.9	5.6
Acc26719.1	Microtubule-associated protein TORTIFOLIA1 (probable)	-4.6	-2.6	-2.3
Acc26789.1	Putative multidrug resistance protein (similar to)	-4.8	-4.0	-4.6
Acc27189.1	Lipid-phosphate phosphatase (probable)	2.1	2.4	3.7
Acc27268.1	Pyruvate, phosphate dikinase, chloroplastic, Precursor (putative)	-4.6	-3.4	-3.0
Acc27430.1	Acetolactate synthase small subunit 1, chloroplastic (AHAS), Precursor (similar to)	3.8	-3.3	-3.9
Acc27478.1	Serine/threonine-protein phosphatase PP1 (putative)	2.8	2.1	3.4
Acc27537.1	Fanconi anemia group D2 protein homolog (Protein FACD2) (probable)	-2.2	-2.4	-2.6
Acc27584.1	Cyclin-dependent protein kinase inhibitor SMR3 (similar to)	-4.1	-4.7	-4.3
Acc27768.1	7-deoxyloganetic acid glucosyltransferase (CrUGT8) (putative)	-2.3	-3.4	-2.2
Acc27963.1	21 kDa protein, Precursor (probable)	-2.7	-2.4	-2.3
Acc27996.1	Probable fructokinase-7 (similar to)	3.2	4.8	3.9
Acc28072.1	Kinesin-like protein KIN-10C (probable)	6.7	5.1	4.2
Acc28618.1	Octanoyltransferase (probable)	-2.3	-3.4	-2.3
Acc28876.1	Leucoanthocyanidin dioxygenase (LDOX) (similar to)	3.2	8.2	11.3
Acc29052.1	Isoflavone reductase homolog (putative)	4.4	10.2	12.0
Acc29342.1	Protein SHOOT GRAVITROPISM 5 (AtIDD15) (similar to)	-6.3	-3.6	-2.8
Acc29524.1	Serine/threonine-protein kinase Aurora-1 (AtAur1) (putative)	2.4	4.4	3.2
Acc29604.1	UPF0317 protein Avi_5849 (probable)	-5.5	-5.0	-5.1
Acc29638.1	Glutamate-1-semialdehyde 2,1-aminomutase, chloroplastic (GSA), Precursor (putative)	3.7	5.0	5.0
Acc30051.1	Probable calcium-binding protein CML48 (putative)	2.8	-2.9	-3.1
Acc30521.1	Peroxidase 51 (Atperox P51), Precursor (putative)	-4.0	-4.6	-4.4
Acc30661.1	Protein NRT1/ PTR FAMILY 5.8 (AtNPF5.8) (putative)	-6.2	-4.7	-4.2

Acc31033.1	Geraniol 8-hydroxylase (SmG10H) (putative)	2.3	3.7	4.8
Acc31165.1	21 kDa protein, Precursor (probable)	-3.2	-5.5	-5.3
Acc31401.1	Myb-related protein B (B-Myb) (probable)	-3.0	-3.2	-2.3
Acc31616.1	Dephospho-CoA kinase (putative)	-2.2	-2.3	-3.2
Acc31674.1	Potassium transporter 5 (AtHAK1) (probable)	3.0	3.9	3.8
Acc31944.1	Receptor-like kinase TMK4, Precursor (putative)	-2.3	-6.9	-5.6
Acc32549.1	Subtilisin-like protease SBT3.18 (AtSBT3.18), Precursor (putative)	2.9	4.8	5.5
Acc32836.1	Nucleobase-ascorbate transporter 7 (AtNAT7) (putative)	6.5	6.4	8.9
Acc33413.1	Probable LRR receptor-like serine/threonine-protein kinase At4g26540, Precursor (putative)	-5.3	-7.8	-6.9
Acc33624.1	Acetyl-CoA-benzylalcohol acetyltransferase (CbBEAT-1) (probable)	2.1	3.0	2.4
Acc33633.1	Vegetative cell wall protein gp1, Precursor (probable)	4.2	9.6	10.1
Acc33695.1	Diacylglycerol kinase theta (DAG kinase theta) (probable)	5.8	10.3	10.8

Supplementary Table 3. Differentially expressed genes (>2 LogFC) identified from the comparison between MaMe Red and MaMe Yellow flesh tissues from mature green (MG) stage, colour change (CC) stage, and ripe (RP) stage.

Gene models	Probable description	MG	CC	RP
Acc00105.1	Kinesin-like protein KIN-10A (AtPAKRP2) (similar to)	-6.2	-4.8	-5.3
Acc00260.1	Chalcone synthase 1	4.0	6.3	4.1
Acc00549.1	Homeobox protein LUMINIDEPENDENS (similar to)	4.2	2.6	2.4
Acc00724.1	E3 ubiquitin-protein ligase DZIP3 (probable)	4.4	6.4	5.7
Acc00804.1	Thioredoxin-like 3-1, chloroplastic, Precursor (probable)	-3.7	-3.6	-2.8
Acc00853.1	Lon protease (probable)	3.3	7.0	6.0
Acc00999.1	Late embryogenesis abundant protein D-29 (LEA D-29) (probable)	2.0	3.6	3.8
Acc01600.1	hypothetical protein	-6.3	-5.6	-5.2
Acc01709.1	UPF0481 protein At3g47200 (probable)	8.5	10.3	9.3
Acc01741.1	BAHD acyltransferase DCR (probable)	4.0	3.4	2.1
Acc02004.1	Chalcone synthase 1	3.7	5.1	5.2
Acc02198.1	Interactor of constitutive active ROPs 2, chloroplastic, Precursor (putative)	-2.7	-5.0	-5.1
Acc02432.1	Phosphoenolpyruvate/phosphate translocator 2, chloroplastic (AtPPT2), Precursor (similar to)	2.0	3.5	2.7
Acc02557.1	4-coumarate--CoA ligase 2 (4CL 2) (putative)	2.3	3.7	3.3
Acc02856.1	hypothetical protein	-10.2	-8.1	-7.9
Acc03929.1	Glucan endo-1,3-beta-glucosidase, basic isoform ((1->3)-beta-glucanase), Precursor (putative)	2.2	-2.8	-2.6
Acc04014.1	Protein trichome birefringence-like 19 (putative)	-4.3	-7.8	-8.1
Acc04187.1	1-aminocyclopropane-1-carboxylate oxidase homolog 1 (putative)	2.2	4.5	5.3
Acc04224.1	3-ketoacyl-CoA synthase 11 (KCS-11) (putative)	-2.6	4.2	3.4
Acc04557.1	Transcription factor E2FC (AtE2F2) (similar to)	2.2	3.2	2.3
Acc04842.1	Rhamnogalacturonate lyase (Rhamnogalacturonase), Precursor (probable)	-4.3	-6.9	-4.0
Acc05003.1	Probable mannose-1-phosphate guanylyltransferase 2	7.0	6.0	4.1
Acc05631.1	Probable uracil phosphoribosyltransferase (putative)	4.2	4.1	3.8
Acc05734.1	Homeobox-leucine zipper protein HAT7 (HD-ZIP protein 7) (similar to)	4.2	3.5	4.3
Acc05941.1	Tubulin alpha chain	2.3	2.8	2.9
Acc06029.1	DNA polymerase alpha catalytic subunit (putative)	4.7	2.4	2.1
Acc06448.1	Berberine bridge enzyme-like 18 (AtBBE-like 18), Precursor (probable)	2.7	-4.0	-8.1
Acc06786.1	14-3-3-like protein (putative)	3.8	3.8	4.5
Acc06792.1	Protein DMR6-LIKE OXYGENASE 1 (S3H DLO1) (probable)	2.8	4.2	-4.7
Acc07130.1	Anti-sigma-I factor RsgI6 (probable)	-4.9	-6.5	-7.5
Acc07557.1	Calcium-binding protein KIC (similar to)	3.0	3.4	3.6
Acc07679.1	Probable 2-oxoglutarate-dependent dioxygenase AOP1	3.2	5.4	6.3
Acc07922.1	Condensin complex subunit 3 (hCAP-G) (probable)	-6.3	-6.5	-6.1

Acc08329.1	Protein IQ-DOMAIN 14 (probable)	5.7	5.7	4.9
Acc08663.1	RING-H2 finger protein ATL72 (similar to)	2.8	-3.0	-3.1
Acc08757.1	Kinesin-like protein KIN-10A (AtPAKRP2) (putative)	-6.6	-6.2	-6.6
Acc08970.1	Chalcone synthase 1	3.4	4.7	2.2
Acc08998.1	Type III polyketide synthase A (PKS-A) (putative)	2.0	3.4	4.7
Acc09296.1	DNA topoisomerase 2 (putative)	-2.1	-3.2	-2.6
Acc09624.1	CRIB domain-containing protein RIC10 (probable)	-2.6	-3.2	-2.7
Acc09822.1	Mechanosensitive ion channel protein 10 (AtMSL10) (probable)	2.5	5.4	2.6
Acc09955.1	Metallothiol transferase FosB (probable)	-2.0	-3.2	-5.9
Acc10105.1	Dual specificity protein kinase shkD (probable)	5.8	4.3	4.0
Acc10232.1	Transcription factor MYB114 (AtMYB114) (similar to)	4.0	9.3	11.0
Acc10233.1	Organellar oligopeptidase A, chloroplastic/mitochondrial, Precursor (similar to)	2.9	5.3	3.6
Acc10234.1	Organellar oligopeptidase A, chloroplastic/mitochondrial, Precursor (similar to)	3.3	5.8	4.8
Acc10275.1	Formin-like protein 6 (AtFH6), Precursor (similar to)	2.4	6.2	5.8
Acc10479.1	Glutathione S-transferase F12 (AtGSTF12) (putative)	7.9	8.7	9.9
Acc11578.1	Extensin, Precursor (similar to)	6.0	6.1	6.1
Acc11770.1	Protein csh3 (similar to)	5.7	8.6	5.5
Acc11996.1	Cullin-2 (CUL-2) (probable)	-2.9	-3.5	-3.5
Acc12264.1	Dof zinc finger protein DOF1.2 (AtDOF1.2) (probable)	2.0	4.5	4.2
Acc12584.1	Probable 2-oxoglutarate-dependent dioxygenase AOP1.2	5.3	-3.6	-2.1
Acc12612.1	ATP-dependent DNA helicase DDX11 (probable)	-5.7	-5.6	-6.3
Acc12897.1	Calcium sensing receptor, chloroplastic, Precursor (probable)	-2.3	-2.2	-3.3
Acc12965.1	Anthocyanin regulatory C1 protein (similar to)	7.2	8.3	8.4
Acc13290.1	Protein pns1 (probable)	5.0	2.3	2.1
Acc13416.1	Alpha carbonic anhydrase 1, chloroplastic (AtaCA1), Precursor (similar to)	5.0	8.4	2.4
Acc13526.1	G-type lectin S-receptor-like serine/threonine-protein kinase SD1-1 (SD1-1), Precursor (similar to)	-2.6	-3.4	-3.8
Acc13718.1	Nucleoporin NUP159 (probable)	6.5	2.3	6.2
Acc13851.1	Early nodulin-like protein 1 (OsENODL1), Precursor (similar to)	5.4	-3.9	-2.8
Acc14478.1	Blue copper protein (AtBCB), Precursor (probable)	2.2	-5.0	-8.3
Acc14923.1	Ribonuclease Y (RNase Y) (probable)	4.7	4.8	6.7
Acc14972.1	2-alkenal reductase (NADP(+)-dependent) (allyl-ADH1) (probable)	-2.5	-5.3	-4.7
Acc14983.1	Condensin complex subunit 2 (AtCAP-H) (putative)	-3.6	-3.9	-5.4
Acc15351.1	UDP-glycosyltransferase 71K1 (probable)	-2.6	-7.4	-9.4
Acc15400.1	Fe(2+) transport protein 1, Precursor (putative)	4.0	-4.0	-5.3
Acc16298.1	Protein MEI2-like 2 (OML2) (similar to)	5.9	2.1	4.9
Acc16712.1	Probable serine/threonine-protein kinase cdc7	6.4	7.2	2.9
Acc16724.1	Palmitoyl-protein thioesterase 3 (PPT-3), Precursor (probable)	2.8	6.1	6.3
Acc16762.1	Leucoanthocyanidin dioxygenase (LDOX) (putative)	3.8	7.7	8.9

Acc16892.1	Ras-related protein RABC2a (AtRABC2a) (putative)	2.1	2.2	2.7
Acc17128.1	Protein YLS3 (AtXYLP9), Precursor (similar to)	2.1	3.6	4.6
Acc17707.1	Inorganic pyrophosphatase 1 (AtPPsPase1) (putative)	3.4	4.9	7.3
Acc17759.1	Zinc phosphodiesterase ELAC protein 2 (RNase Z 2), Precursor (probable)	-2.4	-4.1	-4.4
Acc17819.1	DNA replication complex GINS protein PSF1 (probable)	3.2	3.4	3.5
Acc17940.1	Nuclear pore complex protein DDB_G0274915 (similar to)	-2.5	-2.9	-2.9
Acc18026.1	Intracellular ribonuclease LX (RNase LX), Precursor (probable)	-2.9	-5.3	-5.4
Acc18031.1	Cytochrome P450 94B3 (similar to)	-4.7	-3.3	-4.7
Acc18426.1	RNA polymerase II subunit A C-terminal domain phosphatase SSU72 (CTD phosphatase SSU72) (probable)	-2.1	-3.1	-3.5
Acc18823.1	Zinc transporter 1, Precursor (putative)	-3.6	5.6	3.3
Acc18949.1	Protein E6 (probable)	-3.5	-9.0	-6.1
Acc19207.1	2-oxoglutarate-dependent dioxygenase DAO (probable)	6.7	-6.6	-6.6
Acc19208.1	2-oxoglutarate-dependent dioxygenase DAO (probable)	6.2	-3.3	-5.6
Acc19415.1	Cytochrome P450 716B1 (probable)	-6.5	-5.8	-5.3
Acc19451.1	Nucleoside diphosphate kinase (NDK) (probable)	5.6	2.1	5.4
Acc19566.1	hypothetical protein	-2.7	-5.5	-6.1
Acc19749.1	Angiomotin-like protein 1 (probable)	3.7	3.3	4.0
Acc19846.1	Probable serine/threonine-protein kinase At1g01540 (putative)	-2.0	-4.1	-2.6
Acc20131.1	Kaempferol 3-O-beta-D-galactosyltransferase (F3GalTase), Precursor (putative)	5.0	7.1	5.9
Acc20132.1	Anthocyanidin 3-O-glucosyltransferase 2 (similar to)	6.5	8.1	7.2
Acc20257.1	Anthocyanidin 5,3-O-glucosyltransferase (putative)	7.3	2.7	3.0
Acc21103.1	Scopoletin glucosyltransferase (putative)	-2.6	-6.1	-7.5
Acc21239.1	Basic blue protein, Precursor (similar to)	3.3	4.3	3.5
Acc21466.1	Transcription factor bHLH149 (AtbHLH149) (similar to)	2.0	3.5	2.2
Acc21795.1	Purple acid phosphatase 17, Precursor (putative)	5.0	5.5	4.4
Acc21806.1	ERAD-associated E3 ubiquitin-protein ligase component HRD3A, Precursor (similar to)	2.9	2.8	3.3
Acc21835.1	LEAF RUST 10 DISEASE-RESISTANCE LOCUS RECEPTOR-LIKE PROTEIN KINASE-like 1.2, Precursor (putative)	2.7	-2.2	-2.2
Acc21864.1	Kinesin-like protein KIN-8A (putative)	2.8	4.0	4.2
Acc22037.1	Cytochrome P450 71A8 (similar to)	-2.1	5.7	9.4
Acc22039.1	Probable galactinol--sucrose galactosyltransferase 2 (putative)	-4.9	-4.1	-5.2
Acc22048.1	Probable pectate lyase 18, Precursor (putative)	2.5	5.0	4.6
Acc22572.1	Lachrymatory-factor synthase, Precursor (probable)	2.6	2.5	2.3
Acc22734.1	Solute carrier family 35 member F2 (probable)	3.4	9.3	6.0
Acc22872.1	Lipid-phosphate phosphatase (probable)	-2.8	-6.8	-8.3
Acc22873.1	Lipid-phosphate phosphatase (probable)	2.5	-2.2	-5.0
Acc23255.1	Rac-like GTP-binding protein ARAC7 (putative)	-4.7	-4.6	-7.2
Acc23748.1	hypothetical protein	-3.0	-4.7	-5.5

Acc23780.1	Putative receptor protein kinase ZmPK1, Precursor (probable)	-5.3	-7.2	-7.5
Acc24129.1	7-deoxyloganetin glucosyltransferase (CrUGT6) (putative)	2.5	6.2	5.6
Acc24301.1	Phytochrome A1 (putative)	2.6	4.9	5.7
Acc24307.1	Transcription factor MYB86 (AtMyb4) (similar to)	-5.2	-3.0	-2.1
Acc24332.1	NDR1/HIN1-Like protein 3 (probable)	2.1	-2.9	-5.4
Acc24569.1	Major allergen Pru ar 1 (putative)	4.1	4.4	2.3
Acc24966.1	Chalcone synthase 2	4.3	7.6	7.8
Acc26222.1	Increased rDNA silencing protein 4 homolog (probable)	-4.0	-2.9	-4.5
Acc26480.1	Probable amino acid permease 7 (similar to)	3.0	4.0	4.1
Acc26789.1	Putative multidrug resistance protein (similar to)	-2.8	-4.7	-4.6
Acc27169.1	Major allergen Pru ar 1 (putative)	2.5	2.6	-2.2
Acc27268.1	Pyruvate, phosphate dikinase, chloroplastic, Precursor (putative)	-3.8	-5.0	-7.6
Acc27430.1	Acetolactate synthase small subunit 1, chloroplastic (AHAS), Precursor (similar to)	2.8	-2.8	-2.2
Acc27584.1	Cyclin-dependent protein kinase inhibitor SMR3 (similar to)	-5.5	-4.7	-5.1
Acc27960.1	21 kDa protein, Precursor (probable)	-2.0	-3.7	-3.1
Acc28618.1	Octanoyltransferase (probable)	-2.7	-3.3	-3.6
Acc28624.1	NADP-dependent malic enzyme (NADP-ME) (putative)	2.7	5.4	2.5
Acc28876.1	Leucoanthocyanidin dioxygenase (LDOX) (similar to)	3.0	6.2	7.5
Acc29052.1	Isoflavone reductase homolog (putative)	5.2	8.7	12.0
Acc29342.1	Protein SHOOT GRAVITROPISM 5 (AtIDD15) (similar to)	-2.7	-2.4	-2.9
Acc29524.1	Serine/threonine-protein kinase Aurora-1 (AtAur1) (putative)	2.7	3.7	5.3
Acc29638.1	Glutamate-1-semialdehyde 2,1-aminomutase, chloroplastic (GSA), Precursor (putative)	2.0	4.6	3.3
Acc29728.1	Pectinesterase 7 (PE 7), Precursor (putative)	5.8	3.3	4.7
Acc30521.1	Peroxidase 51 (Atperox P51), Precursor (putative)	-2.7	-4.9	-3.2
Acc30678.1	hypothetical protein	2.1	-3.4	-2.4
Acc31055.1	Probable calcium-binding protein CML41 (similar to)	3.9	4.7	4.4
Acc31320.1	Bromodomain-containing protein 3 (probable)	2.3	2.8	3.7
Acc31401.1	Myb-related protein B (B-Myb) (probable)	-2.7	-2.4	-3.2
Acc31944.1	Receptor-like kinase TMK4, Precursor (putative)	-4.4	-3.8	-4.5
Acc32155.1	Lysosomal Pro-X carboxypeptidase (PRCP), Precursor (probable)	2.3	-3.1	-3.3
Acc32390.1	Flavonoid 3',5'-hydroxylase 2 (F3'5'H) (putative)	4.1	6.3	2.3
Acc32549.1	Subtilisin-like protease SBT3.18 (AtSBT3.18), Precursor (putative)	3.2	5.0	3.3
Acc32836.1	Nucleobase-ascorbate transporter 7 (AtNAT7) (putative)	2.2	2.7	4.7

Supplementary Table 4. Differentially expressed genes (>2 LogFC) identified from the comparison of *A. purpurea* skin tissues between mature green (MG) stage, colour change (CC) stage, and ripe (RP) stage.

Gene models	Probable description	MG-RP	MG-CC	CC-RP
Acc00147.1	UDP-glucuronate 4-epimerase 6 (AtUGlcAE2) (putative)	4.7	2.6	2.1
Acc00676.1	Mucin-5AC (MUC-5AC), Precursor (probable)	-4.5	-6.5	2.0
Acc00809.1	Photosystem I P700 chlorophyll a apoprotein A2 (similar to)	-4.6	-2.1	-2.4
Acc00999.1	Late embryogenesis abundant protein D-29 (LEA D-29) (probable)	-5.8	-8.4	2.6
Acc01258.1	Systemin receptor SR160, Precursor (putative)	5.1	2.2	2.9
Acc01307.1	hypothetical protein	-2.7	-4.9	2.2
Acc01999.1	F-box protein At2g02240 (probable)	3.1	5.1	-2.1
Acc02113.1	Glucomannan 4-beta-mannosyltransferase 9 (AtCslA9) (putative)	5.0	2.5	2.5
Acc02375.1	Protein ENHANCED DISEASE RESISTANCE 2 (probable)	-2.4	-4.8	2.5
Acc02491.1	Putative calcium-binding protein CML23 (probable)	-2.4	-4.7	2.3
Acc02815.1	Peptide-N4-(N-acetyl-beta-glucosaminyl)asparagine amidase A heavy chain (probable)	5.1	2.7	2.4
Acc02863.1	DBIRD complex subunit ZNF326 (probable)	4.2	2.1	2.1
Acc02870.1	Uncharacterized protein At2g39795, mitochondrial, Precursor (similar to)	2.0	6.1	-4.1
Acc02955.1	Laccase-7, Precursor (putative)	-10.7	-6.2	-4.4
Acc03094.1	Protein RALF-like 24, Precursor (similar to)	10.1	5.1	4.9
Acc03238.1	Caffeic acid 3-O-methyltransferase 1 (CAOMT-1) (similar to)	-6.8	-4.1	-2.7
Acc03239.1	Caffeic acid 3-O-methyltransferase (CAOMT) (similar to)	-6.6	-3.9	-2.8
Acc03241.1	Protein DOWNY MILDEW RESISTANCE 6 (AtDMR6) (putative)	2.0	4.5	-2.5
Acc03373.1	Probable pectate lyase 5, Precursor (putative)	8.3	5.9	2.5
Acc03484.1	Homeobox-leucine zipper protein HDG1 (similar to)	-2.0	-4.3	2.3
Acc03503.1	Dehydration-responsive element-binding protein 1D (Protein DREB1D) (similar to)	-7.5	-10.6	3.0
Acc03614.1	Uncharacterized protein At1g28695, Precursor (similar to)	-6.4	-3.6	-2.7
Acc03877.1	WAT1-related protein At5g07050 (putative)	-5.6	-3.6	-2.1
Acc03890.1	Tubulin beta-4 chain	7.3	5.3	2.1
Acc04524.1	UPF0496 protein At1g20180 (probable)	-6.1	-3.5	-2.6
Acc04573.1	Cytochrome P450 71A1 (probable)	-9.0	-5.8	-3.2
Acc04574.1	Cytochrome P450 71A1 (putative)	-6.2	-2.9	-3.4
Acc04618.1	Glucose-1-phosphate adenylyltransferase large subunit, chloroplastic/amyloplastic, Precursor (putative)	4.6	2.3	2.3
Acc04695.1	Transcription factor MYB44 (AtMYB44) (similar to)	-5.5	-8.8	3.3
Acc05054.1	Beta-galactosidase 16 (Lactase 16), Precursor (putative)	-4.8	-2.4	-2.4
Acc05069.1	Uncharacterized protein Rv3254 (similar to)	4.3	2.0	2.3
Acc05573.1	Putative calcium-binding protein CML19	-4.4	-7.1	2.7

Acc05574.1	Calcium-binding protein CML38 (probable)	-3.5	-6.6	3.1
Acc05774.1	Cyclic nucleotide-gated ion channel 2 (AtCNGC2) (putative)	6.3	4.2	2.1
Acc06060.1	Salicylic acid-binding protein 2 (NtSABP2) (putative)	-7.1	-5.0	-2.1
Acc06315.1	Probable LRR receptor-like serine/threonine-protein kinase At1g14390, Precursor	4.6	2.5	2.1
Acc06512.1	GDSL esterase/lipase At1g29670, Precursor (putative)	6.3	3.4	2.8
Acc06522.1	Probable alpha,alpha-trehalose-phosphate synthase [UDP-forming] 7 (AtTPS7) (putative)	-2.0	-4.2	2.1
Acc06612.1	Nicotianamine synthase (putative)	-3.3	4.4	-7.6
Acc06953.1	Ethylene-responsive transcription factor ERF109 (probable)	-4.4	-8.0	3.6
Acc07000.1	Coiled-coil domain-containing protein 15 (probable)	-2.0	2.8	-4.8
Acc07086.1	GATA transcription factor 5 (probable)	-2.1	-4.1	2.0
Acc07340.1	Polyol transporter 5 (AtPLT5) (similar to)	6.3	4.0	2.3
Acc07347.1	UPF0496 protein At4g34320 (putative)	-8.9	-6.8	-2.1
Acc07523.1	MMS19 nucleotide excision repair protein homolog (probable)	-4.9	-2.8	-2.1
Acc07554.1	Nucleosomal histone kinase 1 (probable)	5.5	2.2	3.2
Acc07786.1	Probable inactive purple acid phosphatase 27, Precursor	-5.2	-3.0	-2.2
Acc07923.1	Nudix hydrolase 21, chloroplastic (AtNUDT21), Precursor (similar to)	-3.0	-5.9	2.9
Acc07936.1	Probable mannitol dehydrogenase (putative)	2.0	6.2	-4.1
Acc08006.1	Lipase-like PAD4 (AtPAD4) (probable)	3.4	5.9	-2.6
Acc08304.1	Light-regulated protein, Precursor (probable)	5.2	2.4	2.8
Acc08533.1	29 kDa ribonucleoprotein A, chloroplastic, Precursor (similar to)	5.1	2.7	2.4
Acc08727.1	Transcription repressor OFP1 (AtOFP1) (probable)	-5.8	-2.9	-3.0
Acc08800.1	UDP-glucuronate 4-epimerase 6 (AtUGlcAE2) (putative)	4.6	2.3	2.3
Acc08824.1	Probable magnesium transporter NIPA1 (putative)	-2.8	-5.1	2.3
Acc09174.1	Delta(8)-fatty-acid desaturase (putative)	-2.5	-4.8	2.3
Acc09189.1	Putative glucose-6-phosphate 1-epimerase (similar to)	5.5	3.1	2.4
Acc09392.1	Protein NRT1/ PTR FAMILY 7.3 (AtNPF7.3) (putative)	-8.7	-5.4	-3.3
Acc09653.1	Topoisomerase I damage affected protein 7 (probable)	2.1	4.8	-2.6
Acc09731.1	Stigma-specific STIG1-like protein 1, Precursor (probable)	-9.1	-6.2	-2.9
Acc09749.1	ATP synthase gamma chain, chloroplastic, Precursor (similar to)	7.4	2.9	4.5
Acc09836.1	MEPFL3, Precursor (similar to)	-5.4	-3.4	-2.0
Acc09959.1	Probable mannitol dehydrogenase (putative)	-5.0	-2.7	-2.3
Acc10335.1	hypothetical protein	-8.5	-6.5	-2.0
Acc10740.1	Natterin-3, Precursor (probable)	6.2	2.1	4.1
Acc10747.1	GATA transcription factor 5 (probable)	-2.9	-5.4	2.5
Acc10770.1	ATP phosphoribosyltransferase regulatory subunit (probable)	2.0	6.4	-4.4
Acc10785.1	Dirigent protein 1 (AtDIR1), Precursor (similar to)	-7.2	-4.0	-3.2
Acc10808.1	Probable aquaporin PIP2-8, N-terminally processed	4.1	6.9	-2.8
Acc11212.1	GDSL esterase/lipase At5g03980, Precursor (probable)	-3.7	-5.7	2.0
Acc11451.1	Carboxymethylenebutenolidase homolog (probable)	8.4	2.4	6.0

Acc11592.1	Abscisic stress-ripening protein 1 (similar to)	-4.5	-2.0	-2.4
Acc12093.1	Serine carboxypeptidase-like 6, Precursor (similar to)	4.7	2.5	2.2
Acc12165.1	Protein Ycf2 (probable)	6.5	2.4	4.1
Acc12243.1	Abscisic stress-ripening protein 2 (similar to)	-5.2	-2.3	-3.0
Acc12244.1	Abscisic stress-ripening protein 3 (probable)	-5.1	-2.2	-2.9
Acc12456.1	Phospholipase D alpha 1 (PLD 1), Precursor (putative)	-11.2	-9.2	-2.0
Acc12703.1	Thioredoxin H1 (AtTrxh1) (putative)	-7.5	-5.1	-2.4
Acc12922.1	Sucrose synthase (putative)	-2.5	-5.6	3.1
Acc12949.1	Protein LURP-one-related 6 (putative)	-7.7	-4.8	-2.9
Acc12988.1	Putative nuclease HARBI1 (probable)	-2.4	-5.0	2.6
Acc13696.1	Protochlorophyllide reductase, chloroplastic (PCR), Precursor (putative)	5.2	2.3	2.9
Acc13781.1	E3 ubiquitin-protein ligase RNF181 (probable)	-7.0	-4.5	-2.5
Acc13876.1	Glutaredoxin-C9 (AtGrxC9) (similar to)	-3.3	-5.4	2.1
Acc14056.1	E3 ubiquitin-protein ligase RHA2A (probable)	-2.0	-4.3	2.2
Acc14314.1	Serine/threonine-protein kinase PBS1 (putative)	-2.5	-5.0	2.5
Acc14521.1	Protein CHLORORESPIRATORY REDUCTION 6, chloroplastic, Precursor (putative)	8.3	3.4	4.9
Acc14527.1	Interleukin-2 receptor subunit beta (IL-2 receptor subunit beta), Precursor (probable)	-4.4	-6.7	2.3
Acc14738.1	Cytochrome b561 and DOMON domain-containing protein At4g12980, Precursor (probable)	6.2	3.5	2.7
Acc14757.1	Photosystem I reaction center subunit XI, chloroplastic (PSI-L), Precursor (putative)	6.4	2.8	3.6
Acc15015.1	hypothetical protein	-7.3	-2.9	-4.3
Acc15333.1	Protein neuralized (probable)	-6.1	-8.8	2.7
Acc15479.1	Probably inactive leucine-rich repeat receptor-like protein kinase At3g28040, Precursor (putative)	8.2	5.5	2.7
Acc15507.1	hypothetical protein	-2.6	-4.9	2.2
Acc15710.1	Protein IQ-DOMAIN 14 (probable)	4.4	2.2	2.2
Acc15717.1	Sugar transport protein 1 (putative)	-8.3	-5.3	-3.0
Acc15718.1	Ethylene-responsive transcription factor ERF026 (probable)	-4.2	-6.6	2.4
Acc15719.1	Dehydration-responsive element-binding protein 1E (Protein DREB1E) (probable)	-6.2	-8.6	2.3
Acc15720.1	Dehydration-responsive element-binding protein 1D (Protein DREB1D) (probable)	-4.5	-7.6	3.2
Acc15721.1	Dehydration-responsive element-binding protein 1A (Protein DREB1A) (probable)	-7.6	-9.8	2.3
Acc15722.1	Dehydration-responsive element-binding protein 1D (Protein DREB1D) (similar to)	-5.9	-8.6	2.7
Acc15723.1	Dehydration-responsive element-binding protein 1D (Protein DREB1D) (probable)	-4.7	-7.3	2.6
Acc15763.1	Myosin-52 (probable)	7.7	3.5	4.1
Acc16437.1	Ribulose bisphosphate carboxylase/oxygenase activase 2, chloroplastic (RA 2), Precursor (putative)	6.0	3.5	2.5
Acc16694.1	BOI-related E3 ubiquitin-protein ligase 1 (probable)	4.6	2.4	2.2

Acc16752.1	Phosvitin, Precursor (probable)	5.1	2.5	2.5
Acc17065.1	Polyamine oxidase 1 (AtPAO1) (putative)	-2.4	-4.9	2.5
Acc17167.1	Protein NRT1/ PTR FAMILY 7.3 (AtNPF7.3) (putative)	-7.4	-4.2	-3.3
Acc17416.1	hypothetical protein	5.6	3.0	2.6
Acc17439.1	Gibberellin 2-beta-dioxygenase 8 (putative)	-4.1	-6.6	2.4
Acc17460.1	ATP synthase gamma chain 1, chloroplastic, Precursor (similar to)	6.8	3.6	3.2
Acc17475.1	Lactate utilization protein B (probable)	-10.5	-7.4	-3.1
Acc17476.1	Stigma-specific STIG1-like protein 1, Precursor (probable)	-9.0	-6.2	-2.8
Acc17811.1	Protein TIFY 5A (probable)	-5.5	-8.0	2.4
Acc17930.1	Cinnamoyl-CoA reductase 1 (AtCCR1) (similar to)	-2.3	3.7	-6.1
Acc18055.1	Up-regulator of cell proliferation (probable)	-2.1	-4.3	2.2
Acc18063.1	Adenine/guanine permease AZG2 (AtAzg2) (putative)	-7.9	-4.2	-3.7
Acc18122.1	Homeobox protein knotted-1-like 6 (similar to)	5.2	2.9	2.3
Acc18235.1	Protein NRT1/ PTR FAMILY 4.6 (AtNPF4.6) (similar to)	7.3	2.0	5.2
Acc18347.1	Cleavage and polyadenylation specificity factor subunit 6 (probable)	4.3	2.0	2.3
Acc18381.1	UDP-glycosyltransferase 89B2 (similar to)	-3.1	-5.5	2.4
Acc18581.1	Beta-glucosidase 44 (AtBGLU44), Precursor (putative)	6.9	4.8	2.1
Acc18823.1	Zinc transporter 1, Precursor (putative)	-2.1	6.2	-8.3
Acc19298.1	Peptidyl-prolyl cis-trans isomerase FKBP16-1, chloroplastic (PPIase FKBP16-1), Precursor (similar to)	6.6	2.5	4.1
Acc19462.1	Calcium-binding protein PBP1 (similar to)	-2.8	-5.0	2.2
Acc19465.1	Indole-3-acetic acid-amido synthetase GH3.6 (AtGH3-6) (putative)	-7.6	-5.3	-2.2
Acc19481.1	Proline-rich protein 30 (probable)	5.3	3.1	2.2
Acc19484.1	Protochlorophyllide reductase, chloroplastic (PCR), Precursor (putative)	6.3	2.2	4.1
Acc19500.1	Auxin-induced protein 22D (putative)	-3.7	-6.7	3.0
Acc19822.1	Delta(8)-fatty-acid desaturase (putative)	-2.5	-4.6	2.1
Acc19972.1	Dehydration-responsive element-binding protein 1A (Protein DREB1A) (similar to)	-5.3	-9.0	3.6
Acc19973.1	Sugar transport protein 1 (putative)	-12.5	-10.2	-2.3
Acc20097.1	Glucan endo-1,3-beta-glucosidase 11 ((1->3)-beta-glucanase 11), Precursor (similar to)	5.8	2.3	3.5
Acc20266.1	Probable membrane-associated kinase regulator 2	5.3	3.1	2.1
Acc20331.1	Oligopeptide transporter 6 (AtOPT6) (putative)	5.3	2.2	3.1
Acc20356.1	NDR1/HIN1-like protein 12 (probable)	5.8	2.6	3.3
Acc20373.1	UNC93-like protein 1 (putative)	-2.7	-5.8	3.1
Acc20640.1	E3 ubiquitin-protein ligase RMA1H1 (probable)	-3.1	-5.8	2.7
Acc20661.1	Protein E6 (similar to)	6.1	2.9	3.1
Acc21087.1	Basic secretory protease (BBSP, partial [Boswellia serrata]) (similar to)	-6.0	-3.0	-3.0
Acc21100.1	Cysteine proteinase RD21A (RD21), Precursor (putative)	-4.9	-2.8	-2.0
Acc21125.1	Ethylene-responsive transcription factor ERF109 (probable)	-5.7	-8.8	3.1
Acc21556.1	Feruloyl CoA ortho-hydroxylase 2 (probable)	-8.3	-3.9	-4.4

Acc21698.1	Aclacinomycin 10-hydroxylase RdmB (probable)	5.3	2.9	2.3
Acc22162.1	SNF1-related protein kinase regulatory subunit gamma-like PV42a (AtPV42a) (probable)	-2.5	-4.7	2.2
Acc22506.1	3-ketoacyl-CoA synthase 11 (KCS-11) (putative)	-3.5	-5.5	2.0
Acc22643.1	Abscisic acid 8'-hydroxylase 1 (ABA 8'-hydroxylase 1) (putative)	-2.4	-4.9	2.5
Acc22754.1	Proteinase inhibitor (putative)	-6.9	-4.3	-2.6
Acc22824.1	Protocadherin-15, Precursor (probable)	-6.8	-4.1	-2.7
Acc22908.1	Transcription factor MYB108 (AtMYB108) (putative)	-11.6	-8.7	-2.9
Acc23042.1	Protein Rev (probable)	2.5	5.9	-3.4
Acc23223.1	Probable sulfate transporter 3.5 (putative)	-10.2	-6.4	-3.8
Acc23237.1	Polyol transporter 5 (AtPLT5) (similar to)	-6.1	-3.4	-2.6
Acc23350.1	Putative nuclease HARBI1 (probable)	-2.5	-5.0	2.5
Acc23416.1	Auxin-responsive protein SAUR72 (probable)	6.3	4.2	2.0
Acc23687.1	BEL1-like homeodomain protein 4 (BEL1-like protein 4) (probable)	6.2	2.0	4.1
Acc23693.1	Allene oxide synthase 1, chloroplastic (LeAOS1), Precursor (putative)	4.9	2.4	2.6
Acc23709.1	Transcription factor MYB44 (AtMYB44) (similar to)	-3.7	-6.2	2.5
Acc23710.1	Protein MIZU-KUSSEI 1 (probable)	-9.1	-4.7	-4.4
Acc23891.1	Flowering time control protein FY (probable)	-3.1	-6.1	3.0
Acc23914.1	4-diphosphocytidyl-2-C-methyl-D-erythritol kinase (CMK) (probable)	-5.2	-2.7	-2.5
Acc24872.1	RING-H2 finger protein ATL33 (probable)	5.4	2.8	2.6
Acc24939.1	Glucomannan 4-beta-mannosyltransferase 9 (AtCslA9) (putative)	4.6	2.6	2.0
Acc25260.1	Tetrahydrocannabinolic acid synthase (THCAS), Precursor (probable)	-5.0	-2.9	-2.1
Acc25261.1	Tetrahydrocannabinolic acid synthase (THCAS), Precursor (probable)	-6.0	-3.8	-2.2
Acc25471.1	hypothetical protein	-2.4	-5.0	2.6
Acc25516.1	Beta-glucosidase 11 (AtBGLU11), Precursor (putative)	2.2	4.8	-2.6
Acc25690.1	Putative F-box/LRR-repeat protein At1g56400 (probable)	-7.1	-4.6	-2.5
Acc25731.1	Photosystem I reaction center subunit XI, chloroplastic (PSI-L), Precursor (putative)	4.3	2.1	2.3
Acc25752.1	Calcium uniporter protein 6, mitochondrial, Precursor (similar to)	-3.1	-5.1	2.0
Acc26280.1	Cellulose synthase-like protein E1 (AtCslE1) (putative)	2.9	5.1	-2.2
Acc26332.1	Centrosomal protein of 89 kDa (Cep89) (probable)	-6.9	-3.8	-3.1
Acc26349.1	COBRA-like protein 10, Precursor (putative)	-5.8	-3.7	-2.1
Acc26372.1	Indole-3-acetic acid-amido synthetase GH3.6 (AtGH3-6) (putative)	-10.7	-6.0	-4.6
Acc26592.1	Probable non-specific lipid-transfer protein AKCS9 (LTP), Precursor (putative)	-6.5	-3.3	-3.2
Acc27100.1	Transcription factor MYB108 (AtMYB108) (similar to)	-4.8	-2.4	-2.4
Acc27237.1	Monothiol glutaredoxin-S10 (AtGrxS10) (putative)	-9.4	-7.3	-2.0

Acc27645.1	Probable RNA-binding protein ARP1	-6.5	-3.4	-3.1
Acc27890.1	Cytochrome P450 714A1 (putative)	-2.1	-4.2	2.1
Acc27991.1	Protein HOTHEAD, Precursor (similar to)	-5.8	-3.6	-2.2
Acc28015.1	Dehydration-responsive element-binding protein 1D (Protein DREB1D) (putative)	-5.5	-8.6	3.1
Acc28137.1	S-norcoclaurine synthase 1 (CjNCS1) (putative)	-8.7	-4.9	-3.8
Acc28207.1	Probable aspartic protease At2g35615, Precursor	4.4	2.2	2.2
Acc28230.1	Cytochrome P450 monooxygenase paxP (probable)	5.3	2.8	2.5
Acc28533.1	Protein ASPARTIC PROTEASE IN GUARD CELL 1 (AtASPG1), Precursor (putative)	5.1	2.3	2.8
Acc28663.1	UDP-sulfoquinovose synthase, chloroplastic, Precursor (putative)	4.4	2.3	2.1
Acc28990.1	Glycine cleavage system H protein, mitochondrial, Precursor (putative)	7.0	2.4	4.6
Acc29099.1	Homeobox protein ATH1 (probable)	-7.1	-4.9	-2.2
Acc30030.1	Probable CCR4-associated factor 1 homolog 9 (putative)	-2.2	-4.3	2.1
Acc30157.1	Protein ENHANCED DISEASE RESISTANCE 2-like (probable)	4.8	2.8	2.0
Acc30541.1	Putative calcium-binding protein CML19 (probable)	-4.1	-7.0	2.9
Acc30843.1	Cytokinin hydroxylase (putative)	9.9	5.0	4.9
Acc30956.1	BTB/POZ and TAZ domain-containing protein 4 (putative)	-3.0	-5.3	2.3
Acc30960.1	Peroxidase 51 (Atperox P51), Precursor (putative)	-7.8	-4.1	-3.7
Acc31261.1	ABC transporter G family member 21 (ABC transporter ABCG.21) (putative)	-5.9	-3.8	-2.2
Acc31306.1	CLE27p, Precursor (probable)	6.4	2.2	4.2
Acc32047.1	F-box/FBD/LRR-repeat protein At5g22660 (probable)	-5.1	-2.0	-3.1
Acc32410.1	E3 ubiquitin-protein ligase CIP8 (probable)	-2.2	-5.0	2.8
Acc33159.1	Protein transport protein YIP1 (probable)	-8.1	-5.2	-2.9
Acc33160.1	Protein transport protein YIP1 (probable)	-7.2	-4.4	-2.8
Acc33184.1	Transcription factor MYB108 (AtMYB108) (putative)	-7.8	-4.8	-3.1
Acc33342.1	Protein MARD1 (probable)	4.9	2.6	2.3
Acc33447.2	Renalase (probable)	7.6	2.4	5.2

Supplementary Table 5. Differentially expressed genes (>2 LogFC) identified from the comparison of *A. purpurea* flesh tissues between mature green (MG) stage, colour change (CC) stage, and ripe (RP) stage.

Gene models	Probable description	MG-RP	MG-CC	CC-RP
Acc00147.1	UDP-glucuronate 4-epimerase 6 (AtUGlcAE2) (putative)	5.2	3.2	2.0
Acc01051.1	GATA transcription factor 5 (probable)	-5.7	-3.5	-2.2
Acc01092.1	Aspartyl protease family protein 1, Precursor (similar to)	6.1	3.8	2.3
Acc01744.1	Glycerol-3-phosphate 2-O-acyltransferase 6 (AtGPAT6) (putative)	-3.3	-5.6	2.4
Acc01756.1	ATP-dependent Clp protease proteolytic subunit (probable)	-7.3	-4.0	-3.3
Acc01770.1	Vacuolar cation/proton exchanger 3 (putative)	9.4	6.9	2.5
Acc02419.1	Protein KINESIN LIGHT CHAIN-RELATED 2 (AtKLC1) (putative)	-5.9	-3.9	-2.0
Acc02702.1	Ethylene-responsive transcription factor ERF025 (probable)	-3.1	-5.9	2.8
Acc02955.1	Laccase-7, Precursor (putative)	-5.6	-2.7	-2.8
Acc03005.1	Nodulation-signaling pathway 2 protein (probable)	-4.0	-7.3	3.2
Acc03025.1	Solute carrier family 40 member 2 (AtIREG2) (putative)	6.3	3.0	3.2
Acc03238.1	Caffeic acid 3-O-methyltransferase 1 (CAOMT-1) (similar to)	-6.9	-3.0	-3.8
Acc03239.1	Caffeic acid 3-O-methyltransferase (CAOMT) (similar to)	-7.1	-3.3	-3.8
Acc03298.1	Pectinesterase 34 (PE 34) (similar to)	5.1	2.7	2.3
Acc03324.1	Sterol 24-C-methyltransferase erg6 (probable)	4.9	2.7	2.2
Acc03373.1	Probable pectate lyase 5, Precursor (putative)	9.0	5.9	3.1
Acc03503.1	Dehydration-responsive element-binding protein 1D (Protein DREB1D) (similar to)	-5.1	-9.6	4.4
Acc03552.1	Protein EXORDIUM-like 3, Precursor (putative)	6.2	4.2	2.0
Acc04243.1	Slit homolog 2 protein C-product, Precursor (probable)	-5.3	-2.6	-2.8
Acc04294.1	Photosystem I reaction center subunit II, chloroplastic (PSI-D), Precursor (putative)	5.0	2.1	2.9
Acc04480.1	Transport and Golgi organization 2 homolog (probable)	-7.0	-2.2	-4.8
Acc04499.1	hypothetical protein	-7.7	-3.8	-3.9
Acc04564.1	Auxin-responsive protein SAUR50 (putative)	-5.9	-3.6	-2.4
Acc04573.1	Cytochrome P450 71A1 (probable)	-7.6	-3.8	-3.8
Acc04574.1	Cytochrome P450 71A1 (putative)	-7.2	-3.6	-3.6
Acc04608.1	BEL1-like homeodomain protein 6 (BEL1-like protein 6) (similar to)	-2.5	-5.1	2.6
Acc04799.1	Phytosulfokine-beta, Precursor (similar to)	-4.9	-2.8	-2.0
Acc05136.1	Chlorophyll a-b binding protein CP26, chloroplastic, Precursor (putative)	4.9	2.2	2.7
Acc05774.1	Cyclic nucleotide-gated ion channel 2 (AtCNGC2) (putative)	8.0	4.2	3.7
Acc06071.1	Oxygen-evolving enhancer protein 1, chloroplastic (OEE1), Precursor (putative)	6.2	2.4	3.7
Acc06072.1	Oxygen-evolving enhancer protein 1, chloroplastic (OEE1), Precursor (putative)	6.1	2.8	3.3

Acc06173.1	Chlorophyll a-b binding protein 7, chloroplastic, Precursor (putative)	5.1	2.7	2.4
Acc06220.1	Galactinol synthase 1 (ArGolS1) (putative)	7.9	2.2	5.7
Acc06272.1	Probable galacturonosyltransferase-like 9 (similar to)	-5.0	-2.6	-2.3
Acc06452.1	Photosystem I reaction center subunit psaK, chloroplastic, Precursor (similar to)	7.2	2.2	5.0
Acc06686.1	Ferredoxin, chloroplastic, Precursor (putative)	5.3	2.9	2.4
Acc06753.1	Protein DMR6-LIKE OXYGENASE 2 (S3H DLO2) (probable)	6.1	3.6	2.5
Acc06784.1	ABC transporter G family member 11 (ABC transporter ABCG.11) (probable)	5.8	2.4	3.4
Acc06791.1	Protein DMR6-LIKE OXYGENASE 1 (S3H DLO1) (probable)	7.9	3.8	4.1
Acc06792.1	Protein DMR6-LIKE OXYGENASE 1 (S3H DLO1) (probable)	7.0	3.4	3.6
Acc06953.1	Ethylene-responsive transcription factor ERF109 (probable)	-3.7	-7.0	3.3
Acc06999.1	Formin-like protein 16, Precursor (probable)	-7.9	-2.1	-5.8
Acc07074.1	Regulatory protein ADR1 (probable)	-11.7	-6.1	-5.6
Acc07226.1	(RS)-norcoclaurine 6-O-methyltransferase (Ps6OMT) (probable)	-7.1	-3.8	-3.3
Acc07340.1	Polyol transporter 5 (AtPLT5) (similar to)	5.9	2.9	3.0
Acc07415.1	Probable indole-3-acetic acid-amido synthetase GH3.1 (AtGH3-1) (putative)	-6.9	-4.3	-2.6
Acc07453.1	3-ketoacyl-CoA synthase 2 (KCS-2) (probable)	-10.3	-5.5	-4.8
Acc07554.1	Nucleosomal histone kinase 1 (probable)	6.0	2.6	3.3
Acc08183.1	Glutamate--glyoxylate aminotransferase 2 (AtGGT2) (putative)	4.9	2.5	2.3
Acc08260.1	Chlorophyll a-b binding protein 7, chloroplastic, Precursor (putative)	6.1	2.8	3.3
Acc08591.1	Uncharacterized protein At2g37660, chloroplastic, Precursor (similar to)	5.0	2.2	2.8
Acc08596.1	Protein FAR-RED ELONGATED HYPOCOTYL 1 (probable)	2.1	5.9	-3.8
Acc08653.1	Trihelix transcription factor GT-3b (probable)	-5.9	-3.3	-2.5
Acc08727.1	Transcription repressor OFP1 (AtOFP1) (probable)	-8.8	-4.5	-4.3
Acc08759.1	Long-chain-alcohol oxidase FAO1 (putative)	-6.2	-3.3	-2.9
Acc08788.1	Xyloglucan endotransglucosylase/hydrolase 2, Precursor (putative)	6.3	2.5	3.8
Acc08800.1	UDP-glucuronate 4-epimerase 6 (AtUGlcAE2) (putative)	5.9	3.7	2.1
Acc09018.1	Putative lipid-binding protein At4g00165, Precursor	-6.1	-3.0	-3.1
Acc09044.1	Methylsterol monooxygenase 1-1 (AtSMO1-1) (putative)	6.9	2.8	4.0
Acc09155.1	Probable polygalacturonase (PG) (putative)	5.6	2.8	2.9
Acc09699.1	Acyl-CoA--sterol O-acyltransferase 1 (probable)	-2.5	-4.5	2.0
Acc09731.1	Stigma-specific STIG1-like protein 1, Precursor (probable)	-7.6	-4.8	-2.8
Acc09843.1	Cytokinin dehydrogenase 3 (AtCKX3), Precursor (putative)	-8.4	-3.8	-4.7

Acc10050.1	Peptidyl-prolyl cis-trans isomerase CYP28, chloroplastic (PPIase CYP28), Precursor (similar to)	8.9	4.9	4.0
Acc10472.1	2-oxoglutarate-dependent dioxygenase DAO (putative)	5.5	3.4	2.1
Acc10519.1	Protein NETWORKED 2D (probable)	2.2	6.4	-4.2
Acc10699.1	Germin-like protein 5-1, Precursor (putative)	5.3	2.5	2.8
Acc10808.1	Probable aquaporin PIP2-8, N-terminally processed	3.7	5.9	-2.2
Acc10928.1	Heavy metal-associated isoprenylated plant protein 2 (AtHIP02), Precursor (similar to)	7.7	2.7	5.0
Acc11515.1	LysM domain-containing GPI-anchored protein 1, Precursor (putative)	4.8	2.6	2.2
Acc11629.1	Laccase-14, Precursor (putative)	-4.4	-2.2	-2.2
Acc11638.1	hypothetical protein	-6.7	-4.2	-2.5
Acc11969.1	Protein PLASTID MOVEMENT IMPAIRED 1 (putative)	5.0	2.3	2.7
Acc12093.1	Serine carboxypeptidase-like 6, Precursor (similar to)	4.5	2.4	2.1
Acc12243.1	Abscisic stress-ripening protein 2 (similar to)	-6.5	-3.3	-3.1
Acc12244.1	Abscisic stress-ripening protein 3 (probable)	-4.8	-2.1	-2.8
Acc12245.1	Abscisic stress-ripening protein 1 (probable)	-6.7	-4.7	-2.1
Acc12268.1	Pathogen-related protein (putative)	5.5	3.3	2.2
Acc12315.1	LysM domain-containing GPI-anchored protein 1, Precursor (similar to)	7.4	3.4	4.0
Acc12539.1	Cucumber peeling cupredoxin (CPC) (probable)	6.1	3.1	3.0
Acc12589.1	Auxin-responsive protein IAA17 (similar to)	-7.5	-4.3	-3.2
Acc12655.1	Probable sugar phosphate/phosphate translocator At1g12500 (putative)	5.6	2.2	3.4
Acc12814.1	Subtilisin-like protease SBT1.4 (AtSBT1.4), Precursor (putative)	6.5	3.6	2.9
Acc12922.1	Sucrose synthase (putative)	-2.2	-4.6	2.4
Acc12937.1	Protein MARD1 (probable)	-5.8	-3.5	-2.3
Acc13168.1	Zinc finger homeobox protein 4 (ZFH-4) (probable)	5.2	2.3	2.9
Acc13246.1	Calmodulin-binding protein 25 (AtCAMBP25) (probable)	4.2	2.0	2.2
Acc13760.1	Paired amphipathic helix protein Sin3-like 3 (AtSin3) (probable)	-8.6	-3.8	-4.7
Acc13781.1	E3 ubiquitin-protein ligase RNF181 (probable)	-7.0	-4.2	-2.8
Acc14243.1	Photosystem II protein psbY-2, chloroplastic, Precursor (probable)	5.2	2.6	2.6
Acc14402.1	Homeobox-leucine zipper protein HAT14 (HD-ZIP protein 14) (similar to)	2.1	5.9	-3.7
Acc14463.1	SPX domain-containing protein 2 (AtSPX2) (putative)	3.1	5.8	-2.7
Acc14757.1	Photosystem I reaction center subunit XI, chloroplastic (PSI-L), Precursor (putative)	8.5	2.5	6.0
Acc14779.1	Ycf54-like protein (probable)	4.6	2.5	2.1
Acc14798.1	Peroxidase 4, Precursor (putative)	-5.0	-2.6	-2.4
Acc15015.1	hypothetical protein	-7.8	-2.9	-4.9
Acc15145.1	Photosystem II reaction center W protein, chloroplastic, Precursor (probable)	6.7	2.7	4.1

Acc15148.1	Phospholipase A1-Igamma1, chloroplastic, Precursor (similar to)	5.8	2.0	3.8
Acc15281.1	E3 ubiquitin-protein ligase RBBP6 (probable)	6.8	4.0	2.8
Acc15479.1	Probably inactive leucine-rich repeat receptor-like protein kinase At3g28040, Precursor (putative)	9.3	5.7	3.7
Acc15517.1	Endoglucanase 1, Precursor (similar to)	6.5	2.4	4.1
Acc15528.1	hypothetical protein	-6.9	-4.4	-2.5
Acc15717.1	Sugar transport protein 1 (putative)	-8.0	-5.9	-2.2
Acc15766.1	Cell wall protein DAN4, Precursor (probable)	-4.9	-2.8	-2.1
Acc16389.1	Probable polygalacturonase (PG)	4.6	2.4	2.1
Acc17475.1	Lactate utilization protein B (probable)	-7.2	-4.7	-2.5
Acc17804.1	F-box/kelch-repeat protein At1g15670 (probable)	-5.0	-2.9	-2.1
Acc17811.1	Protein TIFY 5A (probable)	-3.3	-6.7	3.4
Acc17986.1	Sacsin (DNAJC29) (probable)	6.0	3.6	2.4
Acc18063.1	Adenine/guanine permease AZG2 (AtAzg2) (putative)	-7.7	-5.2	-2.5
Acc18122.1	Homeobox protein knotted-1-like 6 (similar to)	4.9	2.6	2.3
Acc18178.1	Protein EDS1B (probable)	5.1	2.5	2.6
Acc18331.1	Flavonoid 3'-monooxygenase (putative)	4.1	2.1	2.0
Acc18480.1	Reticulon-like protein B21 (AtRTNLB21) (probable)	7.1	3.1	4.1
Acc18800.1	Protein NRT1/ PTR FAMILY 4.5 (AtNPF4.5) (probable)	6.6	4.1	2.4
Acc18867.1	Cytidylate kinase (probable)	8.7	4.7	4.0
Acc18940.1	Fasciclin-like arabinogalactan protein 13, Precursor (similar to)	4.9	2.9	2.0
Acc18954.1	Frizzled and smoothened-like protein A, Precursor (probable)	-4.9	-2.6	-2.3
Acc19029.1	GTPase-activating protein GYP3 (similar to)	-7.3	-4.8	-2.4
Acc19030.1	GTPase-activating protein GYP3 (similar to)	-7.2	-4.9	-2.3
Acc19452.1	conserved hypothetical protein	-6.0	-3.3	-2.7
Acc19500.1	Auxin-induced protein 22D (putative)	-2.8	-5.3	2.5
Acc19625.1	hypothetical protein	-2.7	-5.1	2.4
Acc19749.1	Angiomotin-like protein 1 (probable)	-7.1	-4.9	-2.2
Acc19973.1	Sugar transport protein 1 (putative)	-12.7	-10.6	-2.1
Acc20097.1	Glucan endo-1,3-beta-glucosidase 11 ((1->3)-beta-glucanase 11), Precursor (similar to)	7.7	3.5	4.2
Acc20166.1	Cinnamoyl-CoA reductase-like SNL6 (probable)	-5.5	-3.5	-2.0
Acc20266.1	Probable membrane-associated kinase regulator 2	7.1	4.2	2.9
Acc20356.1	NDR1/HIN1-like protein 12 (probable)	6.4	3.2	3.2
Acc20412.1	Cinnamoyl-CoA reductase-like SNL6 (probable)	-5.6	-3.3	-2.3
Acc20661.1	Protein E6 (similar to)	5.4	2.8	2.5
Acc20680.1	Pathogenesis-related genes transcriptional activator PTI5 (similar to)	-6.4	-3.4	-3.1
Acc21087.1	Basic secretory protease (BBSP, partial [Boswellia serrata]) (similar to)	-6.3	-3.8	-2.5
Acc21125.1	Ethylene-responsive transcription factor ERF109 (probable)	-2.2	-5.8	3.6
Acc21429.1	F-box protein PP2-B15 (AtPP2-B15) (probable)	-3.3	5.4	-8.6
Acc21505.1	F-box protein At3g44326 (probable)	-6.5	-4.2	-2.2

Acc21556.1	Feruloyl CoA ortho-hydroxylase 2 (probable)	-7.3	-3.7	-3.6
Acc21630.1	Mediator of RNA polymerase II transcription subunit 29 (probable)	-7.0	-3.1	-3.9
Acc21720.1	Early nodulin-like protein 2, Precursor (probable)	7.4	3.6	3.8
Acc22123.1	ACT domain-containing protein ACR11, Precursor (similar to)	4.7	2.5	2.3
Acc22338.1	Linoleate 13S-lipoxygenase 3-1, chloroplastic, Precursor (putative)	-5.2	-3.0	-2.2
Acc22414.1	Aspartyl protease family protein At5g10770, Precursor (putative)	4.8	2.6	2.2
Acc22511.1	Pleiotropic drug resistance protein 1 (similar to)	6.6	2.6	4.0
Acc22538.1	Two-on-two hemoglobin-3 (AtGLB3) (similar to)	-3.6	3.4	-7.0
Acc22685.1	Taraxerol synthase (KdTAS) (similar to)	5.7	2.4	3.3
Acc22777.1	Sulfate transporter 3.1 (putative)	-7.8	-4.1	-3.7
Acc22824.1	Protocadherin-15, Precursor (probable)	-7.7	-3.1	-4.5
Acc22908.1	Transcription factor MYB108 (AtMYB108) (putative)	-9.1	-5.9	-3.2
Acc23246.1	Dynein light chain LC6, flagellar outer arm (probable)	5.4	3.2	2.2
Acc23323.1	1-aminocyclopropane-1-carboxylate oxidase 1 (ACC oxidase 1) (putative)	-9.6	-6.8	-2.8
Acc23617.1	Cytokinin dehydrogenase 5 (AtCKX5), Precursor (putative)	-6.0	-3.5	-2.5
Acc23710.1	Protein MIZU-KUSSEI 1 (probable)	-8.1	-3.1	-5.0
Acc23756.1	Probable serine/threonine-protein kinase At1g01540 (putative)	6.8	3.5	3.3
Acc23803.1	Zinc finger protein ZAT6 (probable)	-4.6	-2.4	-2.2
Acc23829.1	Phytosulfokine-beta, Precursor (probable)	-9.5	-7.2	-2.3
Acc23840.1	Probable xyloglucan glycosyltransferase 12 (AtCslC12) (putative)	5.1	2.4	2.7
Acc24063.1	Basic blue protein (similar to)	5.1	2.9	2.2
Acc24164.1	Chlorophyll a-b binding protein CP26, chloroplastic, Precursor (putative)	6.4	2.3	4.2
Acc24189.1	Nicotianamine synthase (putative)	-3.3	2.6	-5.9
Acc24394.1	Probable acyl-activating enzyme 1, peroxisomal (AtAMPBP1) (putative)	4.3	2.1	2.2
Acc24484.1	Aspartic proteinase A2 (AtASP57), Precursor (similar to)	7.4	2.6	4.8
Acc24523.1	Transcription factor MYB108 (AtMYB108) (similar to)	-6.2	-3.3	-2.9
Acc24610.1	Germin-like protein 9-3, Precursor (similar to)	4.4	2.3	2.1
Acc24939.1	Glucomannan 4-beta-mannosyltransferase 9 (AtCslA9) (putative)	6.7	3.4	3.2
Acc25119.1	Triose phosphate/phosphate translocator TPT, chloroplastic, Precursor (putative)	4.1	2.1	2.0
Acc25199.1	Chlorophyll a-b binding protein 3C, chloroplastic (LHCP), Precursor (putative)	5.2	2.7	2.4
Acc25200.1	Chlorophyll a-b binding protein 40, chloroplastic (LHCP), Precursor (similar to)	5.2	3.2	2.1
Acc25594.1	Photosystem II reaction center W protein, chloroplastic, Precursor (similar to)	7.5	3.0	4.4

Acc25729.1	Nuclear transition protein 2 (TP-2) (probable)	-2.5	2.0	-4.5
Acc25860.1	Expansin-A4 (AtEXPA4), Precursor (putative)	5.8	3.7	2.0
Acc26166.1	Photosystem II 5 kDa protein, chloroplastic, Precursor [Petunia x hybrida] (probable)	6.6	2.8	3.8
Acc26216.1	F-box protein At2g27310 (probable)	-8.9	-5.8	-3.1
Acc26263.1	Auxin-responsive protein SAUR72 (similar to)	8.2	2.6	5.6
Acc26332.1	Centrosomal protein of 89 kDa (Cep89) (probable)	-7.0	-2.9	-4.2
Acc26372.1	Indole-3-acetic acid-amido synthetase GH3.6 (AtGH3-6) (putative)	-8.0	-4.0	-4.0
Acc26404.1	Protochlorophyllide reductase, chloroplastic (PCR), Precursor (putative)	5.5	3.1	2.4
Acc26411.1	Thioredoxin M4, chloroplastic (AtTrxm4), Precursor (putative)	4.8	2.6	2.1
Acc26426.1	Probable aminotransferase TAT2 (putative)	7.4	2.9	4.6
Acc26648.1	ABC transporter B family member 25 (ABC transporter ABCB.25) (putative)	4.7	2.7	2.1
Acc26651.1	Protein LONGIFOLIA 1 (probable)	4.2	2.1	2.1
Acc26748.1	Probable inactive poly [ADP-ribose] polymerase SRO2	-7.4	-3.1	-4.4
Acc26942.1	Protein IQ-DOMAIN 14 (probable)	-3.6	-6.0	2.3
Acc27059.1	Aspartic proteinase, Precursor (probable)	7.7	2.6	5.1
Acc27295.1	Photosystem I reaction center subunit II, chloroplastic (PSI-D), Precursor (putative)	5.1	2.0	3.1
Acc27688.1	Aquaporin TIP1-3, N-terminally processed (putative)	6.5	3.7	2.9
Acc28015.1	Dehydration-responsive element-binding protein 1D (Protein DREB1D) (putative)	-4.8	-8.5	3.6
Acc28207.1	Probable aspartic protease At2g35615, Precursor	7.8	3.0	4.8
Acc28523.1	IST1 homolog (hIST1) (probable)	-4.5	-2.0	-2.4
Acc28603.1	Aquaporin TIP4-1, N-terminally processed (putative)	8.5	6.1	2.4
Acc29133.1	Protein NRT1/ PTR FAMILY 1.2 (AtNPF1.2) (putative)	7.6	2.9	4.7
Acc29414.1	Protein ASPARTIC PROTEASE IN GUARD CELL 1 (AtASPG1), Precursor (putative)	5.3	3.0	2.3
Acc29506.1	Zinc transporter 4, chloroplastic, Precursor (putative)	-2.8	5.4	-8.2
Acc29538.1	Protein lingerer (probable)	-5.2	-2.9	-2.4
Acc30819.1	Proline dehydrogenase 1, mitochondrial, Precursor (similar to)	-8.3	-5.3	-3.0
Acc30921.1	Zinc finger protein 329 (probable)	-8.2	-5.7	-2.5
Acc30960.1	Peroxidase 51 (Atperox P51), Precursor (putative)	-11.0	-6.9	-4.1
Acc31022.1	Patatin-like protein 2 (AtPLP2) (putative)	-2.8	-5.4	2.6
Acc31047.1	Oxygen-evolving enhancer protein 1, chloroplastic (OEE1), Precursor (putative)	5.3	2.6	2.8
Acc31158.1	Probable peroxygenase 4 (AtPXG4) (similar to)	-4.6	-2.3	-2.2
Acc31457.1	Probable inactive purple acid phosphatase 1, Precursor (putative)	5.2	3.1	2.1
Acc32047.1	F-box/FBD/LRR-repeat protein At5g22660 (probable)	-5.8	-2.9	-2.9
Acc32442.1	Photosystem II 5 kDa protein, chloroplastic, Precursor [Petunia x hybrida] (probable)	4.5	2.4	2.1

Acc32565.1	Acyl-protein thioesterase 2 (APT-2) (probable)	5.0	2.4	2.5
Acc32781.1	Photosystem II protein psbY-2, chloroplastic, Precursor (putative)	5.4	3.0	2.4
Acc33159.1	Protein transport protein YIP1 (probable)	-6.8	-2.9	-4.0
Acc33160.1	Protein transport protein YIP1 (probable)	-6.8	-2.9	-3.9
Acc33184.1	Transcription factor MYB108 (AtMYB108) (putative)	-5.3	-2.7	-2.6
Acc33262.1	Long-chain-alcohol O-fatty-acyltransferase (probable)	6.3	2.3	4.0

Supplementary Table 6: Differentially expressed genes (DEGs) encoding transcription factors in the MYB, bHLH, homeobox and NAC families that are possibly involved in the flavonoid and anthocyanin pathway obtained from the comparison between mature green (MG), colour change (CC), and ripe (RP) stage in *A. purpurea* skin and flesh. Base mean are shown as an indication of expression.

Gene models	Skin comparison	MG-RP	MG-CC	CC-RP	Base mean
Acc00493.1	Transcription factor MYB114 AcMYB10	6.2	5.8	0.4	284
Acc01812.1	Transcription factor bHLH140	-5.2	-6	0.8	29
Acc03485.1	Homeobox ANTHOCYANINLESS2	-2.5	-4.3	1.8	78
Acc06303.1	Homeobox-leucine zipper protein HOX16	-4.3	-5.2	0.9	335
Acc06350.1	NAC transcription factor 29	-3.7	-3.7	-0.1	522
Acc06941.1	Transcription factor bHLH95	-4.4	-2.9	-1.5	69
Acc07314.1	bZIP transcription factor 44	5	4.2	0.8	226
Acc07545.1	Transcription factor bHLH14	2.5	4	-1.5	49
Acc07972.1	Homeobox-leucine zipper protein ATHB-6	7.5	7.6	-0.2	501
Acc10232.1	Transcription factor MYB114 AcMYB110	-2.4	-2.3	-0.2	1222
Acc10691.1	Transcription factor PIF3 (AtbHLH8)	2.8	2.3	0.5	45
Acc10801.1	Myb-related protein 308	6.3	6	0.3	20
Acc12804.1	Transcription factor TT2 (AtMYB123)	-5.9	-4.2	-1.7	12
Acc12965.1	Anthocyanin regulatory C1 protein, AcMYBC1	0.2	-0.53	0.72	63
Acc13843.1	Transcription factor bHLH36	5.6	4.6	0.9	161
Acc14402.1	Homeobox-leucine zipper protein HAT14	3	5.6	-2.6	58
Acc22908.1	Transcription factor MYB108	-11.6	-8.7	-2.9	268
Acc16887.1	WRKY transcription factor 44 (probable) WRKY44	0.09	-1.26	1.34	237
Acc31505.1	NAC transcription factor 29	-5.9	-5.2	-0.8	970
	**Flesh comparison**	**MG-RP**	**MG-CC**	**CC-RP**	**Base mean**
Acc00344.1	Transcription factor bHLH113	2.2	2.4	-0.2	1262
Acc00493.1	Transcription factor MYB114 AcMYB10	7.1	5.7	1.4	284
Acc00505.1	Homeobox-leucine zipper protein HAT22	-2.8	-2.4	-0.4	110
Acc00767.1	NAC domain-containing protein 100	-3.7	-2.8	-0.8	37
Acc01812.1	Transcription factor bHLH140	-7	-8.2	1.2	30
Acc01838.1	Probable WRKY transcription factor 33	3.9	2.3	1.6	100
Acc01879.1	Probable WRKY transcription factor 70	4	3.4	0.6	85
Acc02186.1	Myb-related protein 3R-1	-2	-2.2	0.2	237
Acc02780.1	Probable WRKY transcription factor 70	1	3.7	-2.7	101
Acc02825.1	Transcription factor bHLH51	4	3.8	0.2	18
Acc03057.1	Probable WRKY transcription factor 70	2.8	2.8	0	244
Acc04610.1	bZIP transcription factor 11	3.6	3.4	0.2	42
Acc04695.1	Transcription factor MYB44	-3.9	-7.6	3.7	26
Acc04749.1	Probable WRKY transcription factor 15	2.2	3.2	-1	157
Acc04751.1	Transcription factor bHLH155	2.9	3.1	-0.1	31
Acc04806.1	Transcription factor bHLH74	4.3	3.6	0.7	328
Acc05417.1	Homeobox-leucine zipper protein ATHB-16	8.7	8.1	0.6	577
Acc06381.1	Probable WRKY transcription factor 57	-4.2	-3	-1.3	141

Acc06525.1	Probable WRKY transcription factor 65	-2.1	0.1	-2.2	77
Acc06559.1	Transcription factor bHLH30	-2.5	0.3	-2.8	93
Acc06621.1	Transcription factor bHLH149	-7.1	-1	-6.1	264
Acc06941.1	Transcription factor bHLH95	-4.7	-2.8	-2	69
Acc07066.1	bZIP transcription factor 44	3.4	3.7	-0.3	77
Acc07314.1	bZIP transcription factor 44	4.4	3.1	1.3	226
Acc07548.1	Homeobox-leucine zipper protein ATHB-12	2.3	2.4	-0.1	382
Acc07972.1	Homeobox-leucine zipper protein ATHB-6	7.2	6.6	0.6	501
Acc09204.1	Probable WRKY transcription factor 53	3.4	1.2	2.2	200
Acc09734.1	WRKY transcription factor 6	-3.7	-3	-0.6	114
Acc09787.1	Transcription factor bHLH35	3.1	3.2	-0.1	72
Acc09989.1	Probable WRKY transcription factor 40	3.2	2.2	0.9	86
Acc10125.1	NAC domain-containing protein 100	-4.3	-3.1	-1.2	218
Acc10232.1	Transcription factor MYB114 AcMYB110	-4.2	-4.2	-0.1	1222
Acc10243.1	Homeobox-leucine zipper protein HAT22	-3	-2.5	-0.5	493
Acc10693.1	Transcription factor MYB86 (AtMyb4)	-2.5	-2.1	-0.5	402
Acc11919.1	Homeobox protein XHOX-3	5.1	4.1	1	60
Acc12299.1	NAC domain-containing protein 2	-2.7	-2.1	-0.6	673
Acc12364.1	Probable WRKY transcription factor 50	6	6.7	-0.7	27
Acc12804.1	Transcription factor TT2 (AtMYB123)	-4.7	-3.8	-0.9	12
Acc12965.1	Anthocyanin regulatory C1 protein, AcMYBC1	-1.2	-2.15	0.95	63
Acc13117.1	Myb family transcription factor EFM	-6.9	-5	-1.9	36
Acc13639.1	Probable WRKY transcription factor 31	3.7	2.2	1.5	49
Acc13843.1	Transcription factor bHLH36	5.8	6.5	-0.7	161
Acc15830.1	Transcription factor MYB1R1	-3.5	-2.5	-1	1320
Acc16347.1	NAC domain-containing protein 35	2.7	2.1	0.6	50
Acc16725.1	Homeobox-leucine zipper protein HAT4	3.9	0.7	3.1	45
Acc16887.1	WRKY transcription factor 44 (probable) WRKY44	-0.03	-1.64	1.61	237
Acc17473.1	WRKY transcription factor 6	-4.2	-3.9	-0.3	2857
Acc18287.1	NAC domain-containing protein 100	-2.1	-2.1	0	357
Acc18805.1	Transcription factor PIF3	4.4	1.6	2.8	122
Acc18978.1	Transcription factor bHLH36	5.4	4.2	1.2	20
Acc19394.1	WRKY transcription factor 6	5.6	4.5	1.2	59
Acc20968.1	Probable WRKY transcription factor 14	-4.7	-2.8	-1.9	40
Acc21211.1	NAC domain-containing protein 104	2	2.7	-0.6	20
Acc21466.1	Transcription factor bHLH149	-2.7	-2	-0.7	41
Acc21700.1	NAC transcription factor 56	-4.3	-5	0.6	4958
Acc22104.1	Transcription factor bHLH137	-3.2	-3.8	0.6	5103
Acc22141.1	Probable WRKY transcription factor 40	-2.2	-3.5	1.3	595
Acc22569.1	Homeobox-leucine zipper protein ATHB-8	-2.4	-3.1	0.7	71
Acc22908.1	Transcription factor MYB108	-9.1	-5.9	-3.2	267
Acc22928.1	Transcription factor bHLH35	2.3	-2.6	4.9	60
Acc23052.1	Myb-related protein 306	2.2	2.2	0	115
Acc23186.1	Transcription factor bHLH130	4.2	4.4	-0.2	29
Acc23259.1	Transcription factor bHLH68	2.4	2.7	-0.3	69

Acc23623.1	bZIP transcription factor 11	3	2.2	0.7	111
Acc23678.1	NAC domain-containing protein 78	3.1	2.4	0.7	279
Acc23769.1	Probable WRKY transcription factor 7	4.5	3.6	0.8	213
Acc23831.1	bZIP transcription factor 2	-5.5	-0.3	-5.2	12
Acc24523.1	Transcription factor MYB108	-6.2	-3.3	-2.9	197
Acc25144.1	Transcription factor bHLH30	3.4	2.5	0.9	35
Acc26399.1	NAC transcription factor 56	-4	-4.4	0.4	8183
Acc26849.1	Transcription factor bHLH137	-2.4	-2.9	0.5	284
Acc27100.1	Transcription factor MYB108	-4.4	0	-4.4	102
Acc27905.1	Transcription factor MYB1R1	-3.8	0.2	-4	101
Acc28785.1	Probable WRKY transcription factor 53	2.2	-0.8	3	125
Acc29099.1	Homeobox protein ATH1	-5.4	-4.5	-0.9	36
Acc29560.1	Probable WRKY transcription factor 40	-2.9	-3.6	0.7	307
Acc29877.1	NAC domain-containing protein 100	-2.5	-2.8	0.3	551
Acc30044.1	NAC domain-containing protein 90	6.6	6.4	0.2	43
Acc30416.1	Transcription factor bHLH130	3.6	3.4	0.2	32
Acc30717.1	Myb-related protein 306	-4	-1.1	-2.9	20
Acc30738.1	NAC domain-containing protein 100	-4.1	-3	-1.1	483
Acc30882.1	WD repeat-containing protein 17	4.9	6.5	-1.7	20
Acc31204.1	Transcription repressor MYB4	-2.8	-2.9	0.1	191
Acc31237.1	Transcription factor bHLH49	2.2	2.2	0	20
Acc31419.1	Transcription factor BPE	5.2	2.5	2.7	27
Acc31505.1	NAC transcription factor 29	-5.7	-4.8	-0.9	970
Acc33184.1	Transcription factor MYB108	-5.3	-2.7	-2.6	31

Supplementary Table 7. Pearson's correlation coefficient comparing the correlation between metabolite concentration and relative gene expression in callus over-expressing *MYB110, MYB5,* and *WRKY44*. Output calculated and produced from R studio.

	MYB110.expression	MYBC1 expression	WRKY44.expression	CHS.expression	DFR.expression	F3GT.expression	F3.H1.expression	F3.H2.expression	F3.5.H.expression	FLS1.expression	FLS2.expression	FLS3.expression	LAR1.expression	LAR2.expression	LAR3.expression	LAR4.expression	LAR5.expression	ANR2.expression	ANR1.expression	Procyanidin B1	Procyanidin B3	B.type.procyanidin.dimer.C	B.type.procyanidin.dimer.D	C.type.procyanidin.trimer.A	Procyanidin C1	C.type.procyanidin.trimer.C	Catechin	Epicatechin	cyanidin.3.galactoside
MYB110.expression	1	-0.3027189	-0.2862309	-0.1352285	-0.3649706	0.9322926	-0.370839	-0.405793	-0.3258145	-0.2287808	-0.1230027	-0.1131535	-0.461411	0.8190078	-0.5318775	-0.1413176	-0.4483355	-0.4343696	-0.3635224	-0.0884294	-0.3949854	-0.5943559	-0.2360583	-0.4246198	0.0225739	0.5492572	-0.2400659	0.1437086	0.9360492
cyanidin.3.galactoside	0.9360492	-0.3149089	-0.3058111	-0.1372344	-0.3756142	0.9370899	-0.3840568	-0.4364267	-0.3519727	-0.2672024	-0.1252657	-0.1518272	-0.4744518	0.8528619	-0.5345439	-0.1424134	-0.4574493	-0.4399391	-0.3614521	-0.0808817	-0.4386046	-0.6065198	-0.2463907	-0.4661842	0.0324997	0.5817801	-0.2700546	0.1387484	1
F3GT.expression	0.9322926	-0.2921066	-0.2651526	-0.131424	-0.3260229	1	-0.3220966	-0.3820047	-0.3007024	-0.1960899	-0.1195544	-0.0152689	-0.4079572	0.9223906	-0.4720499	-0.1368566	-0.4011475	-0.3844277	-0.3211618	-0.0862221	-0.3777443	-0.5811211	-0.2700248	-0.406195	0.0308641	0.5241404	-0.2143676	0.1776367	0.9370899
LAR2.expression	0.8190078	0.0054093	-0.4123082	-0.0184639	-0.3421173	0.9223906	-0.2782603	-0.0843681	-0.4131034	-0.2716004	-0.0057107	-0.0167015	-0.4408875	1	-0.4405995	-0.0182688	-0.4086924	-0.3595983	-0.3957695	0.1466638	-0.2222302	-0.391289	-0.0993148	-0.2541345	0.2275735	0.6447075	-0.0925529	0.3222665	0.8528619
C.type.procyanidin.trimer.C	0.5492572	0.2964554	-0.5641286	0.1077509	-0.3651819	0.5241404	-0.1060762	-0.0086926	-0.5689871	-0.6296358	0.1187493	-0.4243658	-0.6840143	0.6447075	-0.3995877	0.1238564	-0.390669	-0.3342139	-0.4231831	0.7503588	0.3319978	0.2281083	0.4726892	0.2689148	0.7809401	1	0.4551534	0.7890749	0.5817801
Epicatechin	0.1437086	0.275772	-0.4562971	0.1962368	-0.2307041	0.1776367	0.0932874	0.0333671	-0.5271132	-0.6481217	0.2046464	-0.3280808	-0.5500522	0.3222665	-0.2031954	0.2174286	-0.201435	-0.186194	-0.2782697	0.8334464	0.5849589	0.5593958	0.6080696	0.4677594	0.7872736	0.7890749	0.739602	1	0.1387484
Procyanidin C1	0.0225739	0.5935938	-0.2278144	0.3433338	-0.0910906	0.0308641	0.2047233	0.3011453	-0.2462778	-0.3112455	0.3434242	-0.4888116	-0.3761597	0.2275735	-0.0798566	0.3641352	-0.0021251	0.003666	-0.1193307	0.9607689	0.8181494	0.7467474	0.5920589	0.7910897	1	0.7809401	0.832111	0.7872736	0.0324997
Procyanidin B1	-0.0884294	0.6688277	-0.4059715	0.2761651	-0.1518342	-0.0862221	0.1743594	0.3949367	-0.3656619	-0.4739067	0.2795219	-0.4238037	-0.4447097	0.1466638	-0.0901224	0.2998517	-0.109781	-0.0563404	-0.2190085	1	0.788957	0.7757737	0.7489166	0.7429142	0.9607689	0.7503588	0.7850709	0.8334464	-0.0808817
FLS3.expression	-0.1131535	3.136E-05	0.0807253	-0.6522824	0.4916788	-0.0152689	0.3714904	-0.0354529	0.3386344	0.0913653	-0.6564087	1	0.5831309	-0.0167015	0.651593	-0.653735	0.3678408	0.5335788	0.4084614	-0.4238037	-0.268112	-0.3082741	-0.3963334	-0.2524567	-0.4888116	-0.4243658	-0.3753932	-0.3280808	-0.1518273
FLS2.expression	-0.1230027	-0.0537552	0.1324302	0.9996329	-0.2444016	-0.1195544	-0.1214798	0.2497143	-0.310081	0.1966874	1	-0.6564087	-0.2765131	-0.0057107	-0.3652295	0.9993516	-0.029796	-0.3091033	-0.2022156	0.2795219	0.2824327	0.393609	0.267899	0.260126	0.3434242	0.1187493	0.4313963	0.2046464	-0.1252657
CHS.expression	-0.1352285	-0.051111	0.1523274	1	-0.2203062	-0.131424	-0.101387	0.2466	-0.2927689	0.2142079	0.9996329	-0.6522824	-0.253825	-0.0184639	-0.3482561	0.999456	-0.0055842	-0.2862193	-0.1777296	0.2761651	0.2911302	0.4020941	0.2589285	0.2713145	0.3433338	0.1077509	0.4366026	0.1962368	-0.1372344
LAR4.expression	-0.1413176	-0.0351594	0.1419347	0.999456	-0.2269116	-0.1368566	-0.0962512	0.2602877	-0.2970206	0.196024	0.9993516	-0.653735	-0.2644779	-0.0182688	-0.3405067	1	-0.0066651	-0.2845934	-0.1826118	0.2998517	0.3106392	0.4219534	0.2759554	0.2889522	0.3641352	0.1238564	0.4557907	0.2174286	-0.1424134
FLS1.expression	-0.2287808	-0.1459305	0.8597405	0.2142079	0.4254767	-0.1960899	0.1801067	0.0139506	0.7400974	1	0.1966874	0.0913653	0.6957304	-0.2716004	0.2720601	0.196024	0.556528	0.4083722	0.5237372	-0.4739067	0.033994	-0.0603024	-0.6596048	0.1288108	-0.3112455	-0.6296358	-0.0784918	-0.6481217	-0.2672024
B.type.procyanidin.dimer.D	-0.2360583	0.499406	-0.6814339	0.2589285	-0.2989576	-0.2700248	-0.0318472	0.3542573	-0.6559483	-0.6596048	0.267899	-0.3963334	-0.5999813	-0.0993148	-0.261696	0.2759554	-0.3935243	-0.3266844	-0.4314651	0.7489166	0.3949594	0.5464855	1	0.3529398	0.5920589	0.4726892	0.4000289	0.6080696	-0.2463907
Catechin	-0.2400659	0.3087252	0.1502298	0.4366026	0.0844603	-0.2143676	0.3568079	0.1456866	0.0008199	-0.0784918	0.4313963	-0.3753932	-0.1124866	-0.0925529	0.1511911	0.4557907	0.2913152	0.1885209	0.1248473	0.7850709	0.9341496	0.8805871	0.4000289	0.8745846	0.832111	0.4551534	1	0.739602	-0.2700546
WRKY44.expression	-0.2862309	-0.3183511	1	0.1523274	0.5315321	-0.2651526	0.3736249	-0.2039274	0.8298764	0.8597405	0.1324302	0.0807253	0.7355634	-0.4123082	0.4802453	0.1419347	0.7584049	0.5758232	0.7133159	-0.4059715	0.1900458	0.1055841	-0.6814339	0.2526223	-0.2278144	-0.5641286	0.1502298	-0.4562971	-0.3058111
MYBC1 expression	-0.3027189	1	-0.3183511	-0.051111	0.0922412	-0.2921066	0.2172643	0.7734439	0.000173	-0.1459305	-0.0537552	3.136E-05	0.0143721	0.0054093	0.2046503	-0.0351594	0.0405537	0.2324098	-0.0318492	0.6688277	0.550371	0.5484494	0.499406	0.6028242	0.5935938	0.2964554	0.3087252	0.275772	-0.3149089
F3.5.H.expression	-0.3258145	0.000173	0.8298764	-0.2927689	0.4981951	-0.3007024	0.3228195	0.012001	1	0.7400974	-0.310081	0.3386344	0.7885764	-0.4131034	0.6540845	-0.2970206	0.6682316	0.6628683	0.6535807	-0.3656619	0.19475	0.0548266	-0.6559483	0.2892818	-0.2462778	-0.5689871	0.0008199	-0.5271132	-0.3519727
ANR1.expression	-0.3635224	-0.0318492	0.7133159	-0.1777296	0.9486637	-0.3211618	0.8646993	-0.2320084	0.6535807	0.5237372	-0.2022156	0.4084614	0.8619428	-0.3957695	0.749801	-0.1826118	0.9457647	0.9277396	1	-0.2190085	0.2167498	0.194483	-0.4314651	0.2810341	-0.1193307	-0.4231831	0.1248473	-0.2782697	-0.3614521
DFR.expression	-0.3649706	0.0922412	0.5315321	-0.2203062	1	-0.3260229	0.9009838	-0.1893678	0.498195	0.4254767	-0.2444016	0.4916787	0.8450263	-0.3421173	0.7362374	-0.2269116	0.8941575	0.9287895	0.9486637	-0.1518342	0.1948203	0.1934284	-0.2989576	0.2572255	-0.0910906	-0.3651819	0.0844603	-0.2307041	-0.3756142
F3.H1.expression	-0.370839	0.2172642	0.3736249	-0.101387	0.9009838	-0.3220966	1	-0.0806737	0.3228195	0.1801067	-0.1214798	0.3714904	0.6222363	-0.2782603	0.694616	-0.0962512	0.8381544	0.8765198	0.8646993	0.1743594	0.4317426	0.4468017	-0.0318472	0.4568338	0.2047233	-0.1060762	0.3568079	0.0932874	-0.3840568
Procyanidin B3	-0.3949854	0.550371	0.1900458	0.2911302	0.1948203	-0.3777443	0.4317426	0.3473849	0.19475	0.033994	0.2824327	-0.268112	0.0566912	-0.2222302	0.3021515	0.3106392	0.3724659	0.3448082	0.2167498	0.788957	1	0.9365498	0.3949594	0.9837008	0.8181494	0.3319978	0.9341496	0.5849589	-0.4386046
F3.H2.expression	-0.405793	0.7734439	-0.2039274	0.2466	-0.1893678	-0.3820047	-0.0806737	1	0.012001	0.0139506	0.2497143	-0.0354529	-0.0166533	-0.0843681	0.1068259	0.2602877	-0.0711099	-0.0018926	-0.2320084	0.3949367	0.3473849	0.4360421	0.3542573	0.3911909	0.3011453	-0.0086926	0.1456866	0.0333671	-0.4364267
C.type.procyanidin.trimer.A	-0.4246198	0.6028242	0.2526223	0.2713145	0.2572255	-0.406195	0.4568338	0.391191	0.2892818	0.1288108	0.260126	-0.2524567	0.1430732	-0.2541345	0.362227	0.2889522	0.4327507	0.4145525	0.2810341	0.7429142	0.9837008	0.9149574	0.3529398	1	0.7910897	0.2689148	0.8745846	0.4677595	-0.4661842
ANR2.expression	-0.4343696	0.2324098	0.5758232	-0.2862193	0.9287896	-0.3844277	0.8765198	-0.0018926	0.6628683	0.4083722	-0.3091033	0.5335788	0.872674	-0.3595983	0.8971292	-0.2845934	0.9382158	1	0.9277396	-0.0563404	0.3448082	0.3136631	-0.3266844	0.4145525	0.003666	-0.3342139	0.1885209	-0.186194	-0.4399391
LAR5.expression	-0.4483355	0.0405537	0.7584049	-0.0055842	0.8941575	-0.4011475	0.8381544	-0.0711099	0.6682316	0.556528	-0.029796	0.3678408	0.8645444	-0.4086924	0.8283324	-0.0066651	1	0.9382158	0.9457647	-0.109781	0.3724659	0.3584578	-0.3935243	0.4327507	-0.0021251	-0.390669	0.2913152	-0.201435	-0.4574493
LAR1.expression	-0.461411	0.0143721	0.7355634	-0.253825	0.8450263	-0.4079572	0.6222363	-0.0166533	0.7885764	0.6957304	-0.2765131	0.5831309	1	-0.4408875	0.7923843	-0.2644779	0.8645444	0.872674	0.8619428	-0.4447097	0.0566912	0.0393703	-0.5999813	0.1430732	-0.3761597	-0.6840143	-0.1124866	-0.5500522	-0.4744518
LAR3.expression	-0.5318775	0.2046503	0.4802453	-0.3482561	0.7362374	-0.4720499	0.694616	0.1068259	0.6540845	0.2720601	-0.3652294	0.651593	0.7923843	-0.4405995	1	-0.3405067	0.8283324	0.8971292	0.749801	-0.0901224	0.3021515	0.3023623	-0.261696	0.362227	-0.0798566	-0.3995877	0.1511911	-0.2031954	-0.5345439
B.type.procyanidin.dimer.C	-0.5943559	0.5484494	0.1055841	0.4020941	0.1934284	-0.5811211	0.4468017	0.4360421	0.0548266	-0.0603024	0.393609	-0.3082741	0.0393703	-0.391289	0.3023623	0.4219534	0.3584578	0.3136631	0.194483	0.7757737	0.9365498	1	0.5464855	0.9149574	0.7467473	0.2281083	0.8805871	0.5593958	-0.6065198

Supplementary Table 8. P-value to indicate the statistical significance of Pearson's correlation coefficient comparing the correlation between metabolite concentration and relative gene expression in callus over-expressing *MYB110, MYB5,* and *WRKY44*. Output calculated and produced from R studio.

	MYB110.expression	MYBC1expression	WRKY44.expression	CHS.expression	DFR.expression	F3GT.expression	F3.H1.expression	F3.H2.expression	F3.5.H.expression	FLS1.expression	FLS2.expression	FLS3.expression	LAR1.expression
MYB110.expression	NA	0.2727717	3.01E-01	0.6308585	1.81E-01	4.18E-07	1.74E-01	0.1334236	0.2359835	4.12E-01	0.6623117	0.6880402	8.34E-02
MYBC1 expression	2.73E-01	NA	2.48E-01	0.8564494	7.44E-01	2.91E-01	4.37E-01	0.0007183	0.9995117	6.04E-01	0.8490996	0.9999115	9.59E-01
WRKY44.expression	3.01E-01	0.2475153	NA	0.5878464	4.14E-02	3.40E-01	1.70E-01	0.4659994	0.0001291	3.97E-05	0.6380083	0.7748853	1.78E-03
CHS.expression	6.31E-01	0.8564494	5.88E-01	NA	4.30E-01	6.41E-01	7.19E-01	0.3756079	0.2896238	4.43E-01	0	0.0084016	3.61E-01
DFR.expression	1.81E-01	0.7437028	4.14E-02	0.4301195	NA	2.36E-01	4.58E-06	0.4990736	0.0587526	1.14E-01	0.3800153	0.0626738	7.32E-05
F3GT.expression	4.18E-07	0.2907669	3.40E-01	0.6405866	2.36E-01	NA	2.42E-01	0.1599873	0.2761383	4.84E-01	0.6712814	0.9569285	1.31E-01
F3.H1.expression	1.74E-01	0.4366742	1.70E-01	0.7191965	4.58E-06	2.42E-01	NA	0.7750257	0.2405702	5.21E-01	0.6662678	0.1727726	1.32E-02
F3.H2.expression	1.33E-01	0.0007183	4.66E-01	0.3756079	4.99E-01	1.60E-01	7.75E-01	NA	0.9661411	9.61E-01	0.3694119	0.9001795	9.53E-01
F3.5.H.expression	2.36E-01	0.9995117	1.29E-04	0.2896238	5.88E-02	2.76E-01	2.41E-01	0.9661411	NA	1.61E-03	0.2606911	0.2169682	4.77E-04
FLS1.expression	4.12E-01	0.6037981	3.97E-05	0.4433106	1.14E-01	4.84E-01	5.21E-01	0.9606445	0.0016062	NA	0.4823106	0.7460624	3.97E-03
FLS2.expression	6.62E-01	0.8490996	6.38E-01	0	3.80E-01	6.71E-01	6.66E-01	0.3694119	0.2606911	4.82E-01	NA	0.0078631	3.18E-01
FLS3.expression	6.88E-01	0.9999115	7.75E-01	0.0084016	6.27E-02	9.57E-01	1.73E-01	0.9001795	0.2169682	7.46E-01	0.0078631	NA	2.25E-02
LAR1.expression	8.34E-02	0.9594563	1.78E-03	0.3613198	7.32E-05	1.31E-01	1.32E-02	0.953027	0.0004768	3.97E-03	0.3184473	0.0225087	NA
LAR2.expression	1.88E-04	0.9847354	1.27E-01	0.9479262	2.12E-01	9.91E-07	3.15E-01	0.7649844	0.1259038	3.27E-01	0.9838849	0.9528912	1.00E-01
LAR3.expression	4.13E-02	0.4643858	7.00E-02	0.2033531	1.75E-03	7.56E-02	4.06E-03	0.7047407	0.008163	3.27E-01	0.1806958	0.0084943	4.28E-04
LAR4.expression	6.15E-01	0.901002	6.14E-01	0	4.16E-01	6.27E-01	7.33E-01	0.3487974	0.282349	4.84E-01	0	0.0082088	3.41E-01
LAR5.expression	9.37E-02	0.8858989	1.05E-03	0.9842418	6.95E-06	1.38E-01	9.54E-05	0.8011704	0.006469	3.12E-02	0.9160502	0.1773628	3.21E-05
ANR2.expression	1.06E-01	0.4045435	2.47E-02	0.301045	5.76E-07	1.57E-01	1.81E-05	0.994659	0.007075	1.31E-01	0.2622764	0.040513	2.19E-05
ANR1.expression	1.83E-01	0.9102861	2.83E-03	0.5262738	7.20E-08	2.43E-01	3.18E-05	0.4053786	0.0082291	4.51E-02	0.4698316	0.1306442	3.60E-05
Procyanidin B1	7.54E-01	0.0064043	1.33E-01	0.3190817	5.89E-01	7.60E-01	5.34E-01	0.1451449	0.180141	7.43E-02	0.3129921	0.1154279	9.67E-02
Procyanidin B3	1.45E-01	0.0335112	4.98E-01	0.2924569	4.87E-01	1.65E-01	1.08E-01	0.2045627	0.4867211	9.04E-01	0.3077664	0.3339662	8.41E-01
B.type.procyanidin.dimer.C	1.95E-02	0.0342627	7.08E-01	0.1373424	4.90E-01	2.31E-02	9.50E-02	0.104203	0.8461246	8.31E-01	0.1466244	0.2636254	8.89E-01
B.type.procyanidin.dimer.D	3.97E-01	0.0580441	5.15E-03	0.3514107	2.79E-01	3.30E-01	9.10E-01	0.1951446	0.0079218	7.47E-03	0.3343651	0.1435994	1.81E-02
C.type.procyanidin.trimer.A	1.15E-01	0.0173754	3.64E-01	0.3280007	3.55E-01	1.33E-01	8.69E-02	0.1493448	0.2956723	6.47E-01	0.3491078	0.3640025	6.11E-01
Procyanidin C1	9.36E-01	0.0196551	4.14E-01	0.2102483	7.47E-01	9.13E-01	4.64E-01	0.2753968	0.376252	2.59E-01	0.2101203	0.0644579	1.67E-01
C.type.procyanidin.trimer.C	3.39E-02	0.2833098	2.85E-02	0.7022913	1.81E-01	4.49E-02	7.07E-01	0.9754723	0.0268555	1.19E-02	0.6733815	0.1148949	4.92E-03
Catechin	3.89E-01	0.2628911	5.93E-01	0.1037079	7.65E-01	4.43E-01	1.92E-01	0.6044096	0.9976863	7.81E-01	0.1083704	0.1679511	6.90E-01
Epicatechin	6.09E-01	0.3197993	8.73E-02	0.4833347	4.08E-01	5.26E-01	7.41E-01	0.9060275	0.0434801	8.97E-03	0.4643945	0.232549	3.36E-02
cyanidin.3.galactoside	2.91E-07	0.2529486	2.68E-01	0.625752	1.68E-01	2.62E-07	1.58E-01	0.1038632	0.1982438	3.36E-01	0.6564481	0.5890873	7.40E-02

	LAR2.expression	LAR3.expression	LAR4.expression	LAR5.expression	ANR2.expression	ANR1.expression	Procyanidin B1	Procyanidin B3	B.type.procyanidin.dimer.C	B.type.procyanidin.dimer.D	C.type.procyanidin.trimer.A	Procyanidin C1	C.type.procyanidin.trimer.C	Catechin	Epicatechin	cyanidin.3.galactoside
MYB110.expression	1.88E-04	4.13E-02	0.6154059	9.37E-02	1.06E-01	1.83E-01	7.54E-01	1.45E-01	1.95E-02	0.3969944	1.15E-01	9.36E-01	0.0339453	3.89E-01	0.6093786	2.91E-07
MYBC1 expression	9.85E-01	4.64E-01	0.901002	8.86E-01	4.05E-01	9.10E-01	6.40E-03	3.35E-02	3.43E-02	0.0580441	1.74E-02	1.97E-02	0.2833098	2.63E-01	0.3197993	2.53E-01
WRKY44.expression	1.27E-01	7.00E-02	0.6138479	1.05E-03	2.47E-02	2.83E-03	1.33E-01	4.98E-01	7.08E-01	0.0051506	3.64E-01	4.14E-01	0.0284864	5.93E-01	0.0873373	2.68E-01
CHS.expression	9.48E-01	2.03E-01	0	9.84E-01	3.01E-01	5.26E-01	3.19E-01	2.92E-01	1.37E-01	0.3514107	3.28E-01	2.10E-01	0.7022913	1.04E-01	0.4833347	6.26E-01
DFR.expression	2.12E-01	1.75E-03	0.4160608	6.95E-06	5.76E-07	7.20E-08	5.89E-01	4.87E-01	4.90E-01	0.2790714	3.55E-01	7.47E-01	0.1807569	7.65E-01	0.4080985	1.68E-01
F3GT.expression	9.91E-07	7.56E-02	0.6267125	1.38E-01	1.57E-01	2.43E-01	7.60E-01	1.65E-01	2.31E-02	0.3303957	1.33E-01	9.13E-01	0.0448971	4.43E-01	0.5264937	2.62E-07
F3.H1.expression	3.15E-01	4.06E-03	0.7329284	9.54E-05	1.81E-05	3.18E-05	5.34E-01	1.08E-01	9.50E-02	0.9102918	8.69E-02	4.64E-01	0.7067277	1.92E-01	0.7408877	1.58E-01
F3.H2.expression	7.65E-01	7.05E-01	0.3487974	8.01E-01	9.95E-01	4.05E-01	1.45E-01	2.05E-01	1.04E-01	0.1951446	1.49E-01	2.75E-01	0.9754723	6.04E-01	0.9060275	1.04E-01
F3.5.H.expression	1.26E-01	8.16E-03	0.282349	6.47E-03	7.08E-03	8.23E-03	1.80E-01	4.87E-01	8.46E-01	0.0079218	2.96E-01	3.76E-01	0.0268555	9.98E-01	0.0434801	1.98E-01
FLS1.expression	3.27E-01	3.27E-01	0.4838187	3.12E-02	1.31E-01	4.51E-02	7.43E-02	9.04E-01	8.31E-01	0.007465	6.47E-01	2.59E-01	0.0118915	7.81E-01	0.0089734	3.36E-01
FLS2.expression	9.84E-01	1.81E-01	0	9.16E-01	2.62E-01	4.70E-01	3.13E-01	3.08E-01	1.47E-01	0.3343651	3.49E-01	2.10E-01	0.6733815	1.08E-01	0.4643944	6.56E-01
FLS3.expression	9.53E-01	8.49E-03	0.0082088	1.77E-01	4.05E-02	1.31E-01	1.15E-01	3.34E-01	2.64E-01	0.1435994	3.64E-01	6.45E-02	0.1148949	1.68E-01	0.232549	5.89E-01
LAR1.expression	1.00E-01	4.28E-04	0.3408097	3.21E-05	2.19E-05	3.60E-05	9.67E-02	8.41E-01	8.89E-01	0.0180546	6.11E-01	1.67E-01	0.0049198	6.90E-01	0.033635	7.40E-02
LAR2.expression	NA	1.00E-01	0.9484757	1.30E-01	1.88E-01	1.44E-01	6.02E-01	4.26E-01	1.49E-01	0.7247278	3.61E-01	4.15E-01	0.0094649	7.43E-01	0.2414231	5.33E-05
LAR3.expression	1.00E-01	NA	0.2142749	1.36E-04	5.81E-06	1.29E-03	7.49E-01	2.74E-01	2.73E-01	0.3461011	1.85E-01	7.77E-01	0.1400416	5.91E-01	0.4676362	4.01E-02
LAR4.expression	9.48E-01	2.14E-01	NA	9.81E-01	3.04E-01	5.15E-01	2.78E-01	2.60E-01	1.17E-01	0.3194645	2.96E-01	1.82E-01	0.6600976	8.77E-02	0.4363188	6.13E-01
LAR5.expression	1.30E-01	1.36E-04	0.9811922	NA	2.34E-07	1.02E-07	6.97E-01	1.72E-01	1.90E-01	0.1467191	1.07E-01	9.94E-01	0.1499364	2.92E-01	0.471584	8.64E-02
ANR2.expression	1.88E-01	5.81E-06	0.3039205	2.34E-07	NA	6.31E-07	8.42E-01	2.08E-01	2.55E-01	0.2346614	1.24E-01	9.90E-01	0.2234119	5.01E-01	0.5064254	1.01E-01
ANR1.expression	1.44E-01	1.29E-03	0.5147829	1.02E-07	6.31E-07	NA	4.33E-01	4.38E-01	4.87E-01	0.1083078	3.10E-01	6.72E-01	0.1160186	6.58E-01	0.3152558	1.86E-01
Procyanidin B1	6.02E-01	7.49E-01	0.277566	6.97E-01	8.42E-01	4.33E-01	NA	4.72E-04	6.76E-04	0.0013136	1.51E-03	1.29E-08	0.0012702	5.26E-04	0.0001135	7.74E-01
Procyanidin B3	4.26E-01	2.74E-01	0.2597886	1.72E-01	2.08E-01	4.38E-01	4.72E-04	NA	2.77E-07	0.1451197	4.52E-11	1.93E-04	0.2266871	3.51E-07	0.0219887	1.02E-01
B.type.procyanidin.dimer.C	1.49E-01	2.73E-01	0.1171948	1.90E-01	2.55E-01	4.87E-01	6.76E-04	2.77E-07	NA	0.0350438	1.76E-06	1.38E-03	0.4135398	1.47E-05	0.030146	1.65E-02
B.type.procyanidin.dimer.D	7.25E-01	3.46E-01	0.3194645	1.47E-01	2.35E-01	1.08E-01	1.31E-03	1.45E-01	3.50E-02	NA	1.97E-01	2.01E-02	0.0751813	1.40E-01	0.0161738	3.76E-01
C.type.procyanidin.trimer.A	3.61E-01	1.85E-01	0.2962477	1.07E-01	1.24E-01	3.10E-01	1.51E-03	4.52E-11	1.76E-06	0.196928	NA	4.44E-04	0.3324651	1.99E-05	0.0787011	7.99E-02
Procyanidin C1	4.15E-01	7.77E-01	0.1821044	9.94E-01	9.90E-01	6.72E-01	1.29E-08	1.93E-04	1.38E-03	0.0200554	4.44E-04	NA	0.0005886	1.19E-04	0.0004945	9.08E-01
C.type.procyanidin.trimer.C	9.46E-03	1.40E-01	0.6600976	1.50E-01	2.23E-01	1.16E-01	1.27E-03	2.27E-01	4.14E-01	0.0751813	3.32E-01	5.89E-04	NA	8.82E-02	0.0004701	2.29E-02
Catechin	7.43E-01	5.91E-01	0.0877337	2.92E-01	5.01E-01	6.58E-01	5.26E-04	3.51E-07	1.47E-05	0.1395638	1.99E-05	1.19E-04	0.0882344	NA	0.001624	3.30E-01
Epicatechin	2.41E-01	4.68E-01	0.4363188	4.72E-01	5.06E-01	3.15E-01	1.14E-04	2.20E-02	3.01E-02	0.0161738	7.87E-02	4.95E-04	0.0004701	1.62E-03	NA	6.22E-01
cyanidin.3.galactoside	5.33E-05	4.01E-02	0.6126407	8.64E-02	1.01E-01	1.86E-01	7.74E-01	1.02E-01	1.65E-02	0.3760262	7.99E-02	9.08E-01	0.022899	3.30E-01	0.6219081	NA

Supplementary Table 9. Oligonucleotide primers used for real-time qPCR analysis.

Gene Name	Primer sequence 5' - 3'
EF1a	GCACTGTCATTGATGCTCCT
EF1a	CCAGCTTCAAAACCACCAGT
MYB110	ATATCTCCAAGAGAAGAGCCGATATCCAA
MYB110	TGGTTGAATGCTATCTGCAATGACAGT
MYBC1	CCACTTACATCACAGCCCAGGAATA
MYBC1	GTGTTGGTGGTTTTTGGTGACGCTT
WRKY44	GAACCAGGCATTTTGGGGCAAGAG
WRKY44	TTGGTGCATCTGTAGTAACTTCTTGG
CHS	ACAGCTTGACCACCTAAATGGGCTTA
CHS	CAAAGTGGAATAAAGCATGGCCAAT
F3'5'H	GATTTTGTGCCGTCGATTGCGT
F3'5'H	AAGCTTGGGGCCATTAGGGTTTT
F3GT	TAGCCAAGCAGAGATCCGCTTCC
F3GT	CAAGAATCCTTCTGGTAAGTACTGTTTCGA
DFR	GTCGGAGAAGCTGATTGGGATGGG
DFR	TGGCATTTCCATTGGGAGGGGTT
F3'H1	GGTGGCGTATGCTCCGGAAGA
F3'H1	GTGGCCCGCACTCACCAGTG
F3'H2	CGAAACGCCTCGAATCGTACG
F3'H2	CTGATCACGTTCAGGCTCACTG
FLS1	GGGAGTGACAGAGGAATACAATGGG
FLS1	CTGTGGGCAAGGTGGGTACATA
FLS2	GTTCAAGTTGCTGTCACTAGGGTT
FLS2	GACTTCATTTGGGACAAGAATGGTGAG
FLS3	CATTTGGCCCAAGAACCCTCCTTC
FLS3	CTATTTCATCACCACCCAAACACGAC
LAR1	ATTACCTCCCTCTACCCAGAAGC
LAR1	TTTGCTCCGGGATTTGGTGTAACG
LAR2	GATGACATTCGTACGCTCAACAAATC
LAR2	GTGGGATTATATTCTCTTCTGCTGCA
LAR3	GCGCCATAGTCATACCCGGTTC
LAR3	GCAAAAACCTCTTCACAGTGCCAAC
LAR4	GGAGCAATCATCATACAACAGGGTG
LAR4	CTCCGATGGCAGGAACCTCTTAAC
LAR5	CAATCAAGAGGTTCCTGCCATCTG
LAR5	CTCTATCAGCCGCCTCACGC
ANR2	CTGTGTCGATCAATCAGCTCAAAGG

ANR2	CTTCGGCAAACTTCCACGCTTC
ANR1	CTGTGACGATCAATCAGCTCAACGA
ANR1	CTTCGGCAAACTTCCACGCTG
